# Supplementary figures and images for: Effects of a High-Fat Diet on Intestinal and Gonadal Metabolism in Female and Male Sea Cucumber Apostichopus japonicus
Source: Biology (Basel). 2023 Jan 29;12(2):212. doi: 10.3390/biology12020212 (PMC9953091; doi:10.3390/biology12020212)

Number of Tags

0 50000 100000 150000 200000 250000 300000

Total\_Tags(103666)  
Taxon\_Tags(94009)

Unclassified\_Tags(0)  
Singleton\_Tags(9656)

OTUs(355)

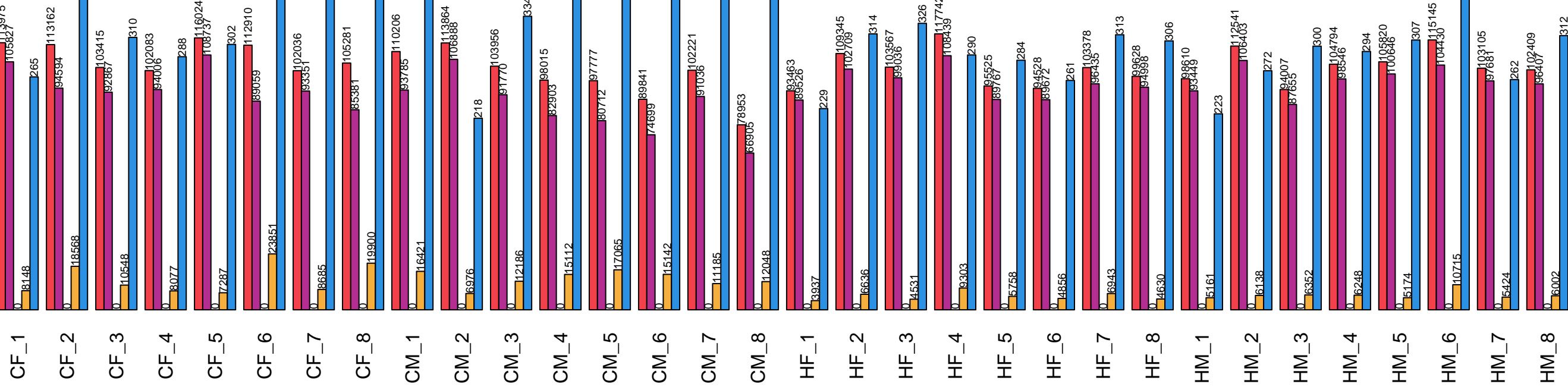

Number of OTUs

0 200 400 600 800

Supplement: Supplementary file 1 [file biology-12-00212-s001.zip › 16s rDNA SEQ/1.OTU/all.tags_otus_count.pdf]

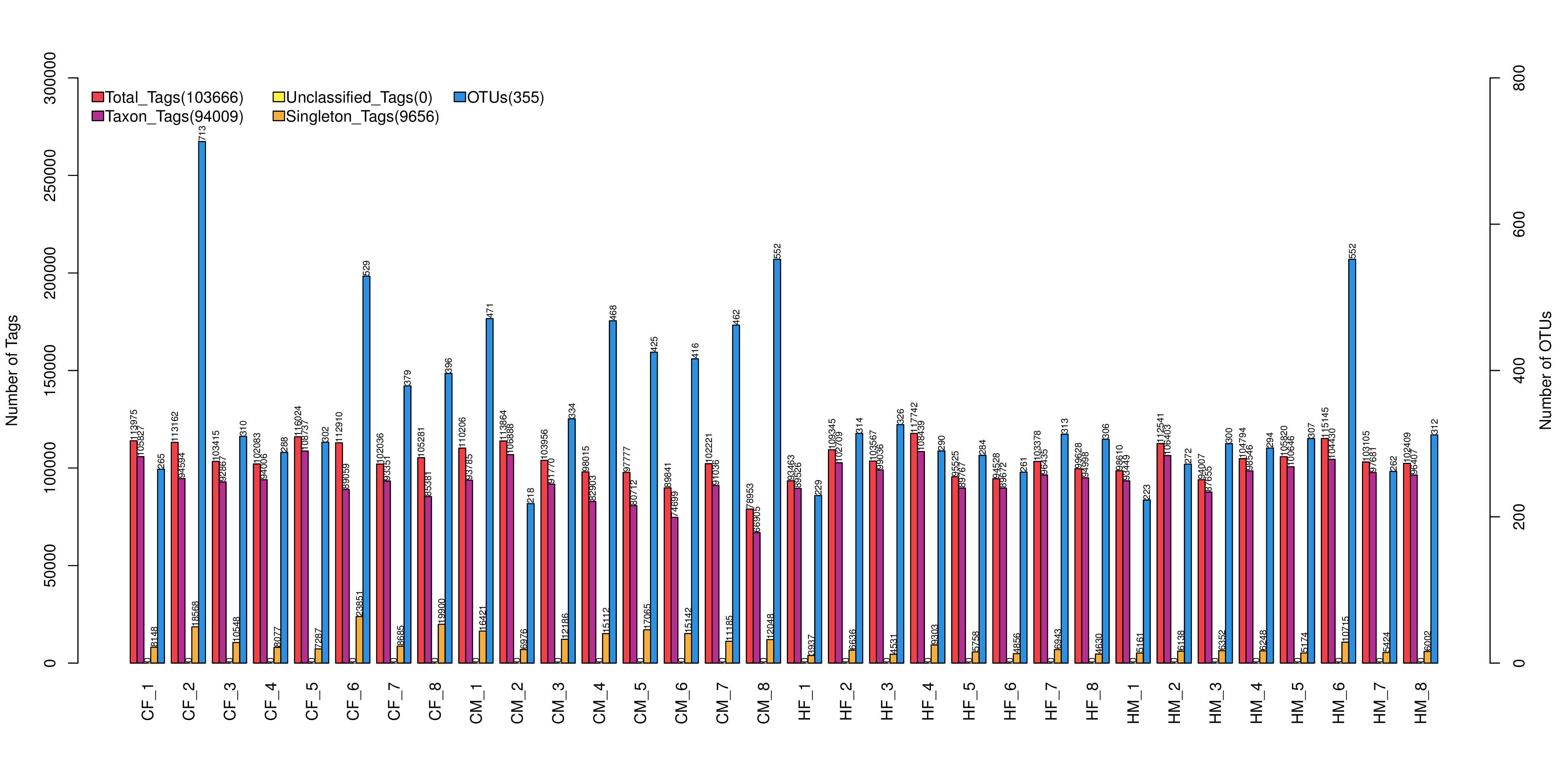

Supplement: Supplementary file 1 [file biology-12-00212-s001.zip › 16s rDNA SEQ/1.OTU/all.tags_otus_count.png]

CF HF

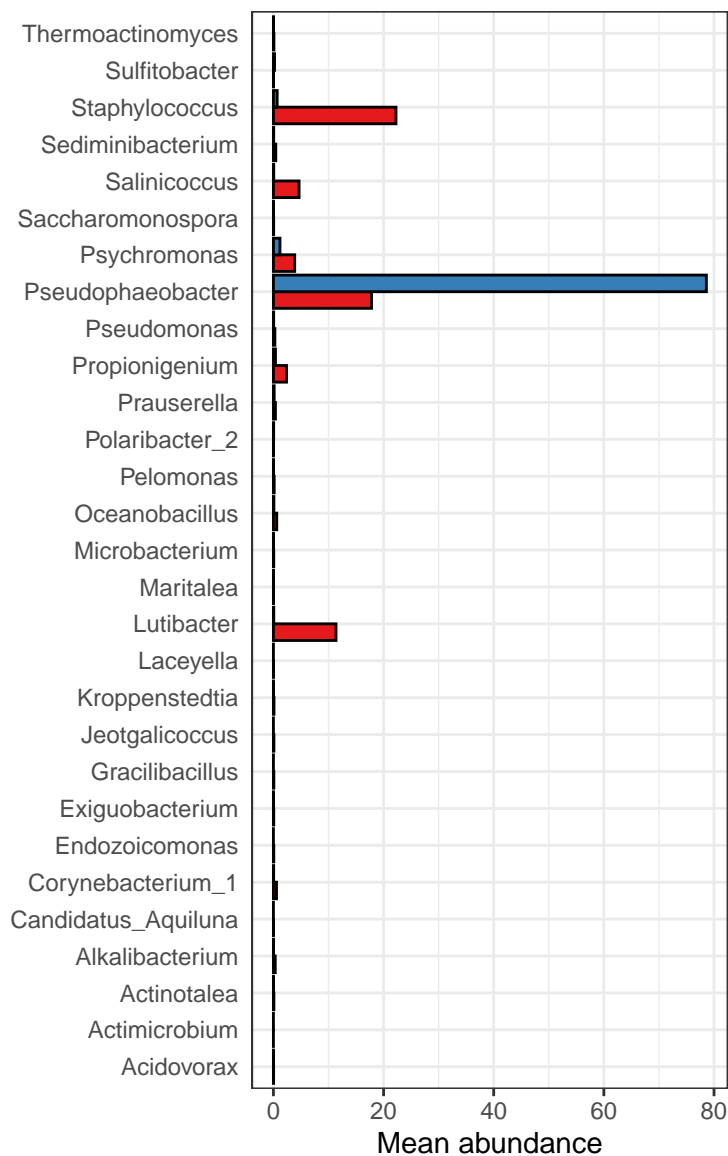

95% confidence intervals

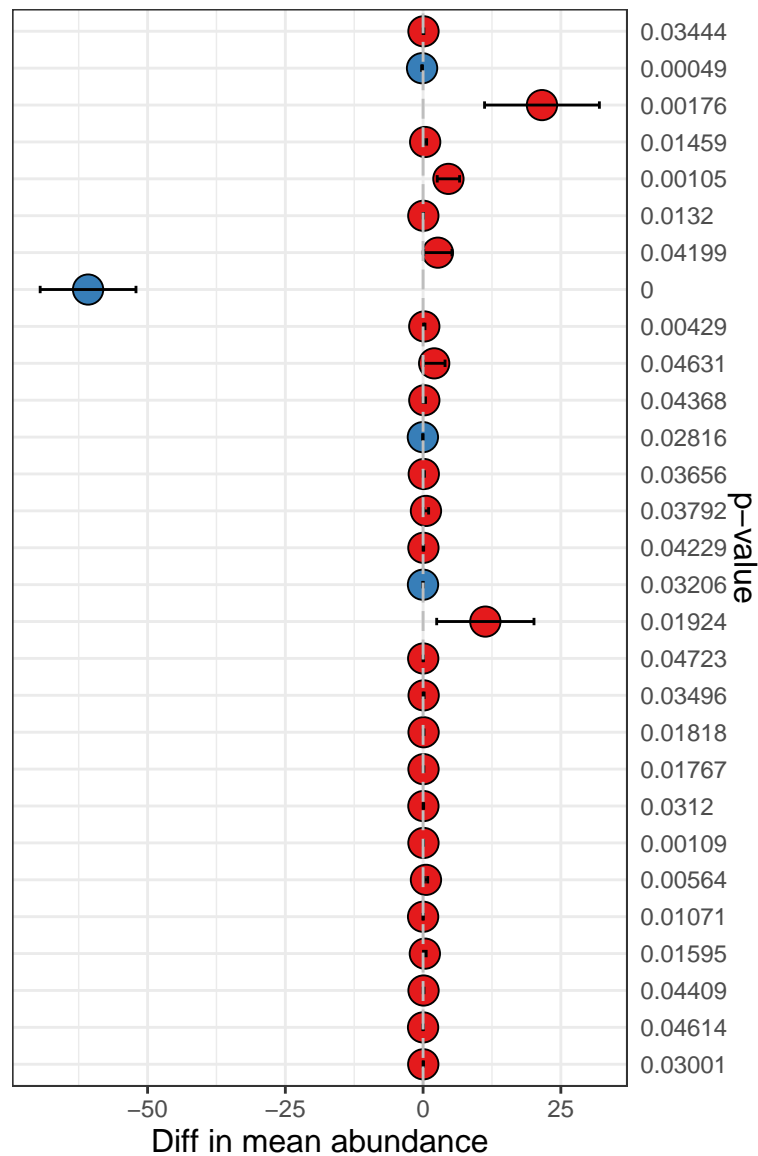

Supplement: Supplementary file 1 [file biology-12-00212-s001.zip › 16s rDNA SEQ/2.Taxa_diff/Genus/CF_vs_HF.t-test.extended_error_bar.pdf]

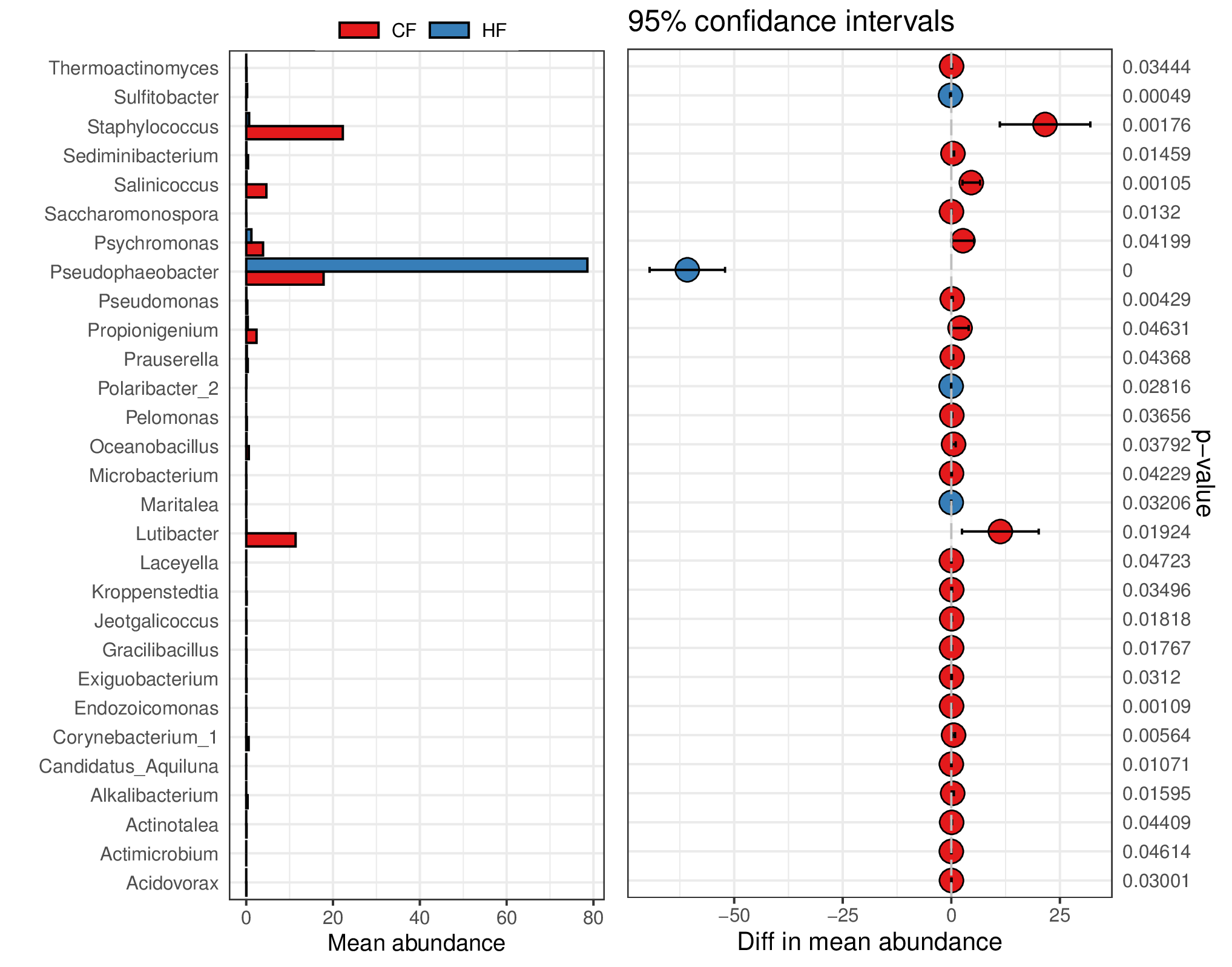

Supplement: Supplementary file 1 [file biology-12-00212-s001.zip › 16s rDNA SEQ/2.Taxa_diff/Genus/CF_vs_HF.t-test.extended_error_bar.png]

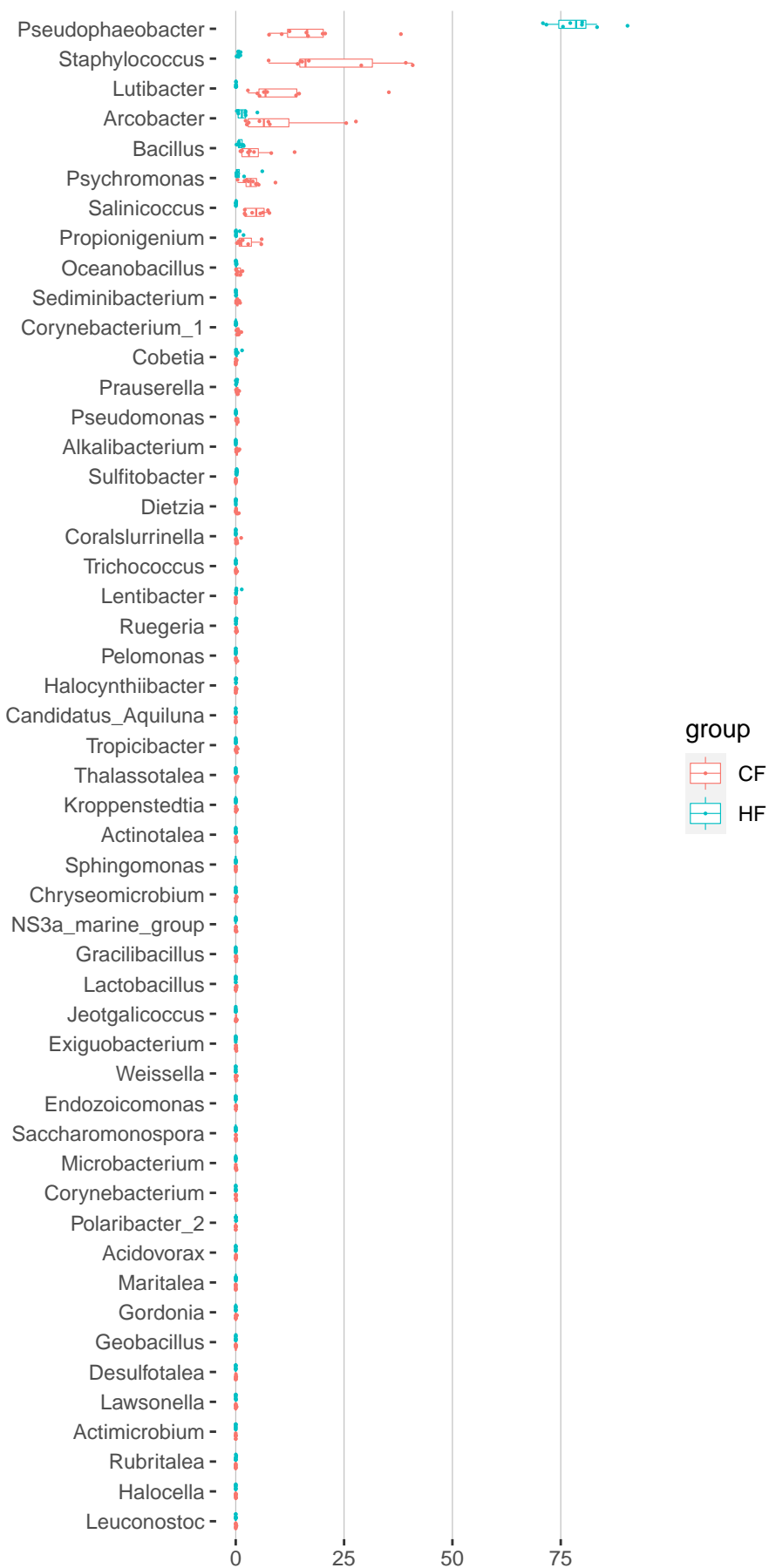

Supplement: Supplementary file 1 [file biology-12-00212-s001.zip › 16s rDNA SEQ/2.Taxa_diff/Genus/CF_vs_HF.wilcox.boxplot.pdf]

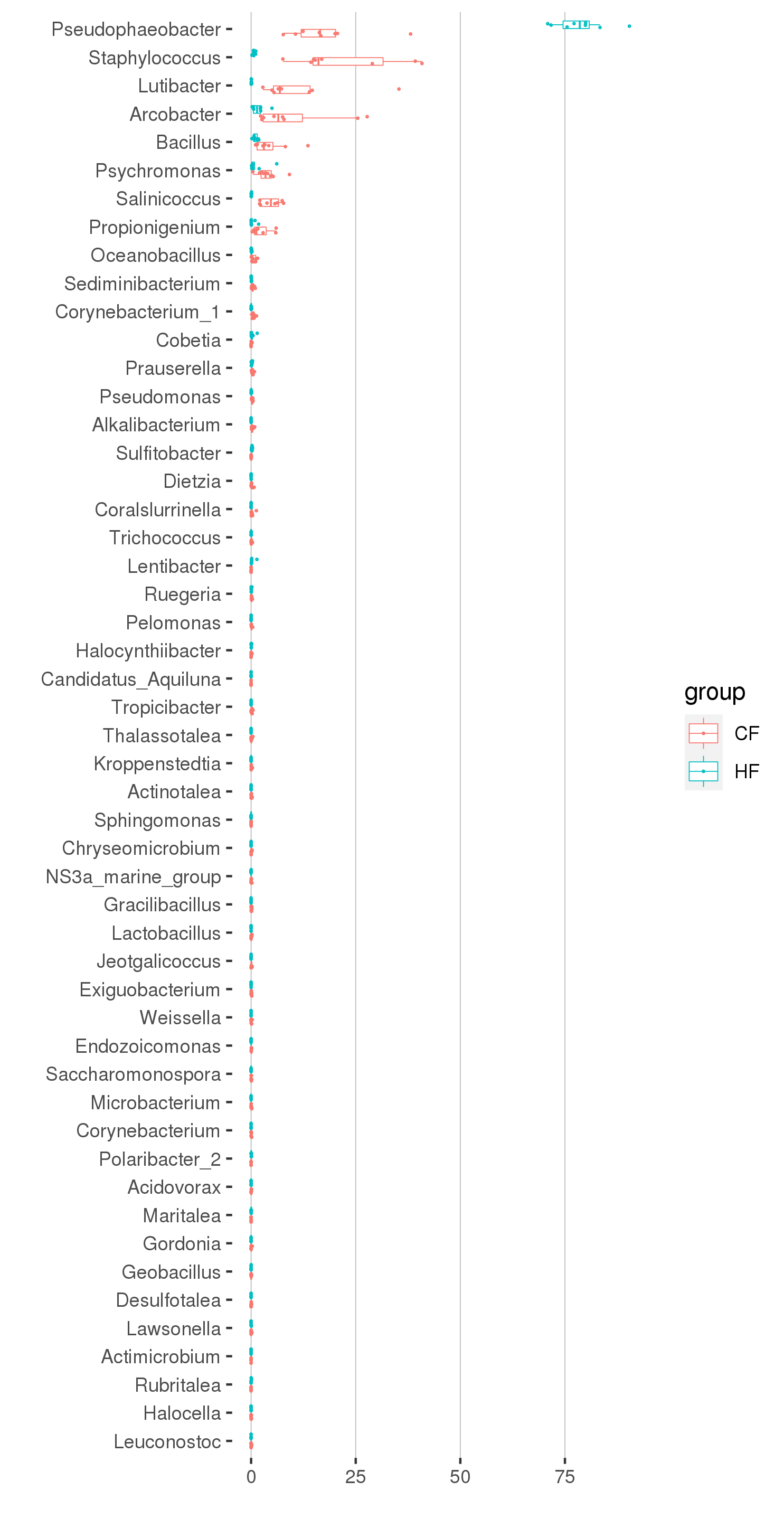

Supplement: Supplementary file 1 [file biology-12-00212-s001.zip › 16s rDNA SEQ/2.Taxa_diff/Genus/CF_vs_HF.wilcox.boxplot.png]

Actimicrobium -

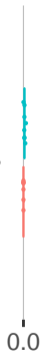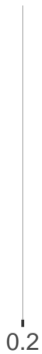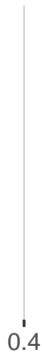

group

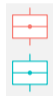

CM

CF

Supplement: Supplementary file 1 [file biology-12-00212-s001.zip › 16s rDNA SEQ/2.Taxa_diff/Genus/CM_vs_CF.wilcox.boxplot.pdf]

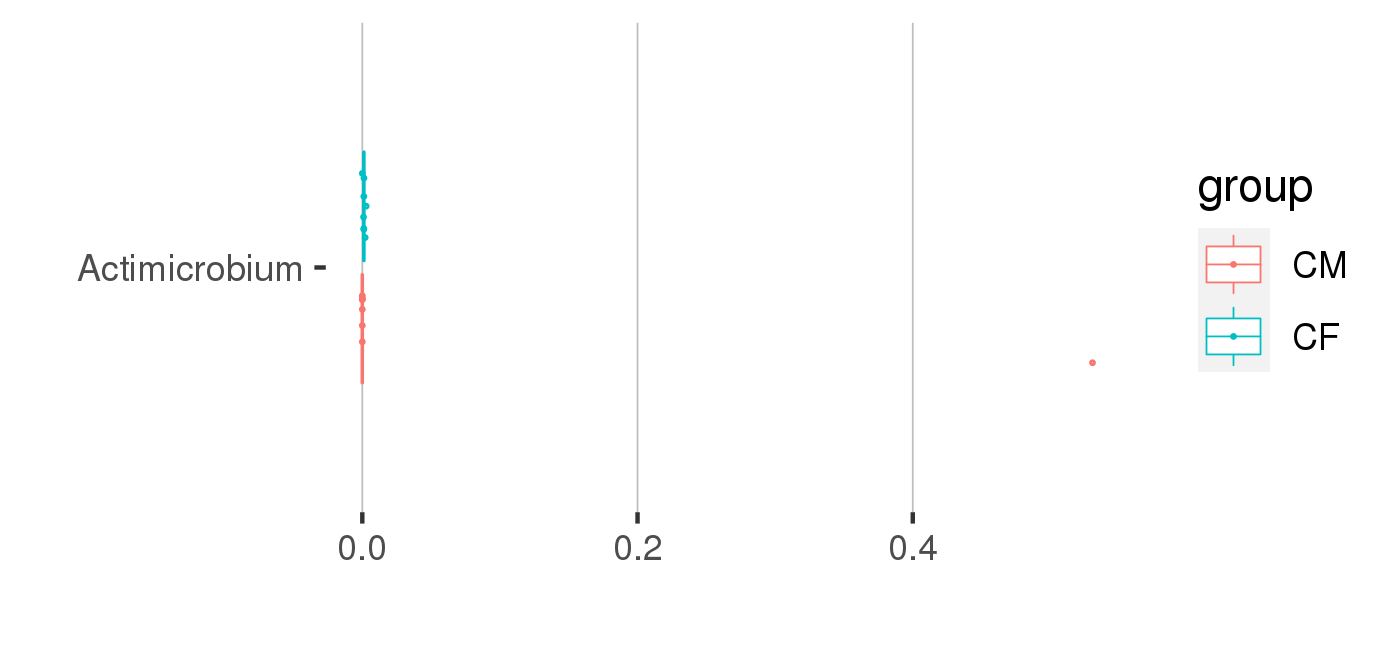

Supplement: Supplementary file 1 [file biology-12-00212-s001.zip › 16s rDNA SEQ/2.Taxa_diff/Genus/CM_vs_CF.wilcox.boxplot.png]

CM HM

95% confidence intervals

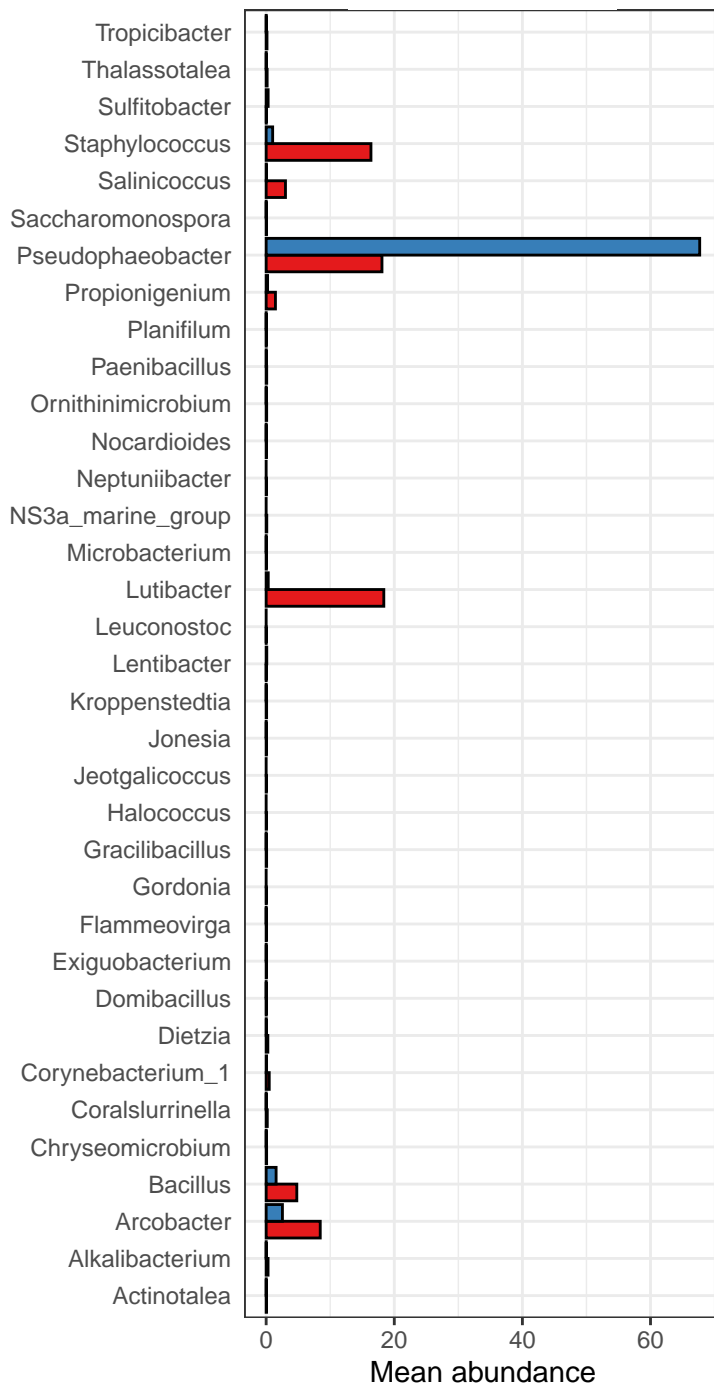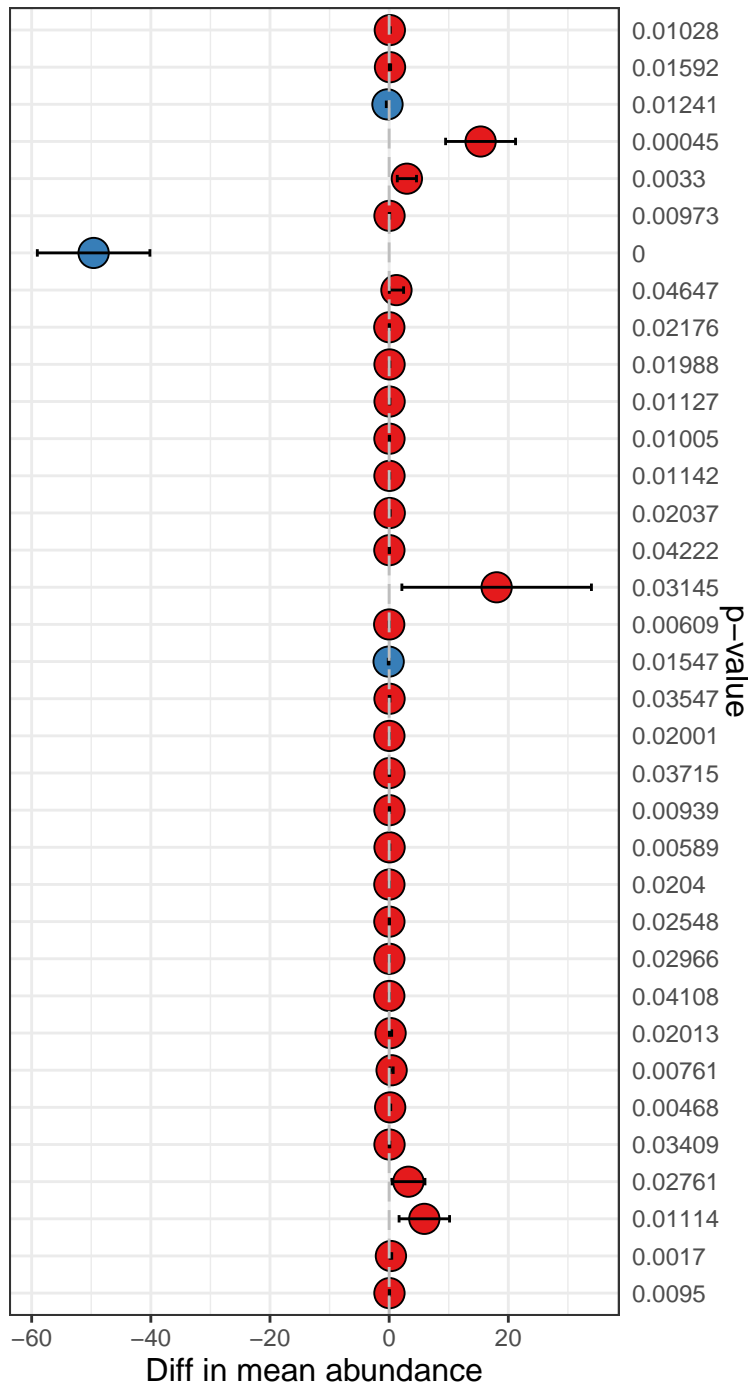

Supplement: Supplementary file 1 [file biology-12-00212-s001.zip › 16s rDNA SEQ/2.Taxa_diff/Genus/CM_vs_HM.t-test.extended_error_bar.pdf]

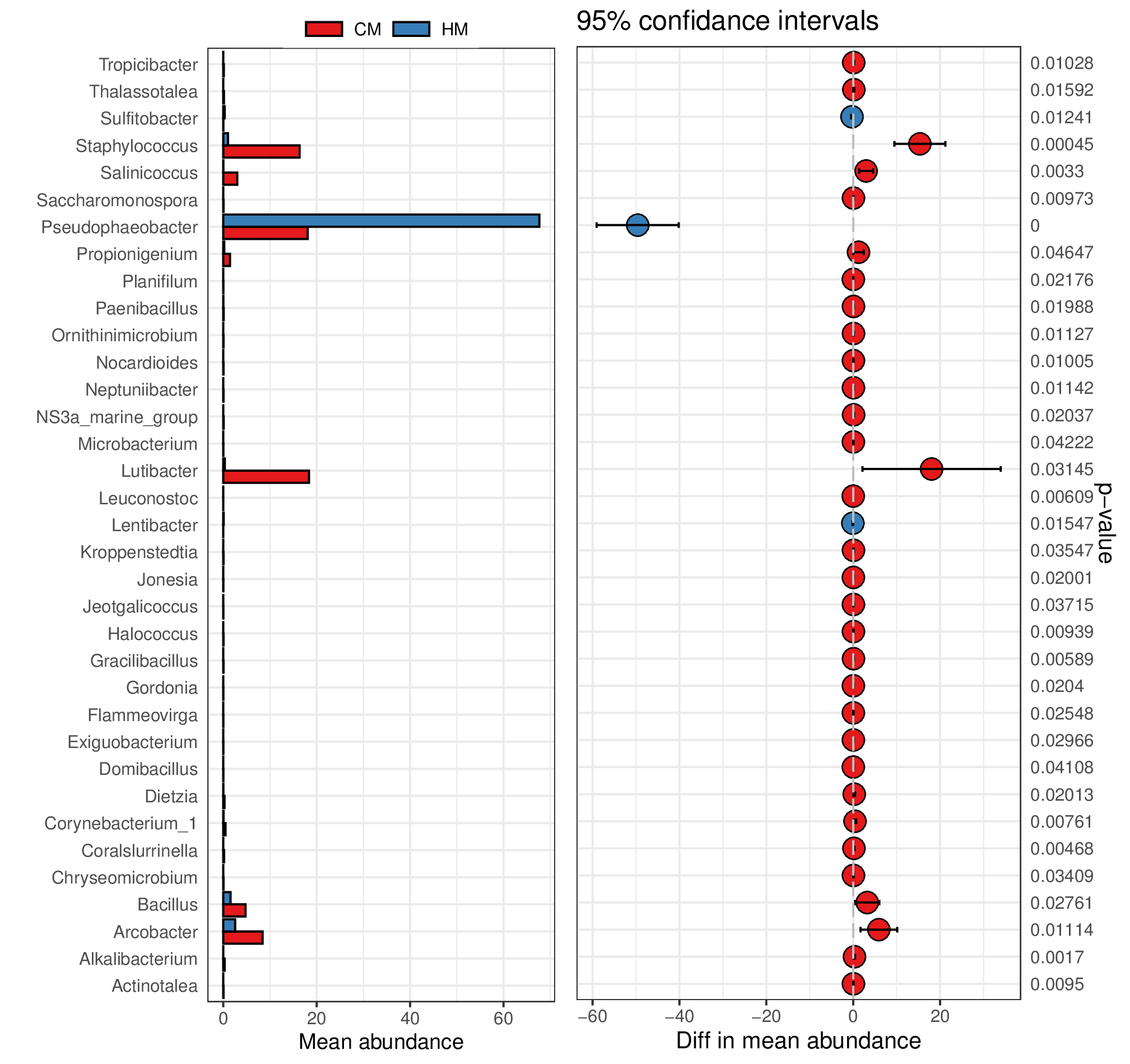

Supplement: Supplementary file 1 [file biology-12-00212-s001.zip › 16s rDNA SEQ/2.Taxa_diff/Genus/CM_vs_HM.t-test.extended_error_bar.png]

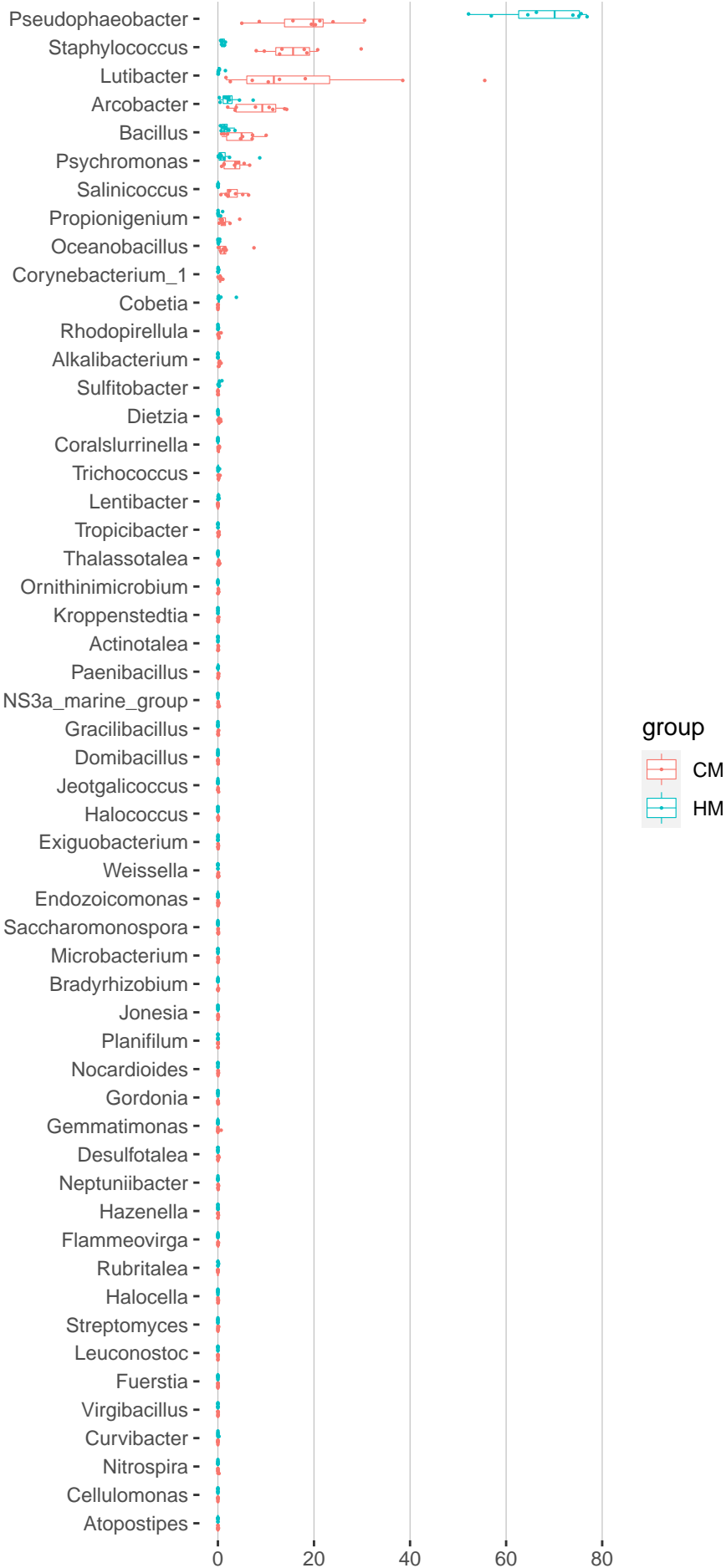

Supplement: Supplementary file 1 [file biology-12-00212-s001.zip › 16s rDNA SEQ/2.Taxa_diff/Genus/CM_vs_HM.wilcox.boxplot.pdf]

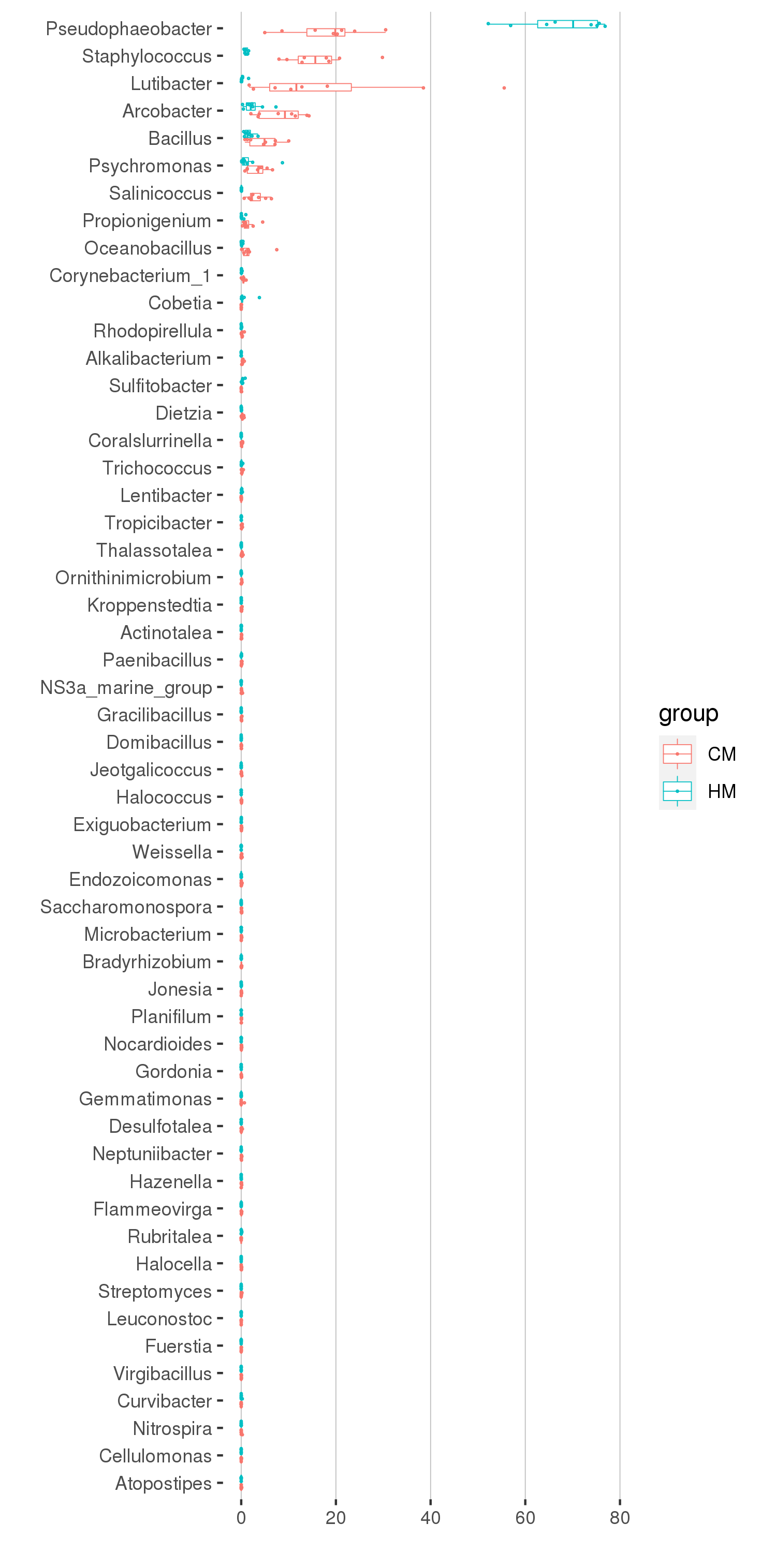

Supplement: Supplementary file 1 [file biology-12-00212-s001.zip › 16s rDNA SEQ/2.Taxa_diff/Genus/CM_vs_HM.wilcox.boxplot.png]

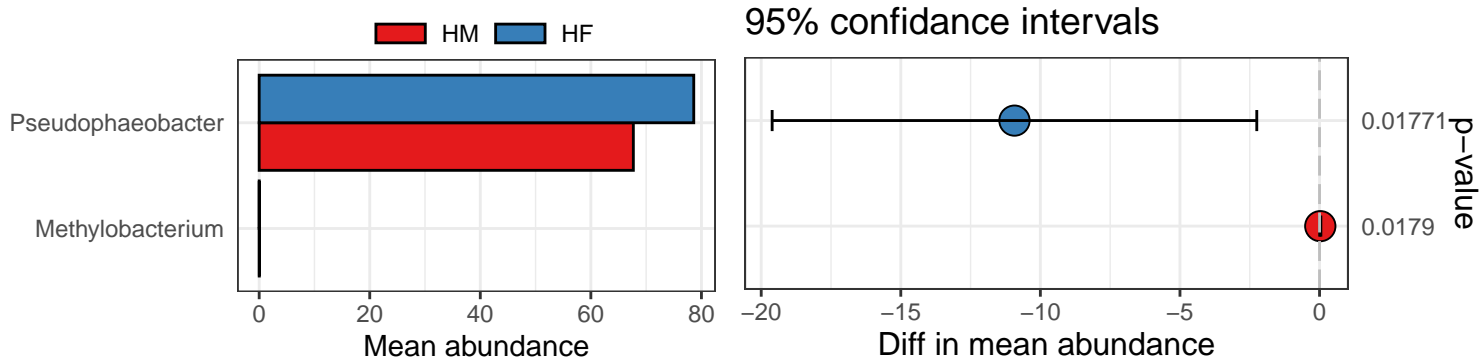

Supplement: Supplementary file 1 [file biology-12-00212-s001.zip › 16s rDNA SEQ/2.Taxa_diff/Genus/HM_vs_HF.t-test.extended_error_bar.pdf]

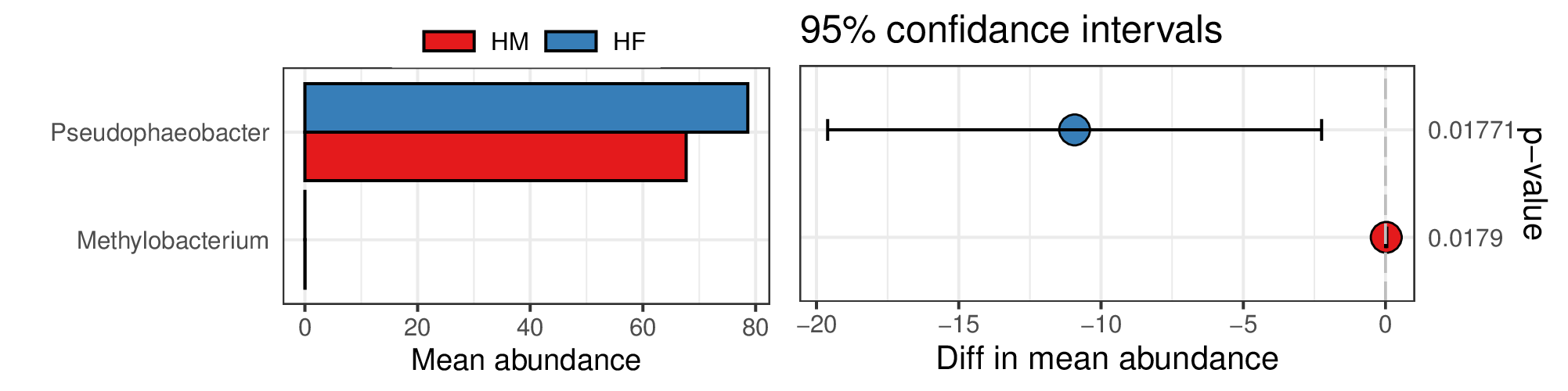

Supplement: Supplementary file 1 [file biology-12-00212-s001.zip › 16s rDNA SEQ/2.Taxa_diff/Genus/HM_vs_HF.t-test.extended_error_bar.png]

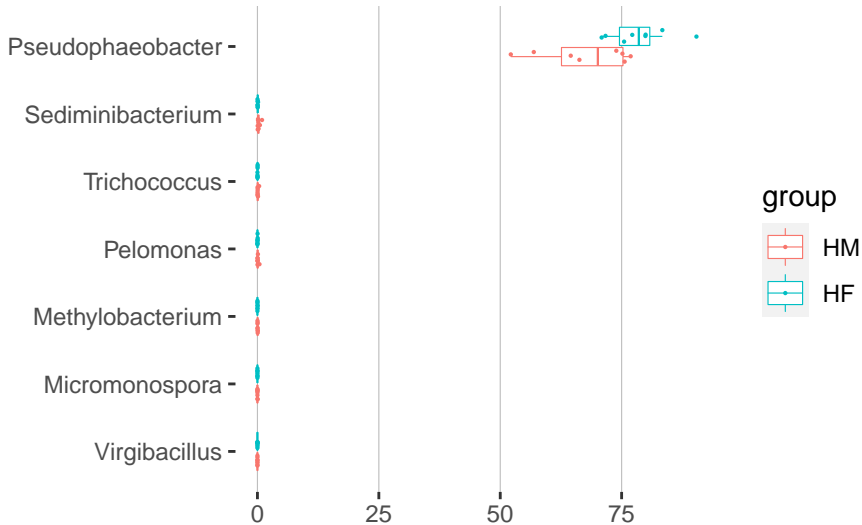

Supplement: Supplementary file 1 [file biology-12-00212-s001.zip › 16s rDNA SEQ/2.Taxa_diff/Genus/HM_vs_HF.wilcox.boxplot.pdf]

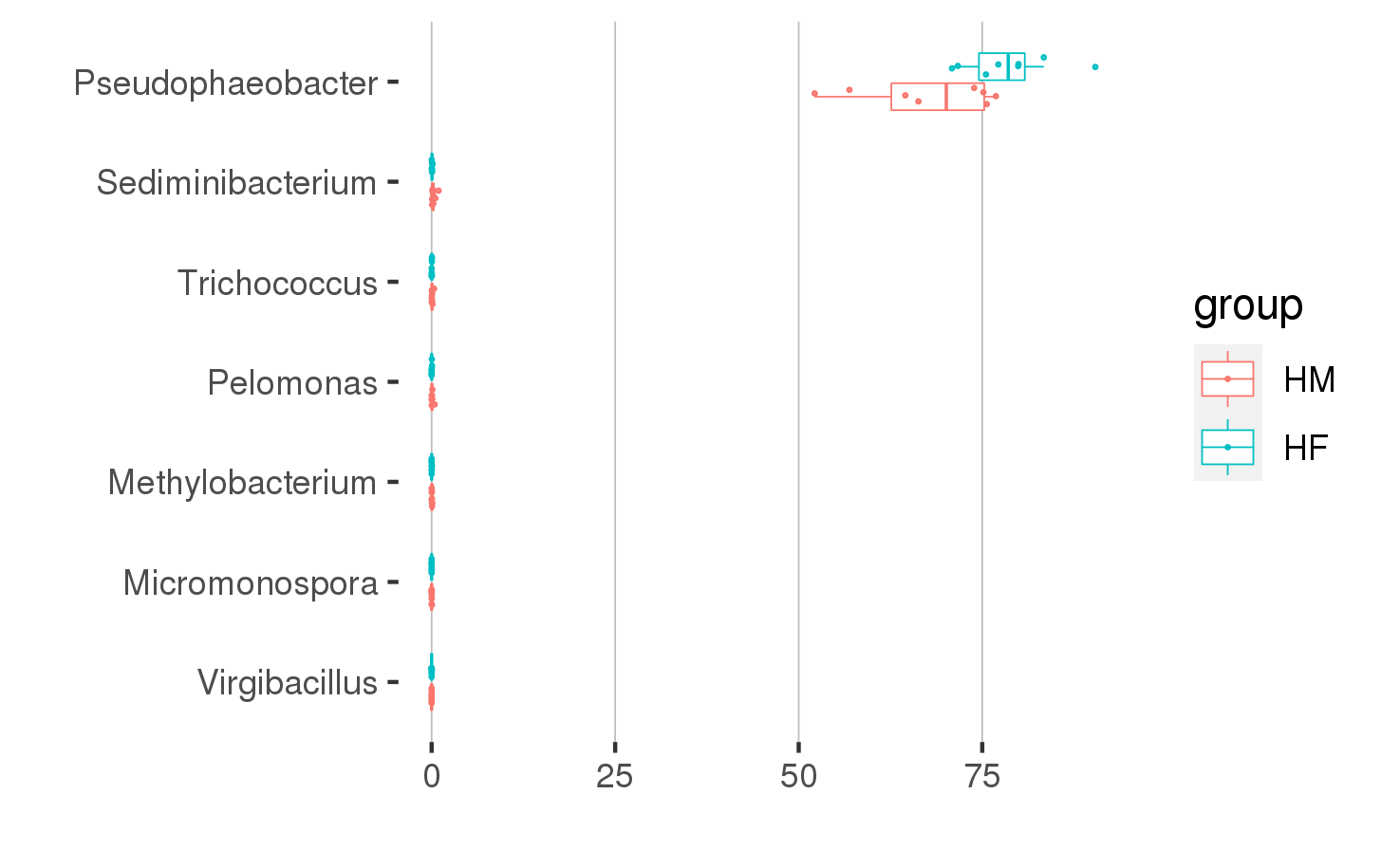

Supplement: Supplementary file 1 [file biology-12-00212-s001.zip › 16s rDNA SEQ/2.Taxa_diff/Genus/HM_vs_HF.wilcox.boxplot.png]

CF HF

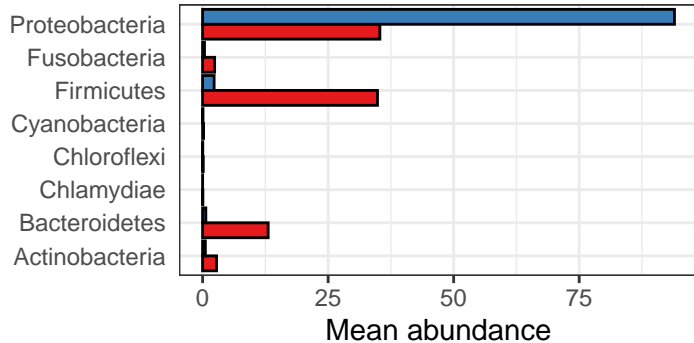

95% confidence intervals

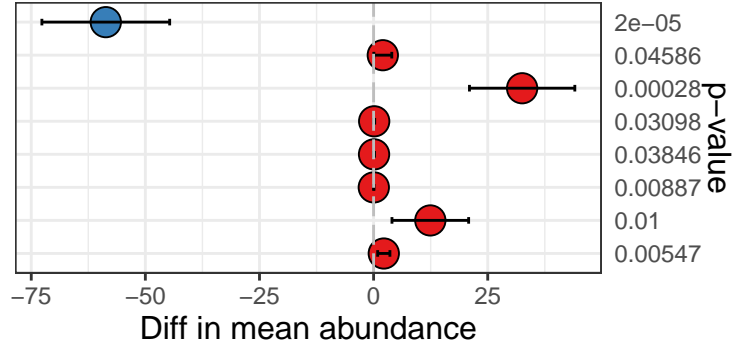

Supplement: Supplementary file 1 [file biology-12-00212-s001.zip › 16s rDNA SEQ/2.Taxa_diff/Phylum/CF_vs_HF.t-test.extended_error_bar.pdf]

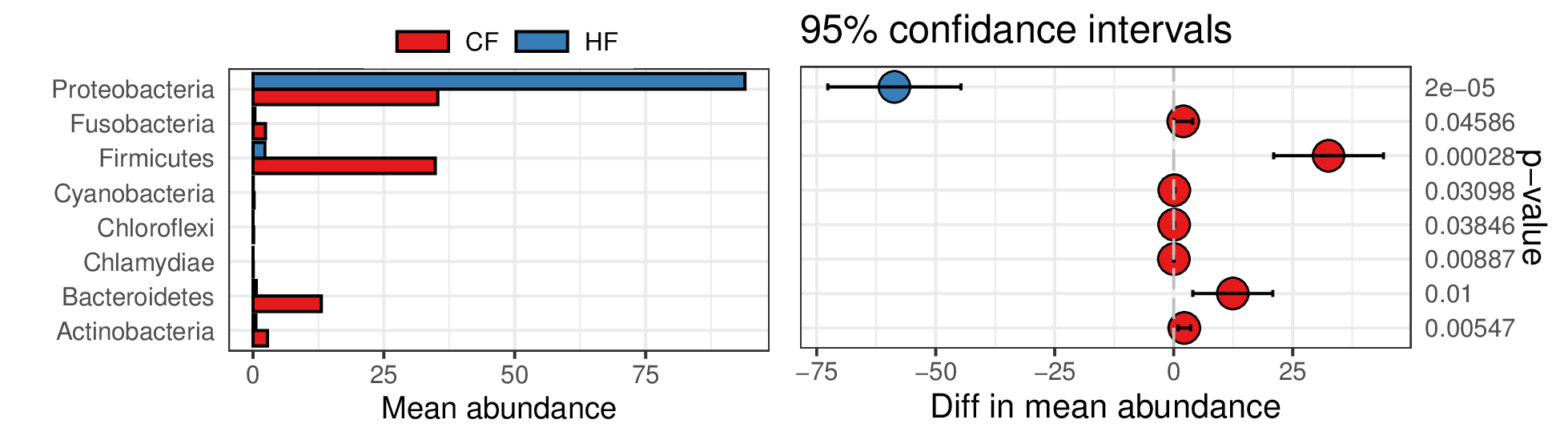

Supplement: Supplementary file 1 [file biology-12-00212-s001.zip › 16s rDNA SEQ/2.Taxa_diff/Phylum/CF_vs_HF.t-test.extended_error_bar.png]

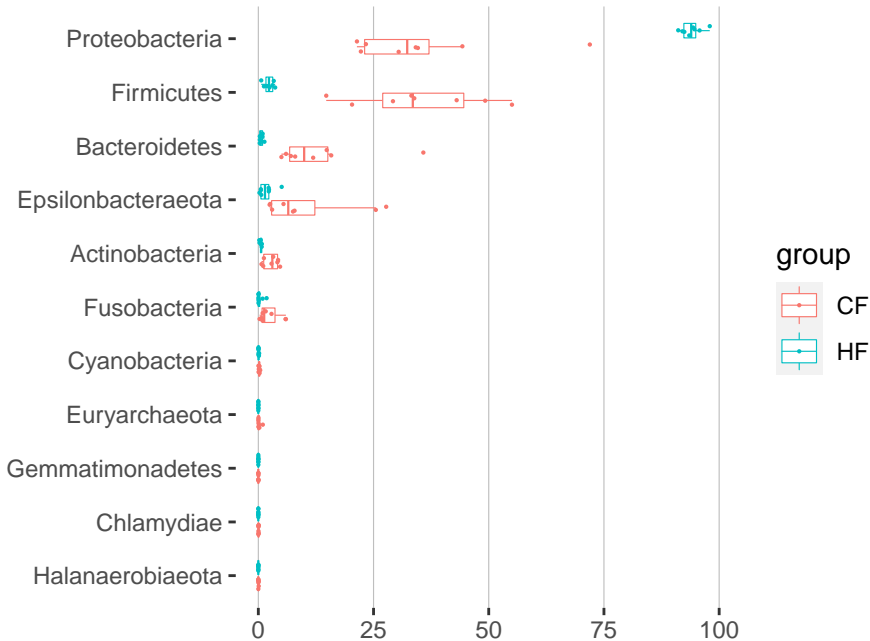

Supplement: Supplementary file 1 [file biology-12-00212-s001.zip › 16s rDNA SEQ/2.Taxa_diff/Phylum/CF_vs_HF.wilcox.boxplot.pdf]

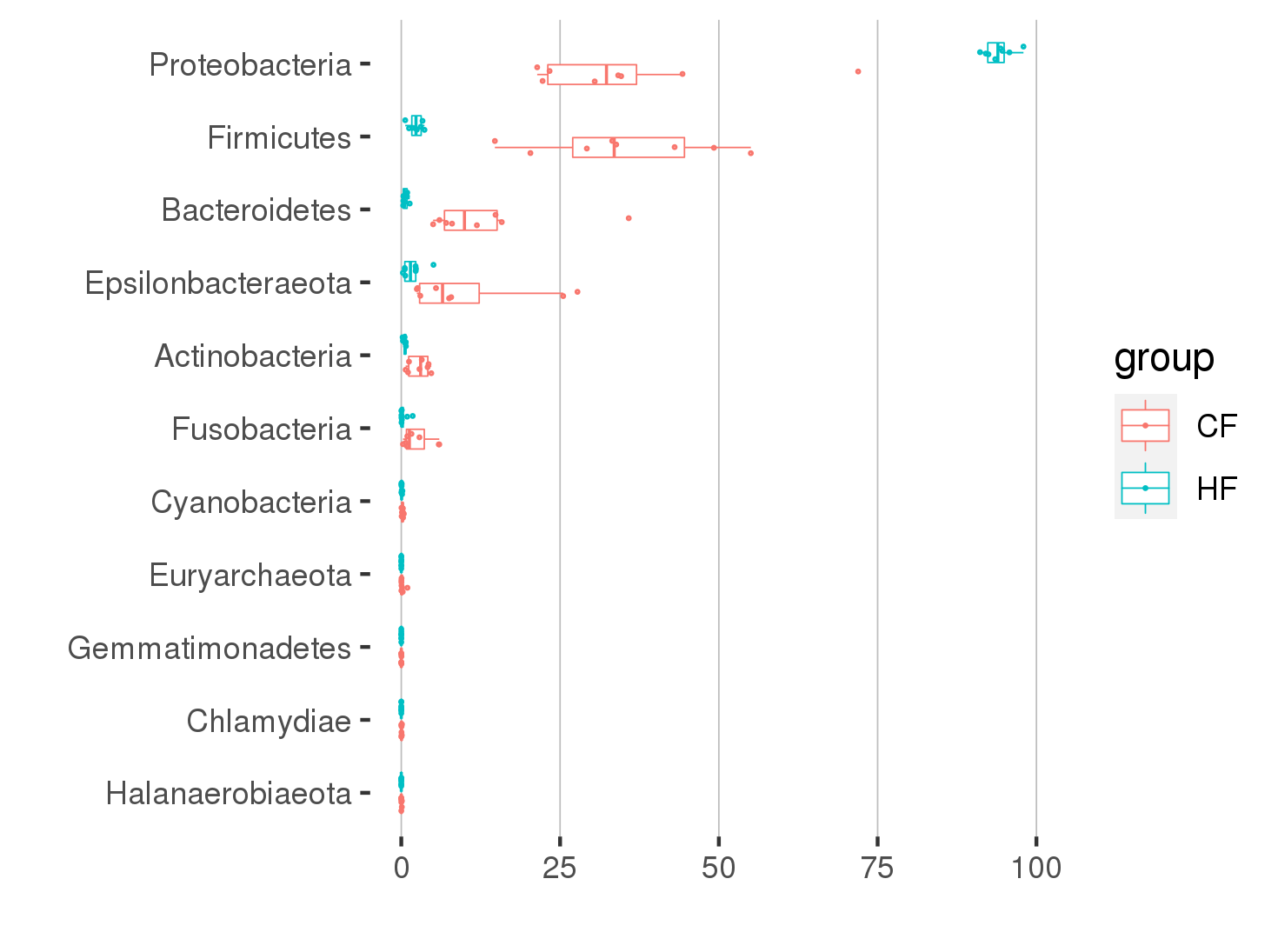

Supplement: Supplementary file 1 [file biology-12-00212-s001.zip › 16s rDNA SEQ/2.Taxa_diff/Phylum/CF_vs_HF.wilcox.boxplot.png]

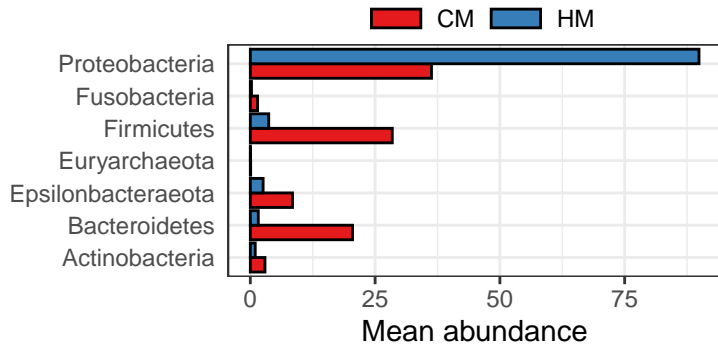

## 95% confidence intervals

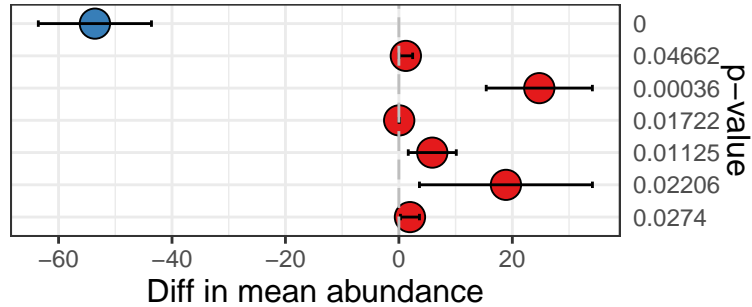

Supplement: Supplementary file 1 [file biology-12-00212-s001.zip › 16s rDNA SEQ/2.Taxa_diff/Phylum/CM_vs_HM.t-test.extended_error_bar.pdf]

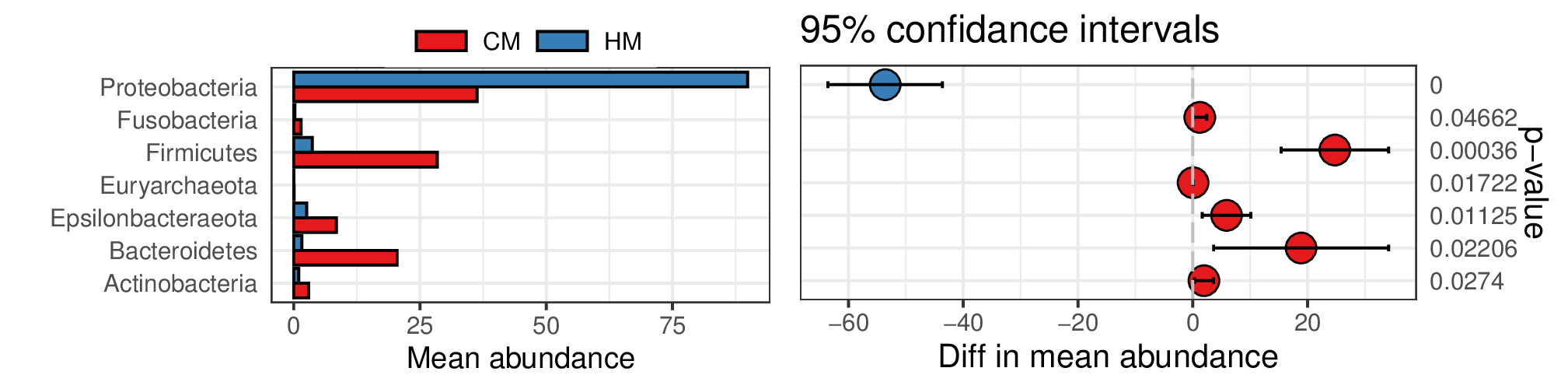

Supplement: Supplementary file 1 [file biology-12-00212-s001.zip › 16s rDNA SEQ/2.Taxa_diff/Phylum/CM_vs_HM.t-test.extended_error_bar.png]

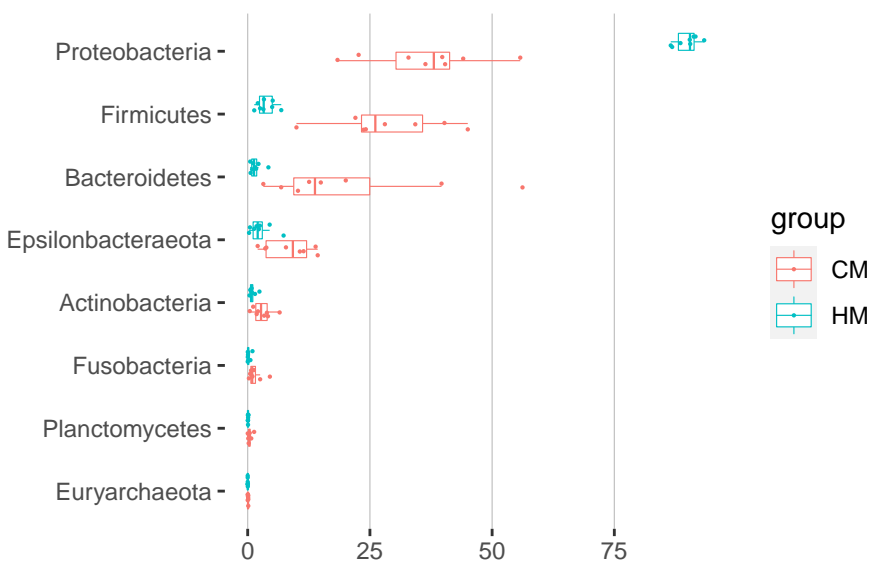

Supplement: Supplementary file 1 [file biology-12-00212-s001.zip › 16s rDNA SEQ/2.Taxa_diff/Phylum/CM_vs_HM.wilcox.boxplot.pdf]

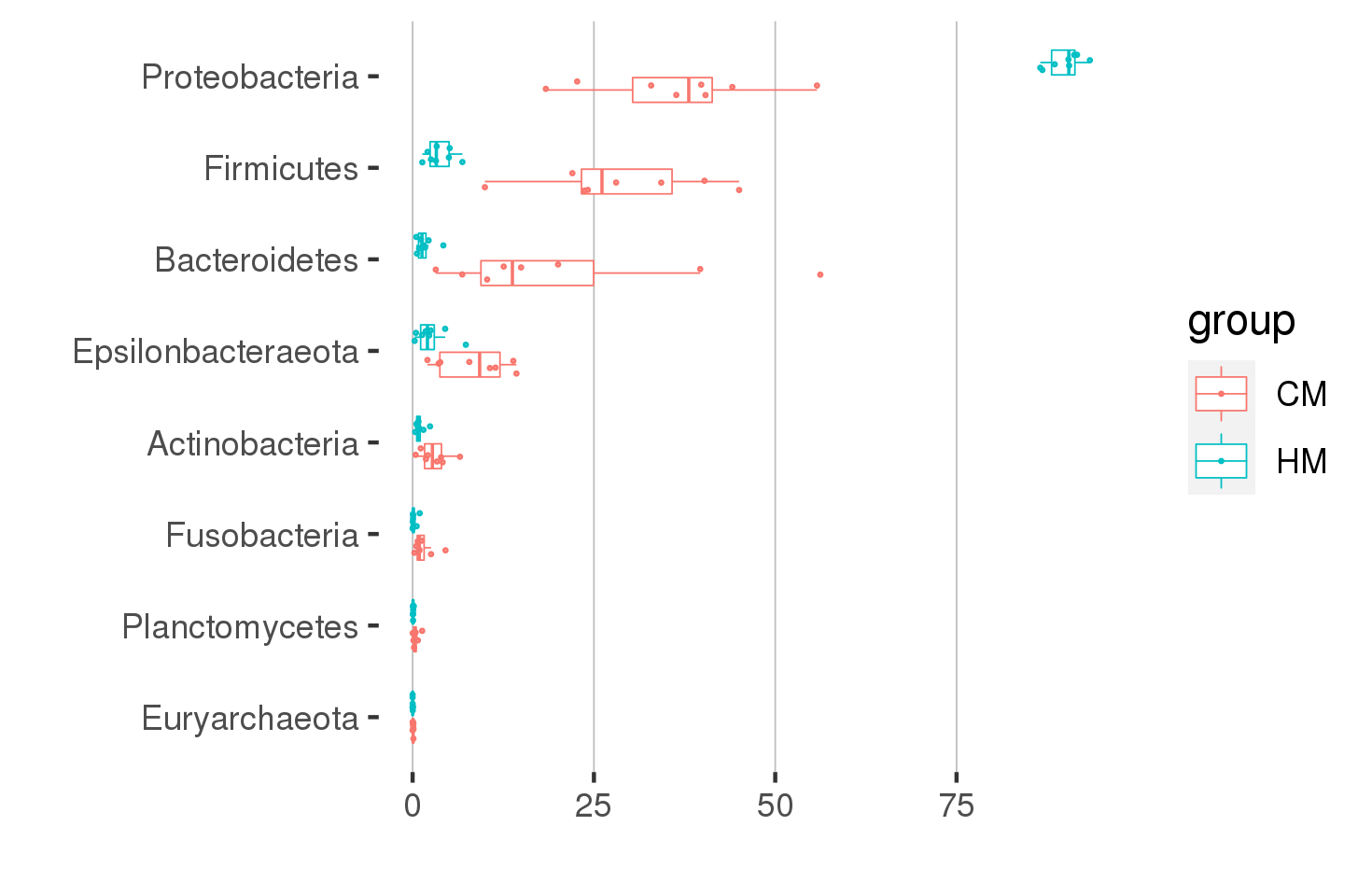

Supplement: Supplementary file 1 [file biology-12-00212-s001.zip › 16s rDNA SEQ/2.Taxa_diff/Phylum/CM_vs_HM.wilcox.boxplot.png]

HM HF

Proteobacteria

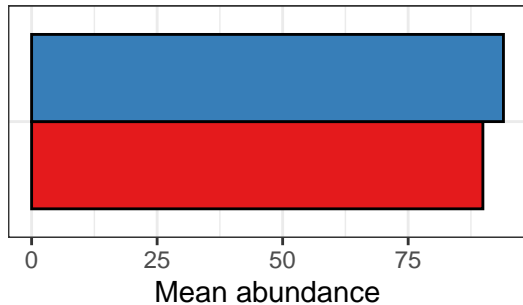

95% confidence intervals

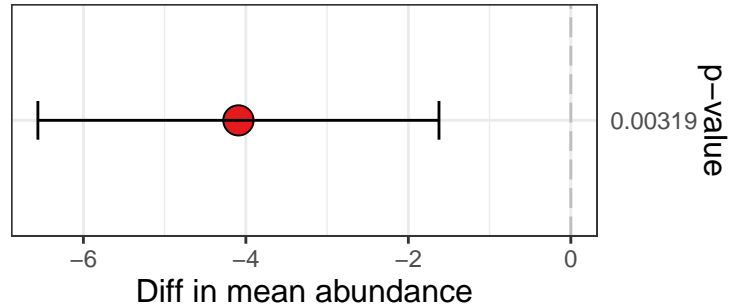

Supplement: Supplementary file 1 [file biology-12-00212-s001.zip › 16s rDNA SEQ/2.Taxa_diff/Phylum/HM_vs_HF.t-test.extended_error_bar.pdf]

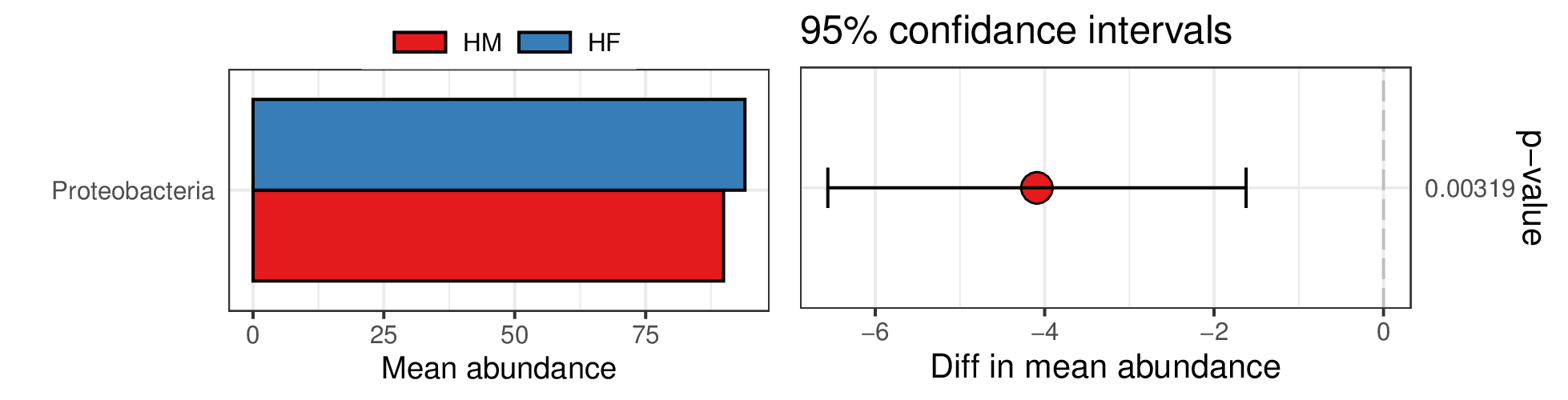

Supplement: Supplementary file 1 [file biology-12-00212-s001.zip › 16s rDNA SEQ/2.Taxa_diff/Phylum/HM_vs_HF.t-test.extended_error_bar.png]

Proteobacteria -

Bacteroidetes -

Cyanobacteria -

0

25

50

75

100

group

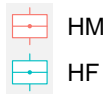

Supplement: Supplementary file 1 [file biology-12-00212-s001.zip › 16s rDNA SEQ/2.Taxa_diff/Phylum/HM_vs_HF.wilcox.boxplot.pdf]

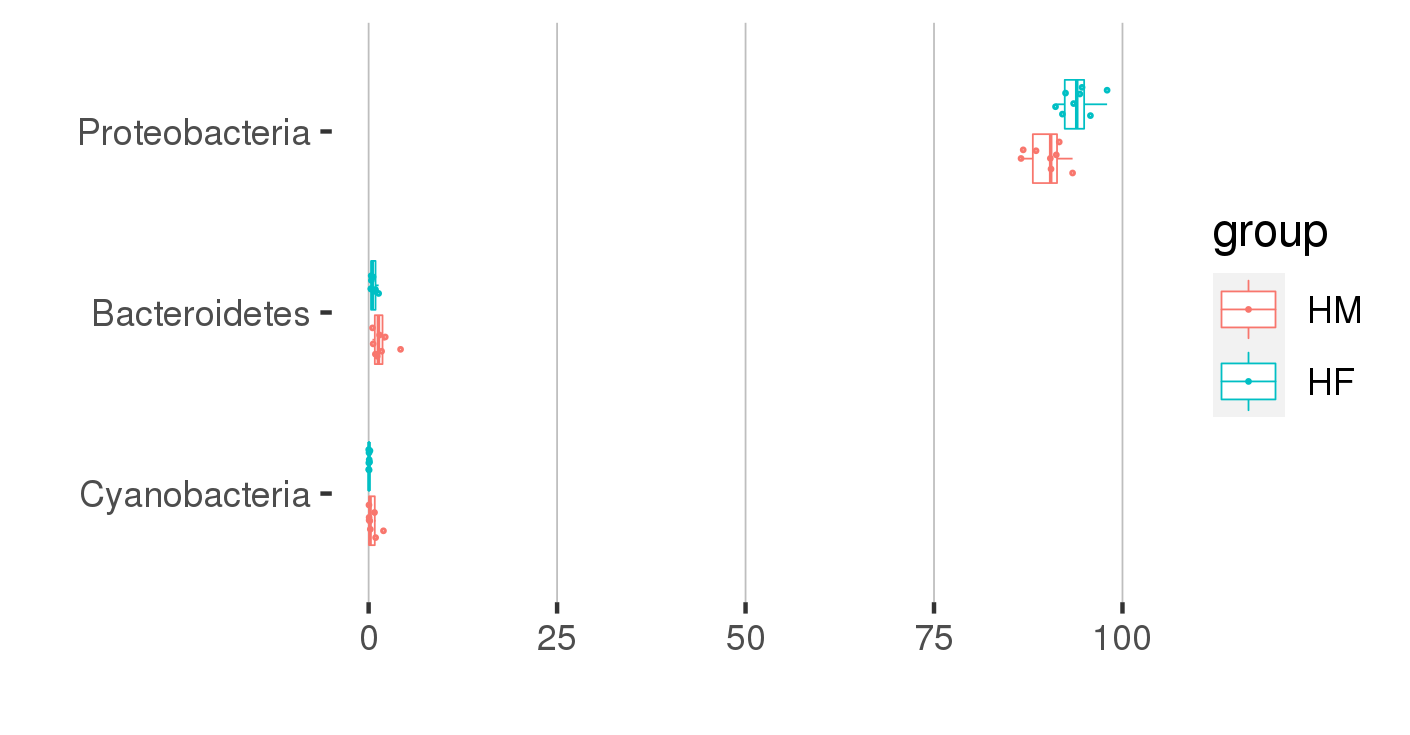

Supplement: Supplementary file 1 [file biology-12-00212-s001.zip › 16s rDNA SEQ/2.Taxa_diff/Phylum/HM_vs_HF.wilcox.boxplot.png]

the boxplot of CF-VS-HF

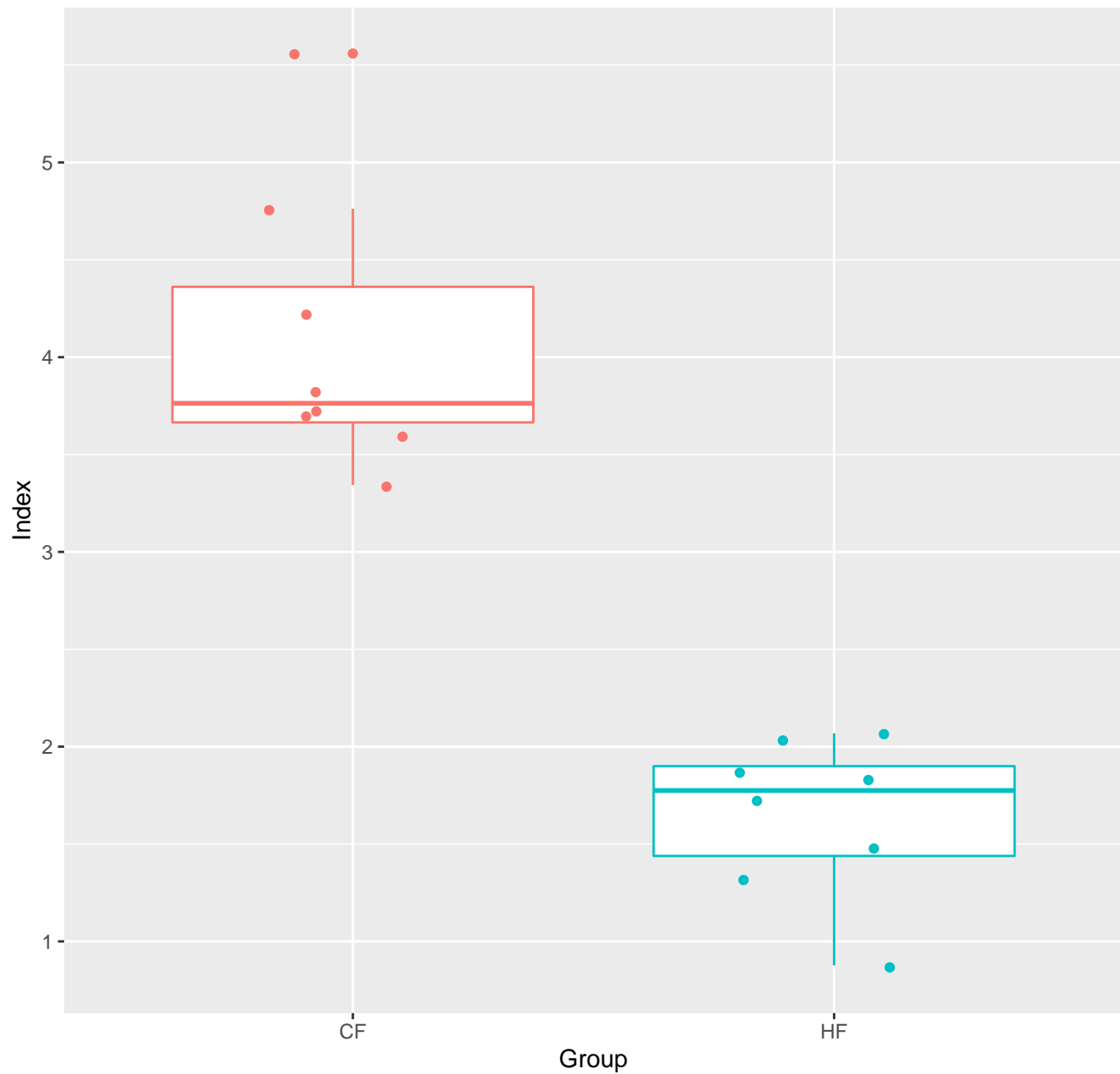

Supplement: Supplementary file 1 [file biology-12-00212-s001.zip › 16s rDNA SEQ/3.Alpha_diversity/2.diff_alpha_diversity/shannon/CF-VS-HF.boxplot.pdf]

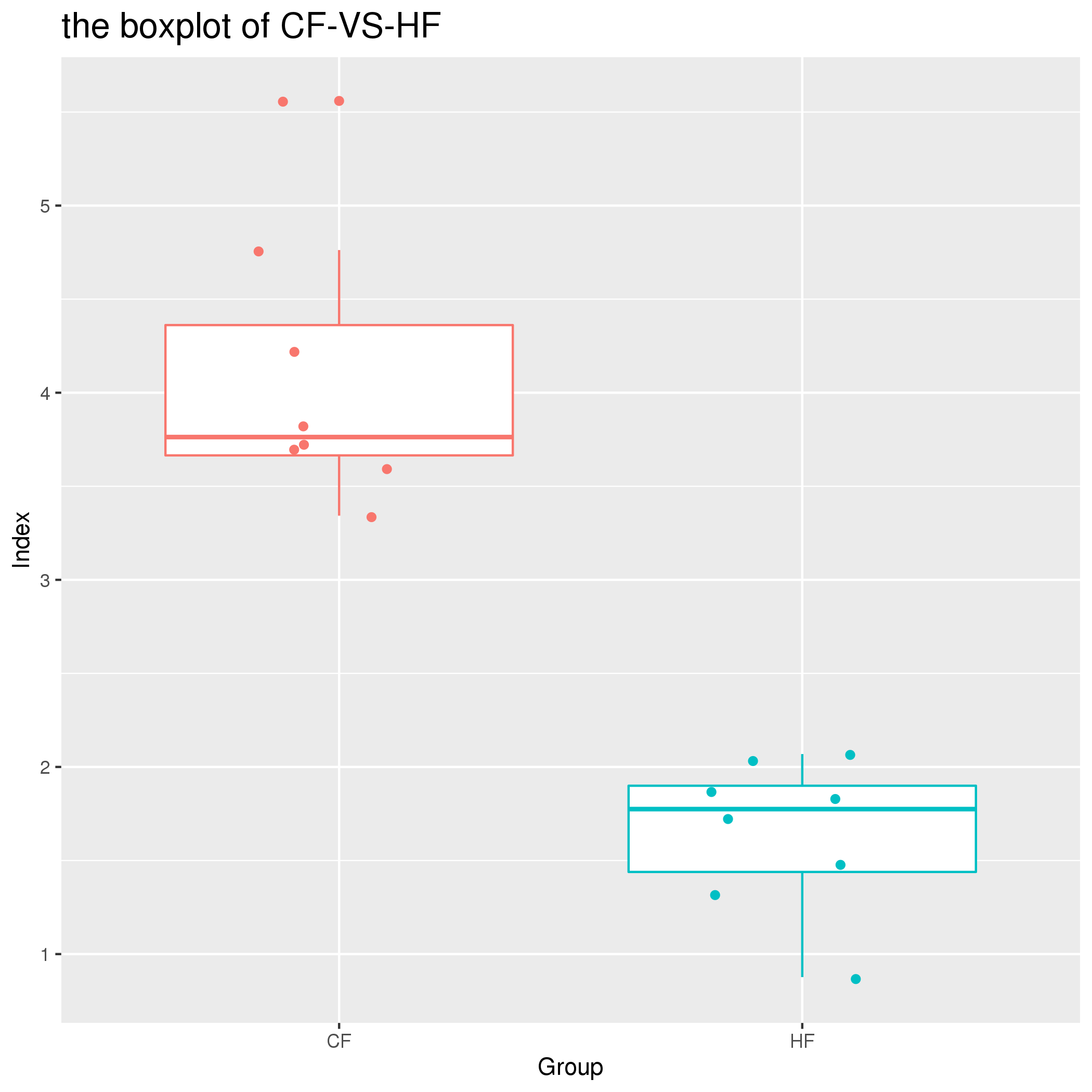

Supplement: Supplementary file 1 [file biology-12-00212-s001.zip › 16s rDNA SEQ/3.Alpha_diversity/2.diff_alpha_diversity/shannon/CF-VS-HF.boxplot.png]

the boxplot of CM-VS-CF

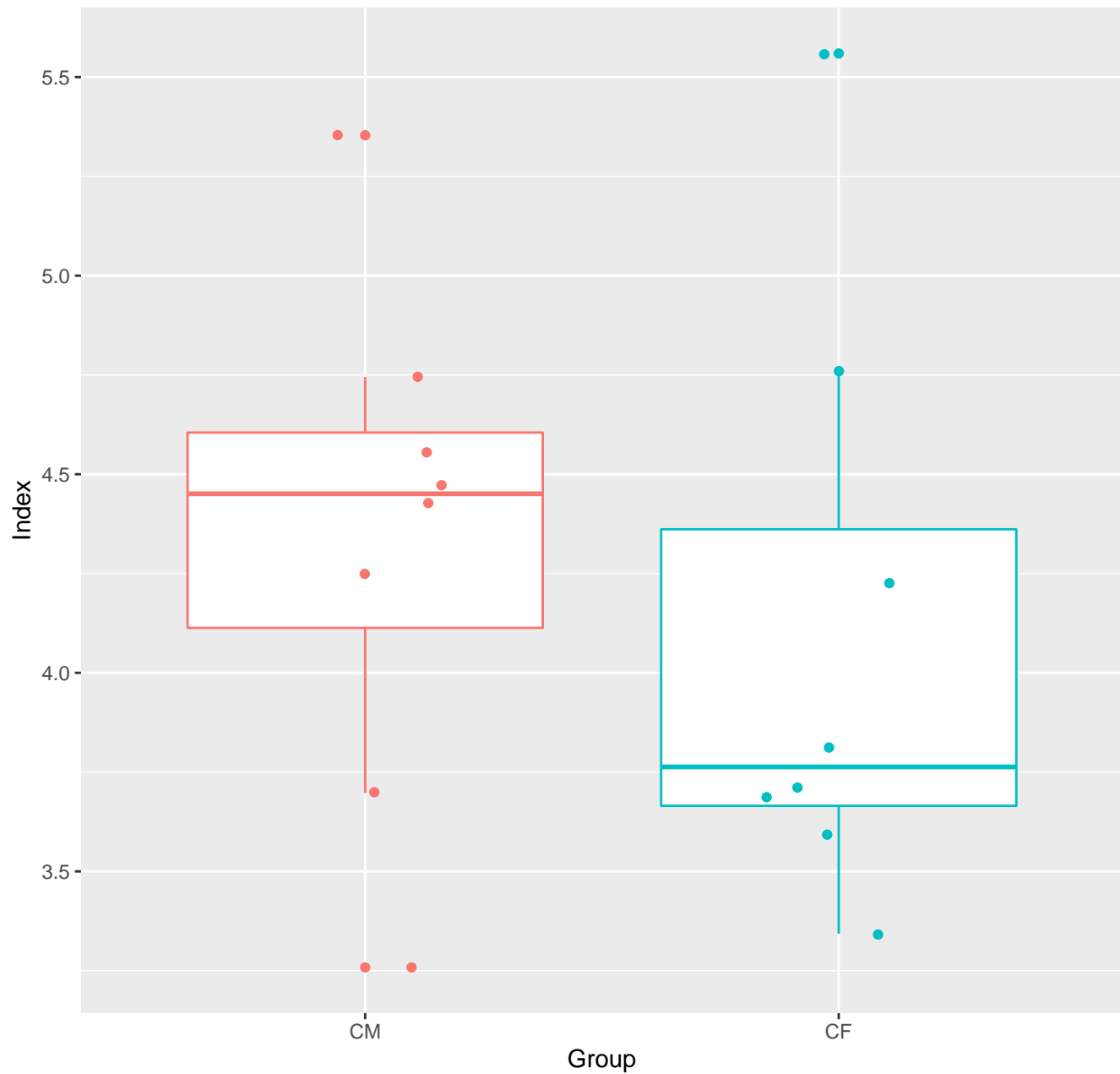

Supplement: Supplementary file 1 [file biology-12-00212-s001.zip › 16s rDNA SEQ/3.Alpha_diversity/2.diff_alpha_diversity/shannon/CM-VS-CF.boxplot.pdf]

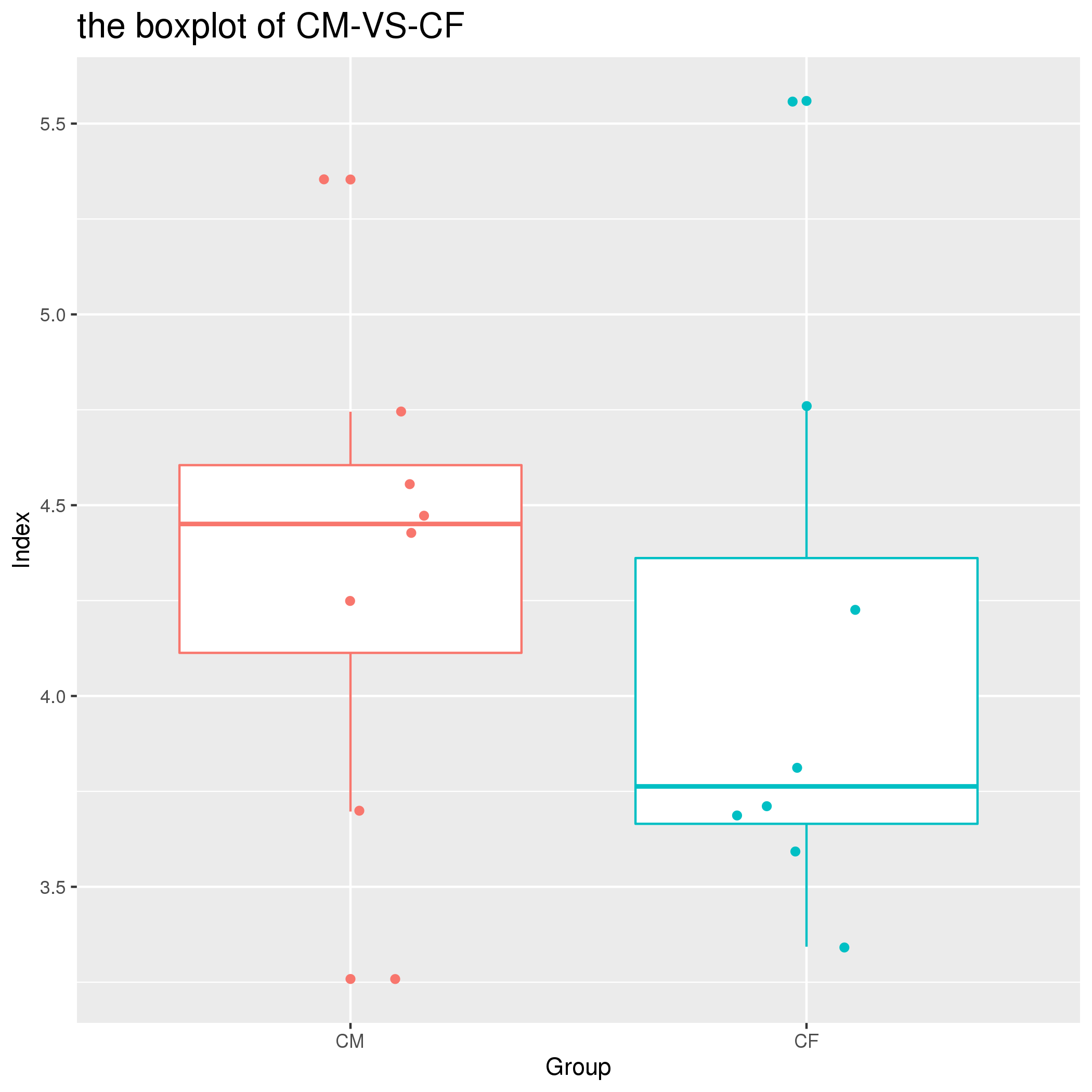

Supplement: Supplementary file 1 [file biology-12-00212-s001.zip › 16s rDNA SEQ/3.Alpha_diversity/2.diff_alpha_diversity/shannon/CM-VS-CF.boxplot.png]

the boxplot of CM-VS-HM

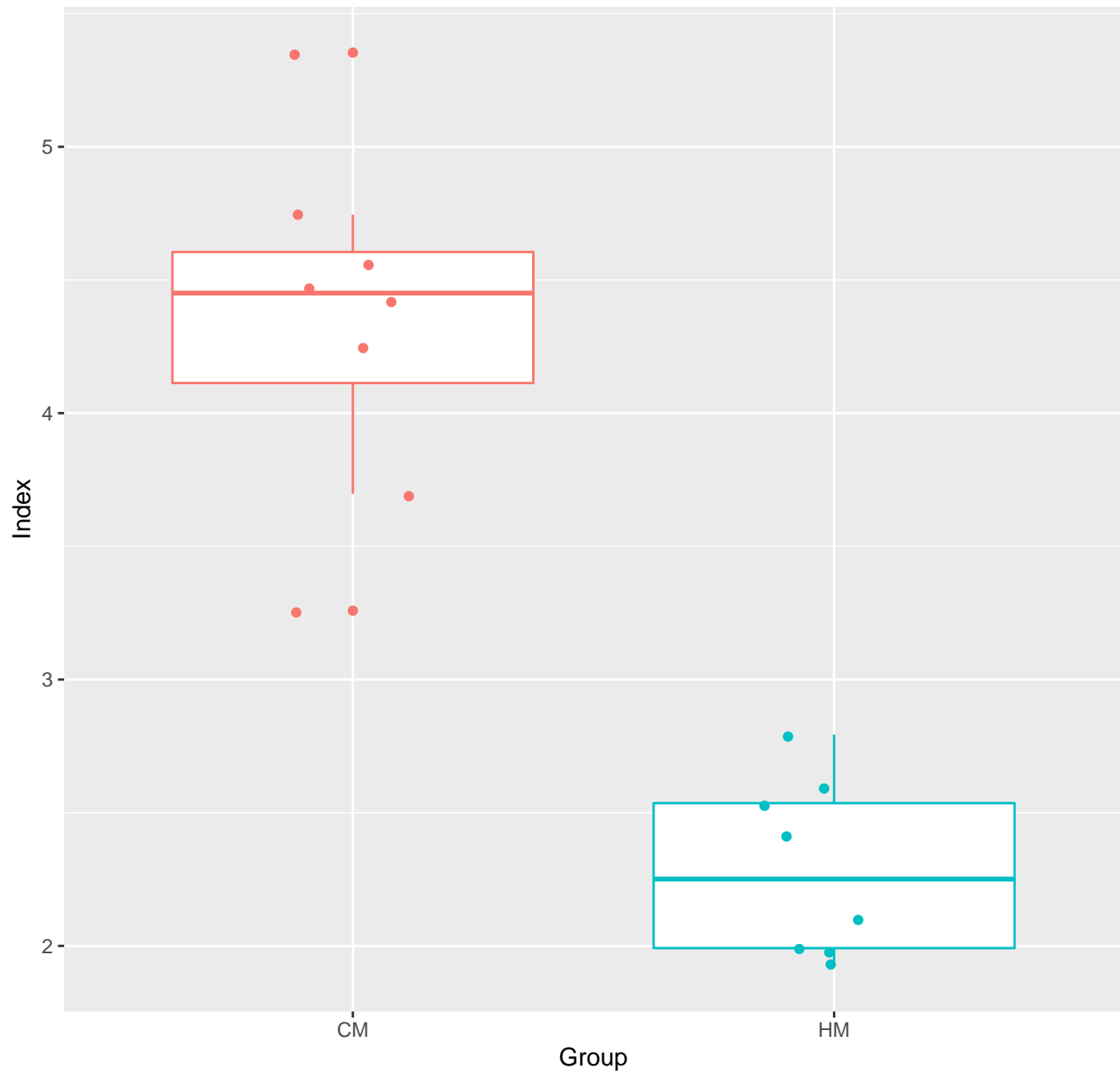

Supplement: Supplementary file 1 [file biology-12-00212-s001.zip › 16s rDNA SEQ/3.Alpha_diversity/2.diff_alpha_diversity/shannon/CM-VS-HM.boxplot.pdf]

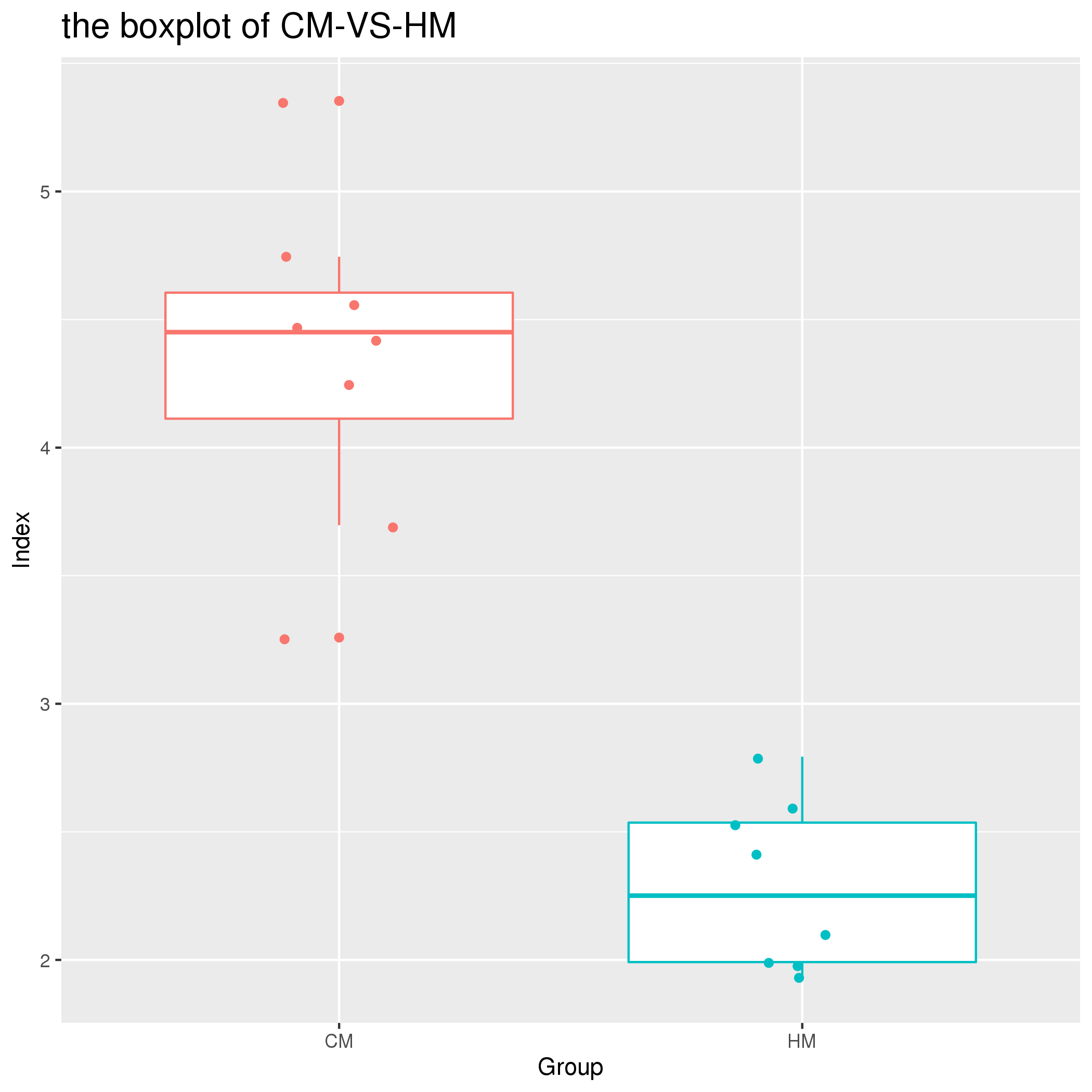

Supplement: Supplementary file 1 [file biology-12-00212-s001.zip › 16s rDNA SEQ/3.Alpha_diversity/2.diff_alpha_diversity/shannon/CM-VS-HM.boxplot.png]

the boxplot of HM-VS-HF

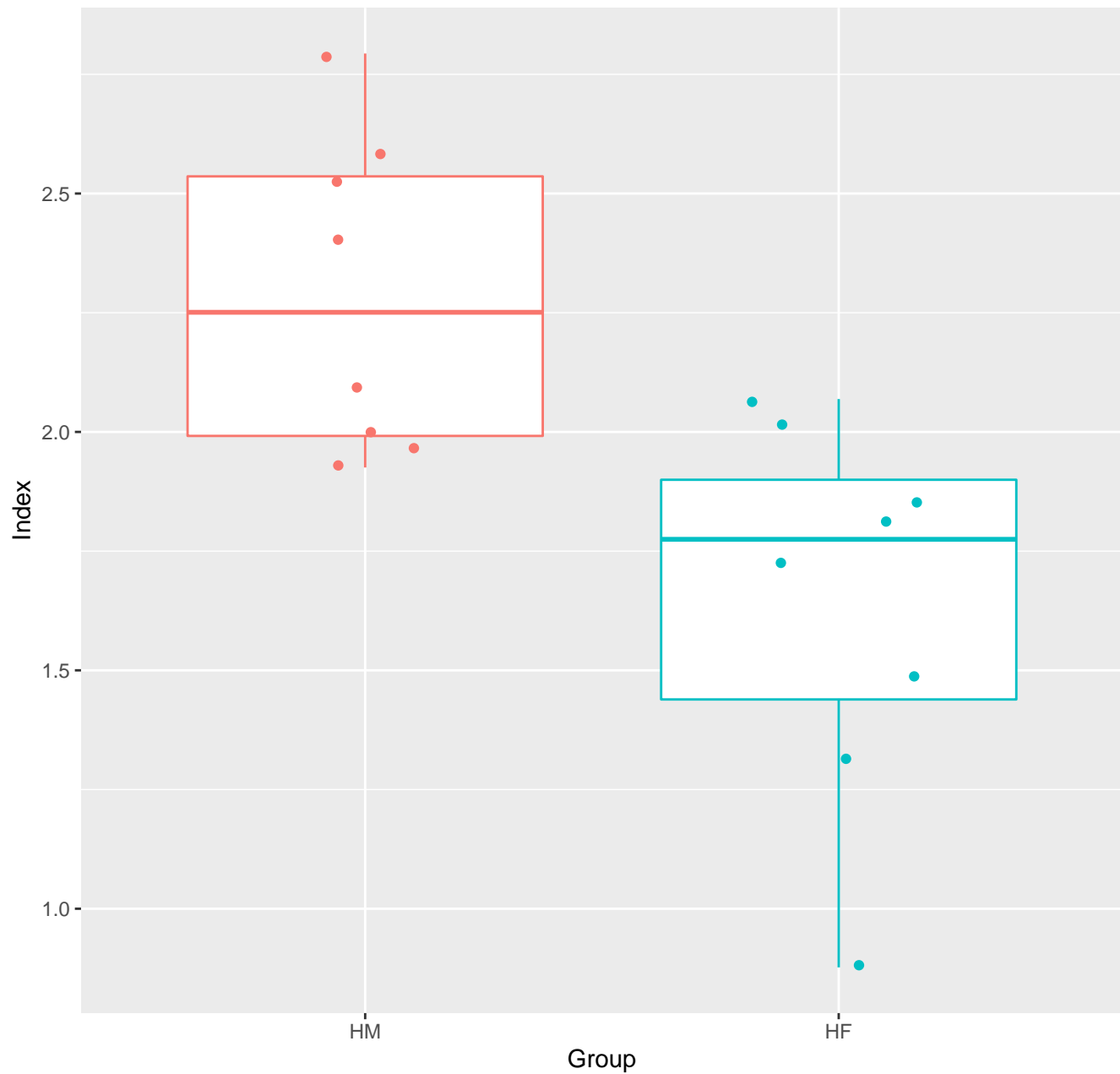

Supplement: Supplementary file 1 [file biology-12-00212-s001.zip › 16s rDNA SEQ/3.Alpha_diversity/2.diff_alpha_diversity/shannon/HM-VS-HF.boxplot.pdf]

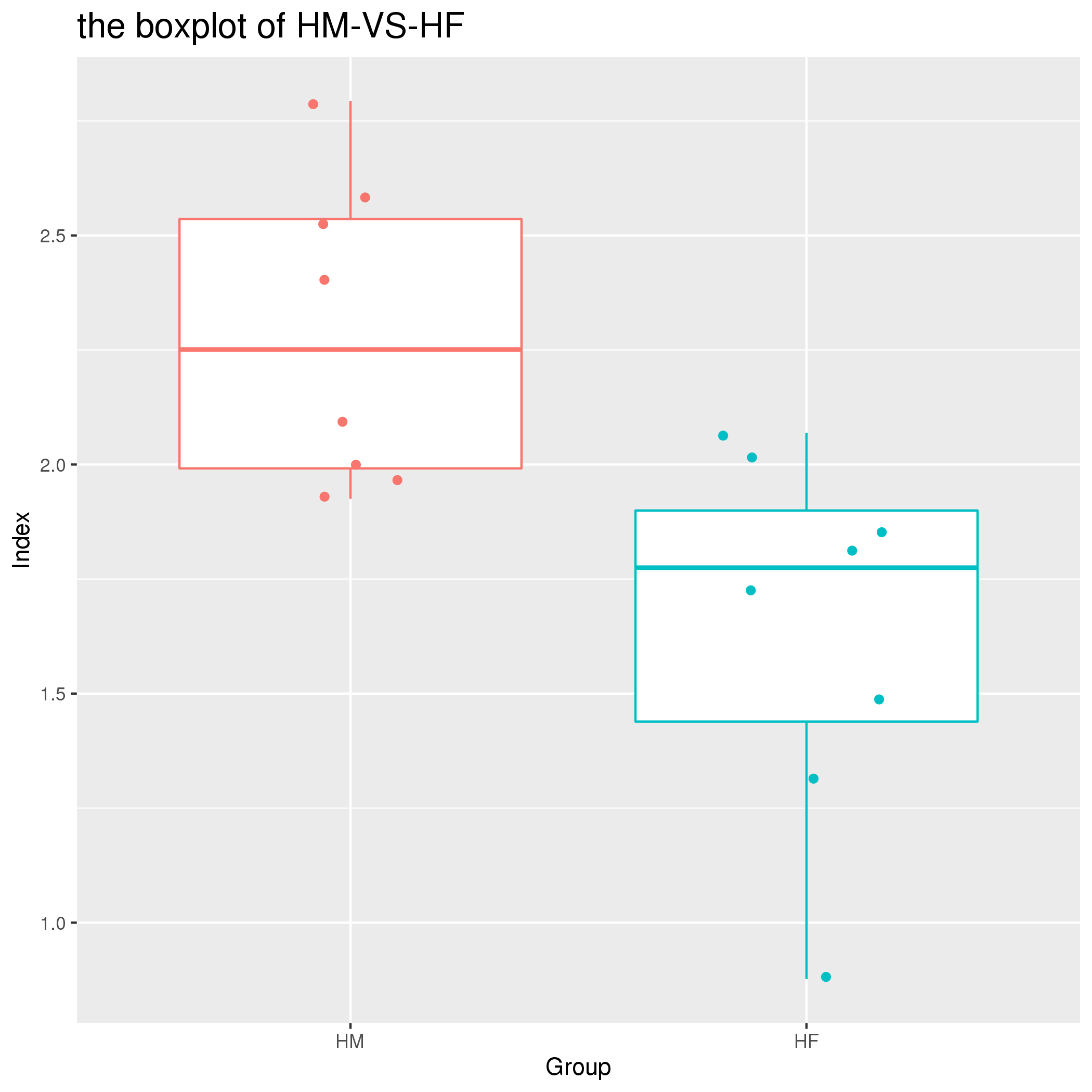

Supplement: Supplementary file 1 [file biology-12-00212-s001.zip › 16s rDNA SEQ/3.Alpha_diversity/2.diff_alpha_diversity/shannon/HM-VS-HF.boxplot.png]

the boxplot of CF-VS-HF

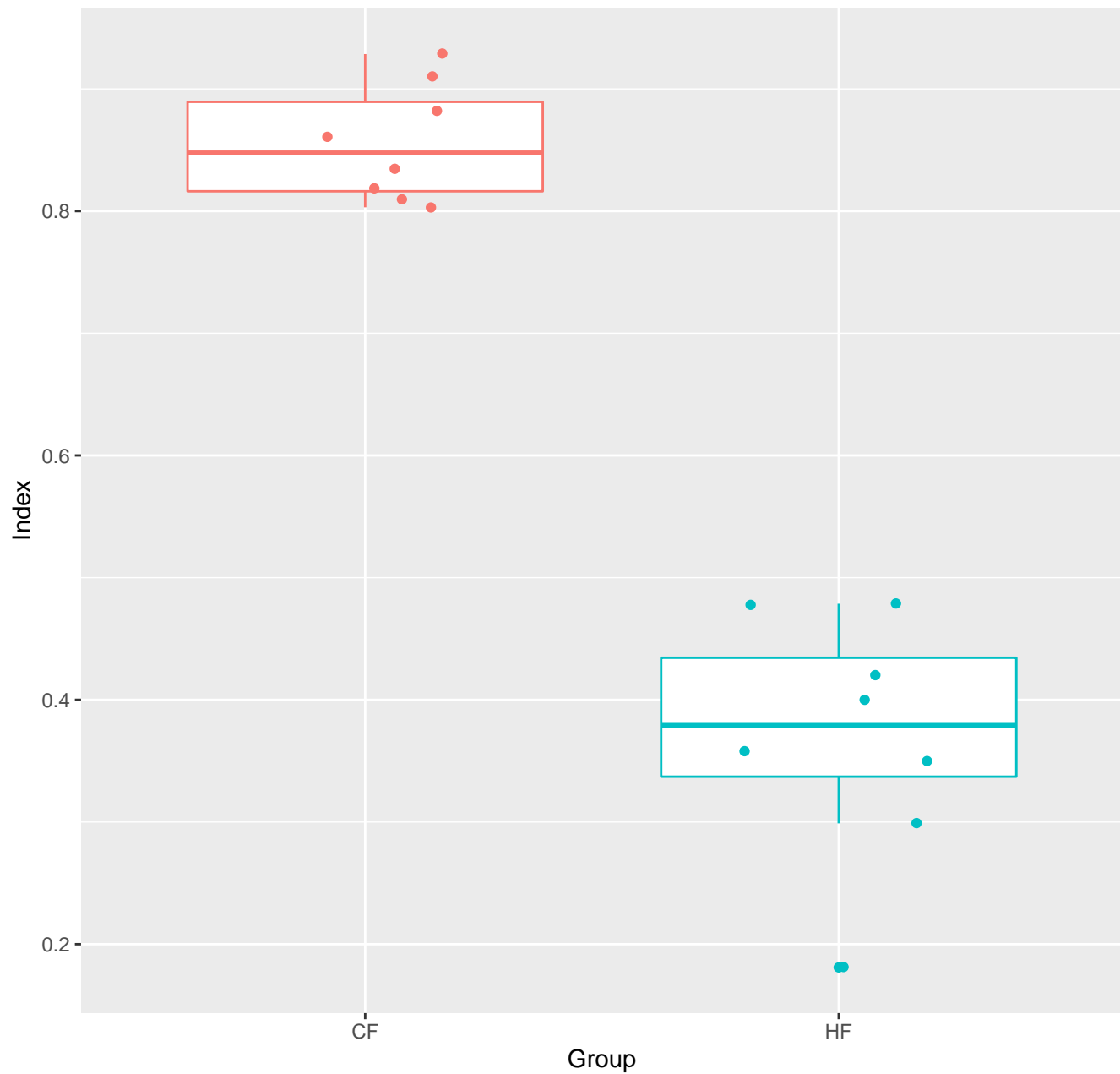

Supplement: Supplementary file 1 [file biology-12-00212-s001.zip › 16s rDNA SEQ/3.Alpha_diversity/2.diff_alpha_diversity/simpson/CF-VS-HF.boxplot.pdf]

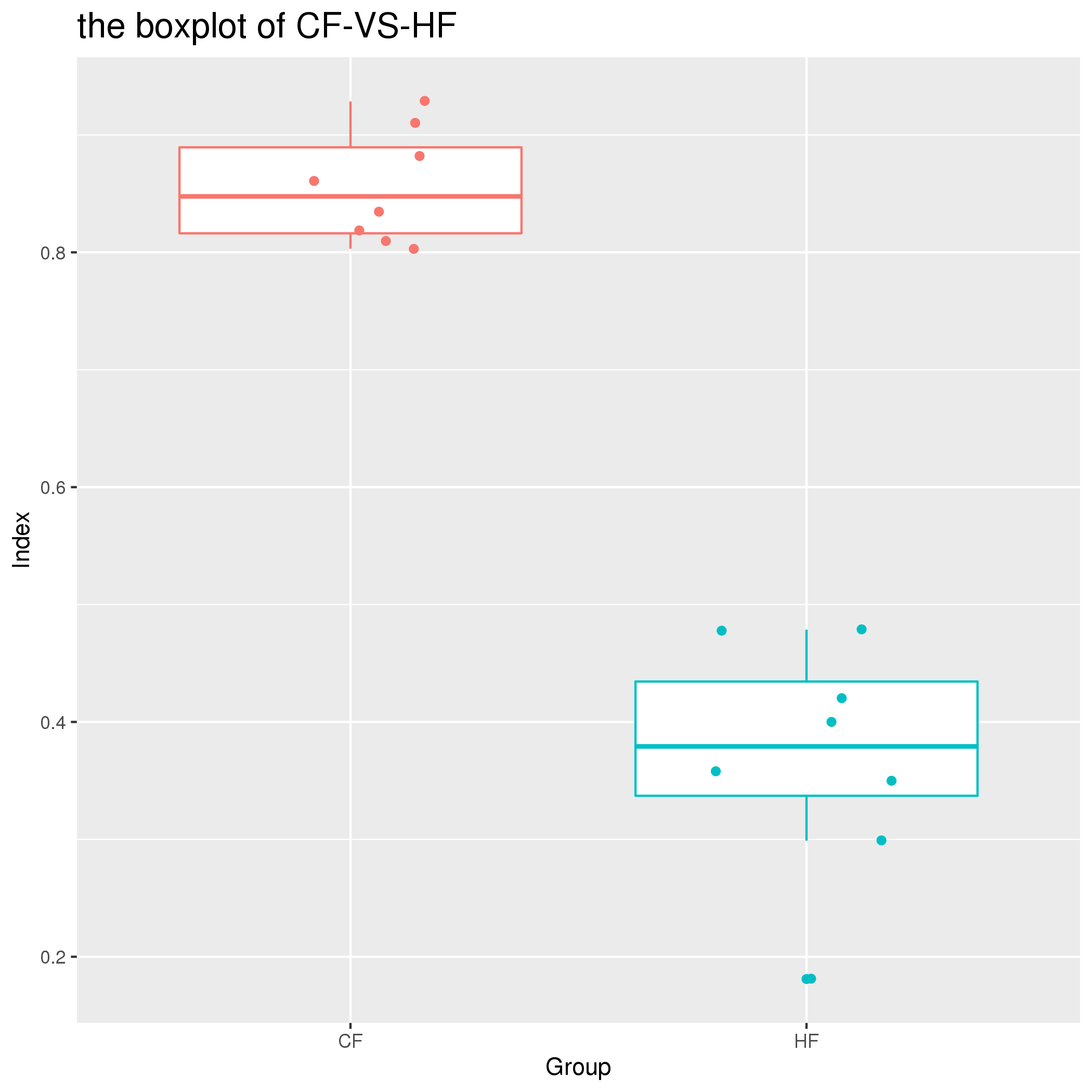

Supplement: Supplementary file 1 [file biology-12-00212-s001.zip › 16s rDNA SEQ/3.Alpha_diversity/2.diff_alpha_diversity/simpson/CF-VS-HF.boxplot.png]

the boxplot of CM-VS-CF

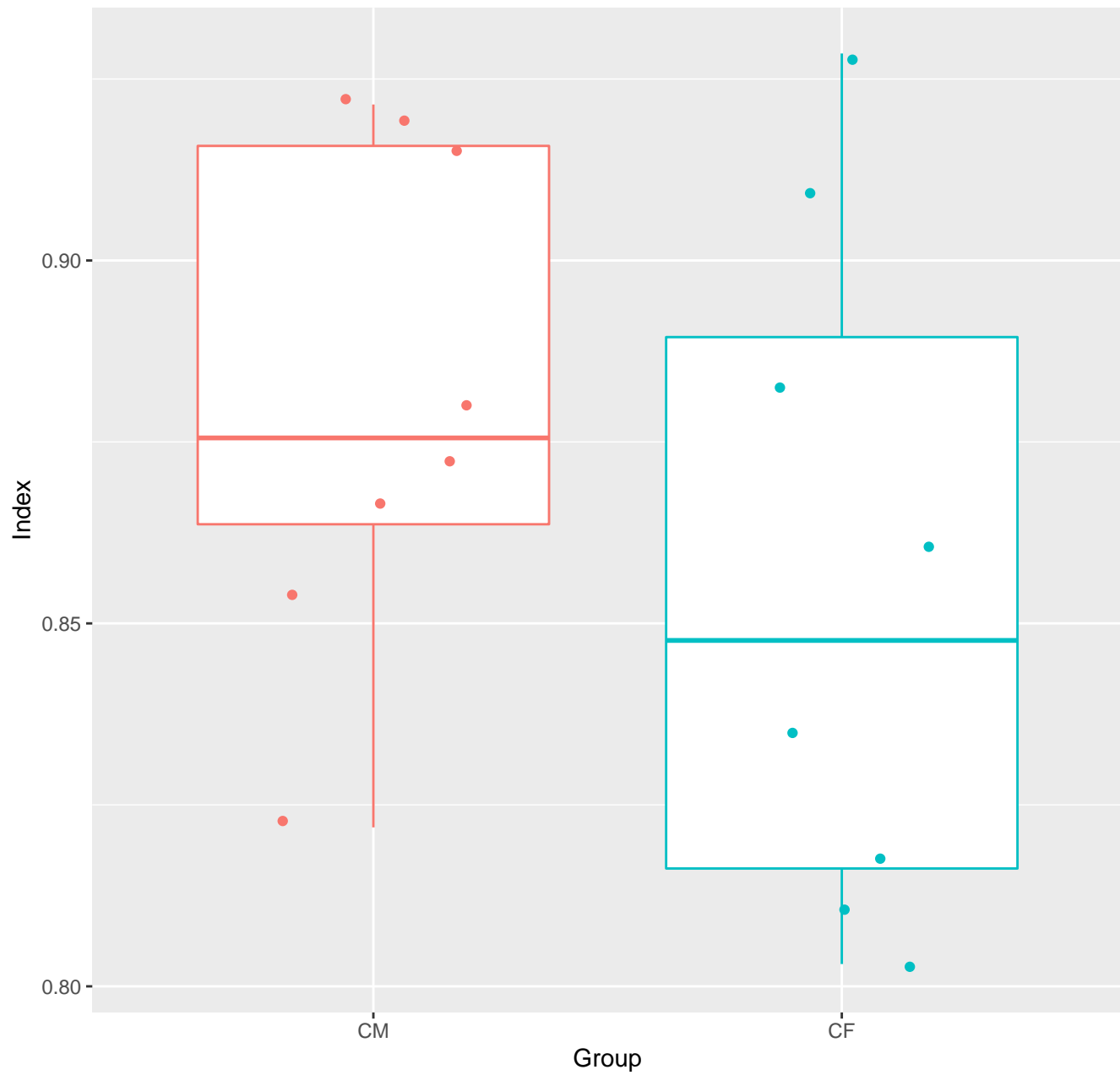

Supplement: Supplementary file 1 [file biology-12-00212-s001.zip › 16s rDNA SEQ/3.Alpha_diversity/2.diff_alpha_diversity/simpson/CM-VS-CF.boxplot.pdf]

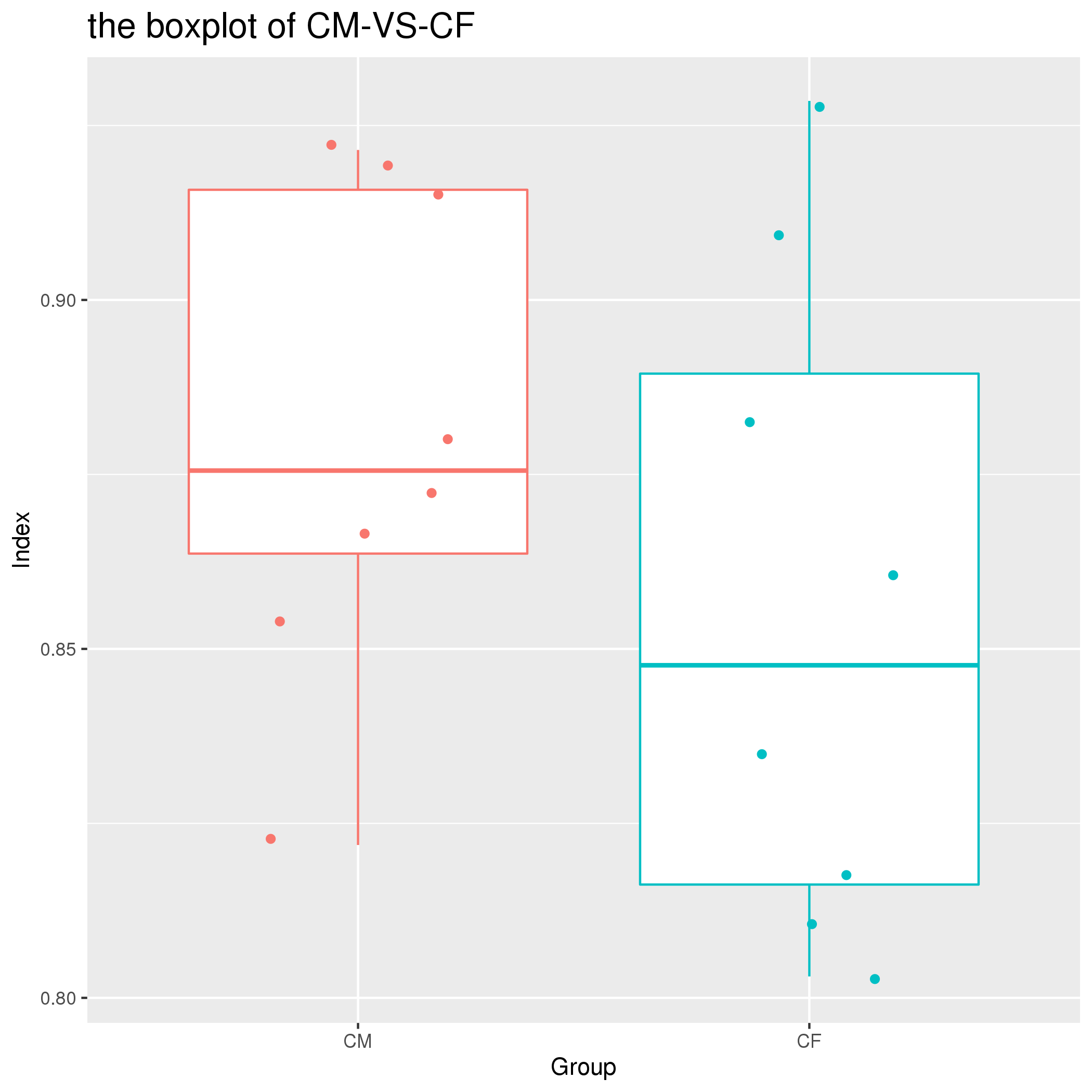

Supplement: Supplementary file 1 [file biology-12-00212-s001.zip › 16s rDNA SEQ/3.Alpha_diversity/2.diff_alpha_diversity/simpson/CM-VS-CF.boxplot.png]

the boxplot of CM-VS-HM

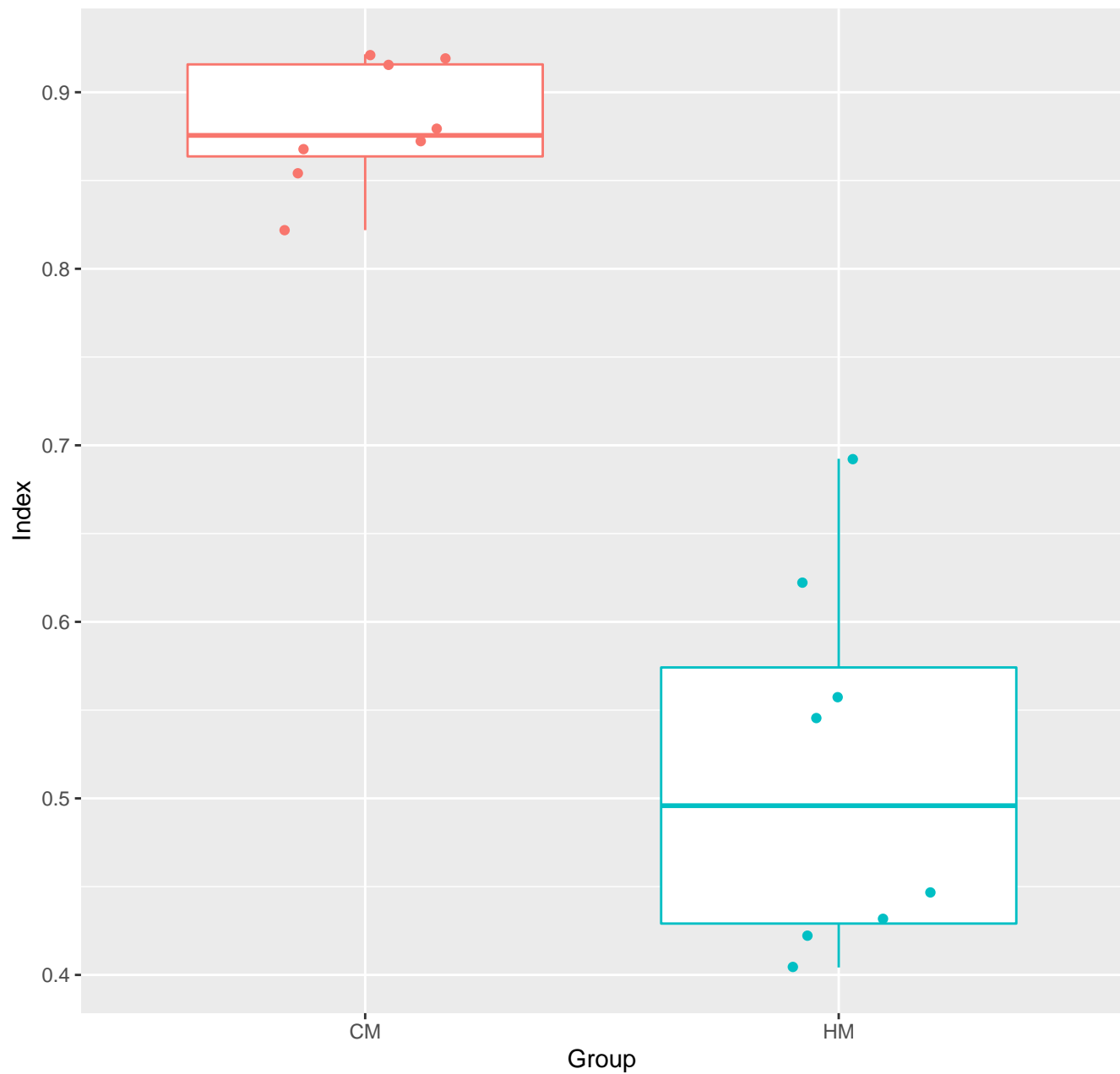

Supplement: Supplementary file 1 [file biology-12-00212-s001.zip › 16s rDNA SEQ/3.Alpha_diversity/2.diff_alpha_diversity/simpson/CM-VS-HM.boxplot.pdf]

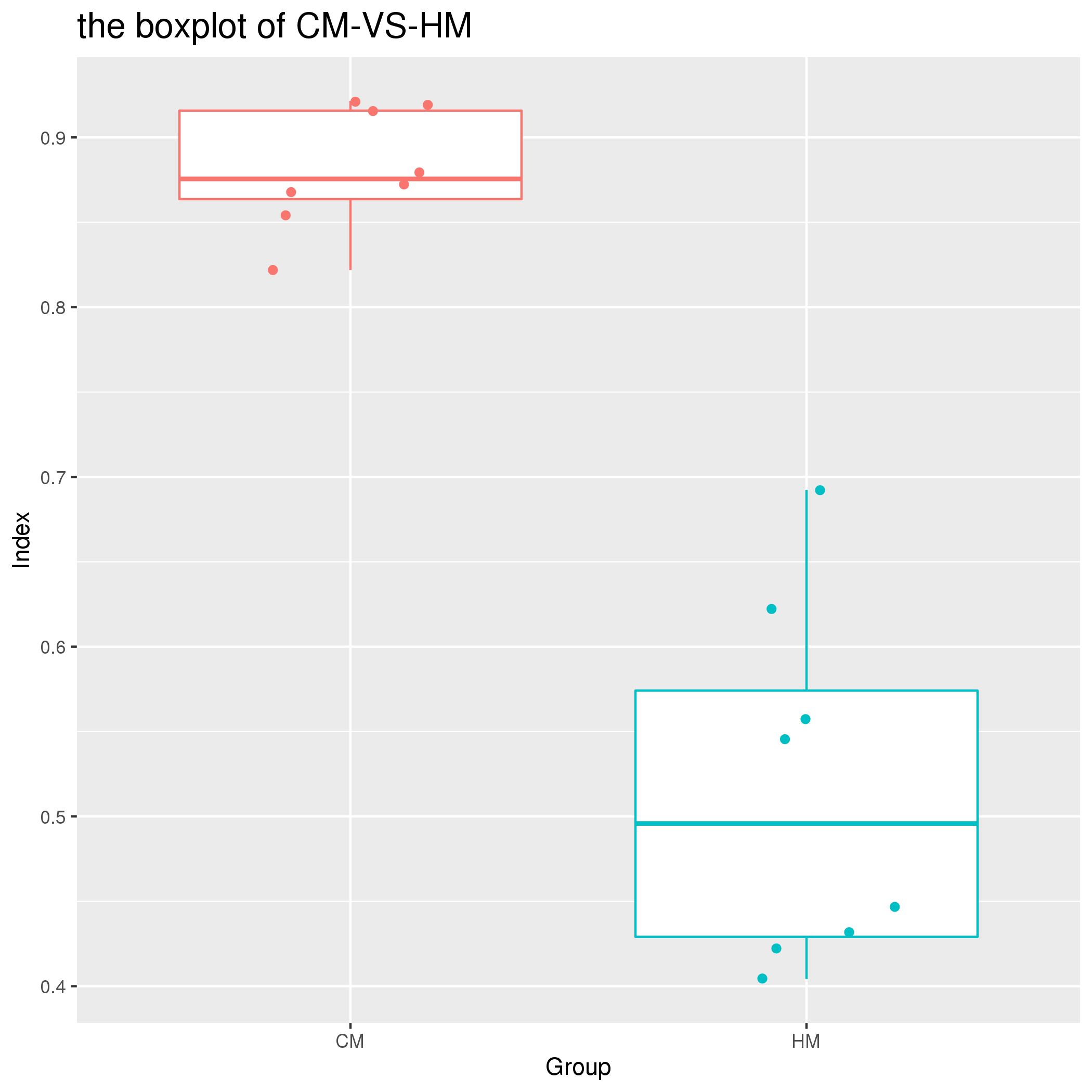

Supplement: Supplementary file 1 [file biology-12-00212-s001.zip › 16s rDNA SEQ/3.Alpha_diversity/2.diff_alpha_diversity/simpson/CM-VS-HM.boxplot.png]

the boxplot of HM-VS-HF

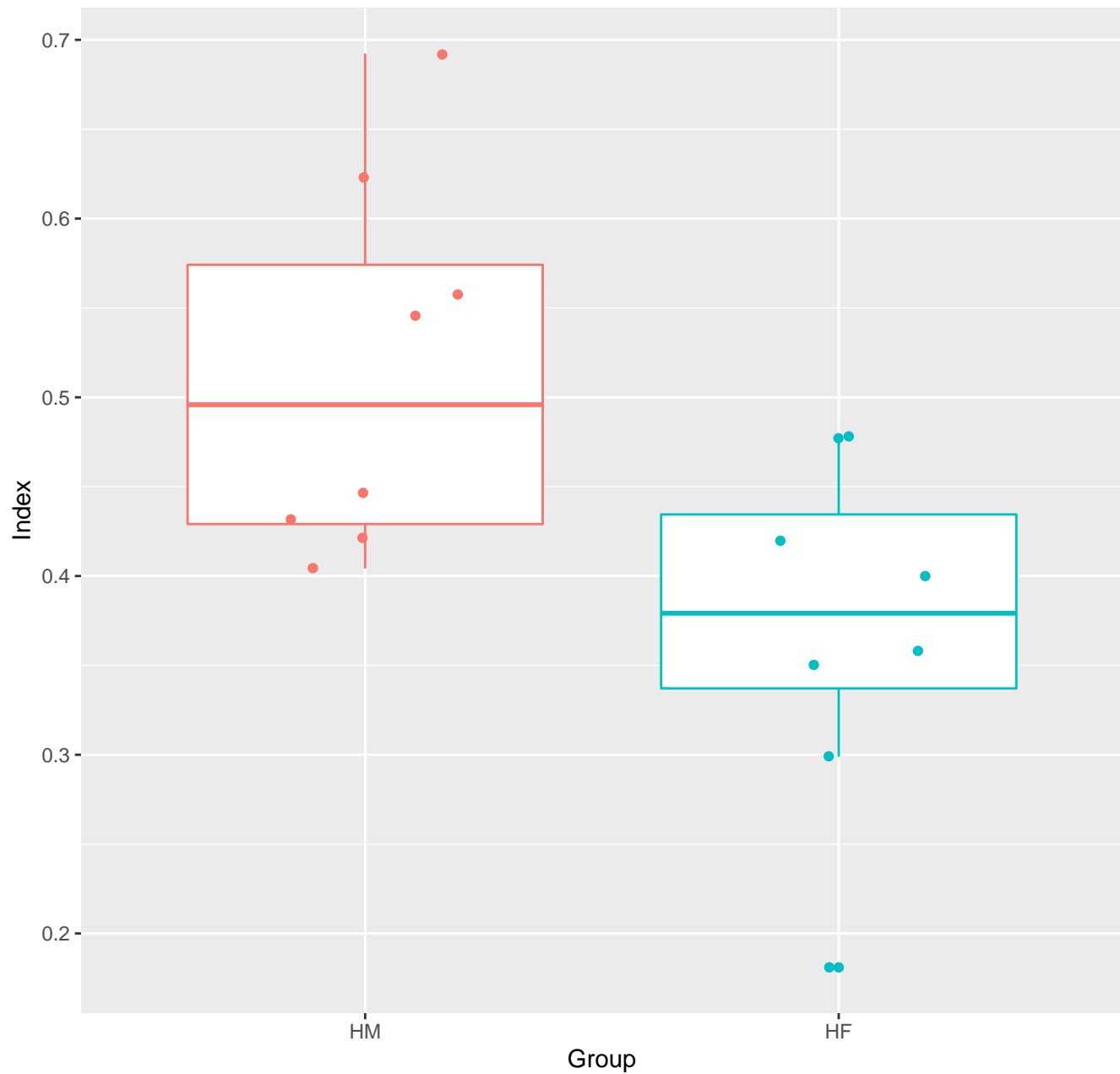

Supplement: Supplementary file 1 [file biology-12-00212-s001.zip › 16s rDNA SEQ/3.Alpha_diversity/2.diff_alpha_diversity/simpson/HM-VS-HF.boxplot.pdf]

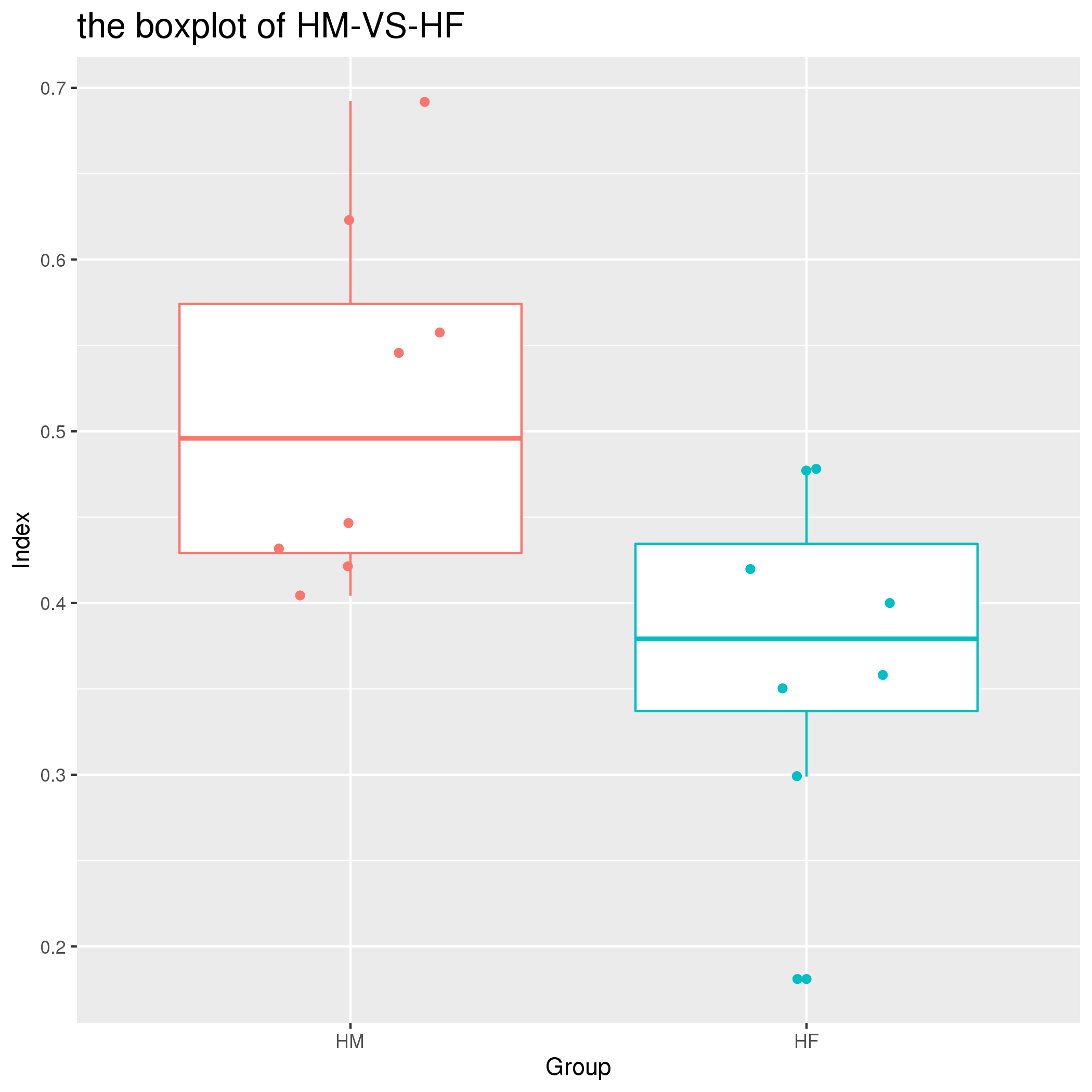

Supplement: Supplementary file 1 [file biology-12-00212-s001.zip › 16s rDNA SEQ/3.Alpha_diversity/2.diff_alpha_diversity/simpson/HM-VS-HF.boxplot.png]

the boxplot of CF-VS-HF

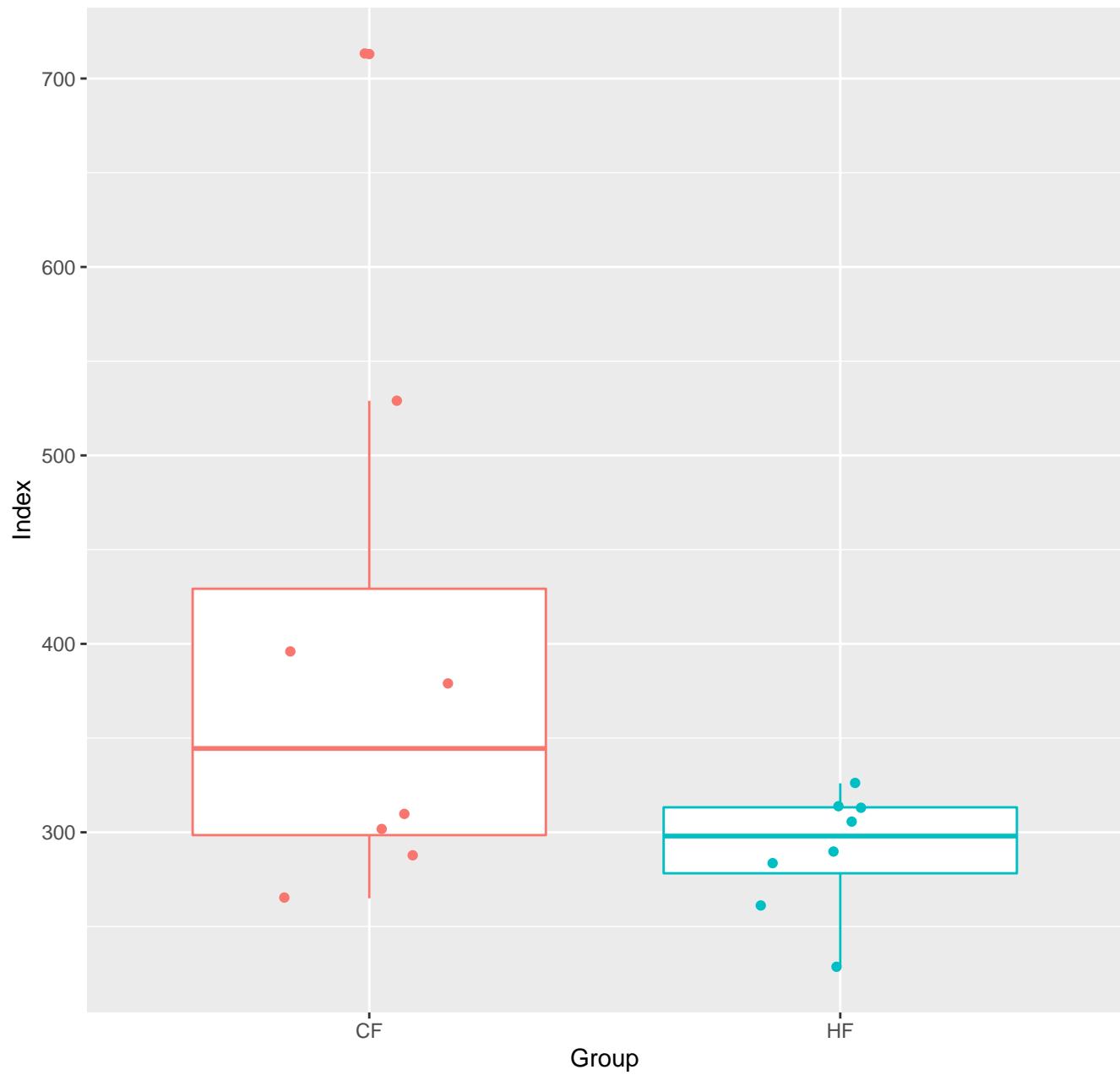

Supplement: Supplementary file 1 [file biology-12-00212-s001.zip › 16s rDNA SEQ/3.Alpha_diversity/2.diff_alpha_diversity/sobs/CF-VS-HF.boxplot.pdf]

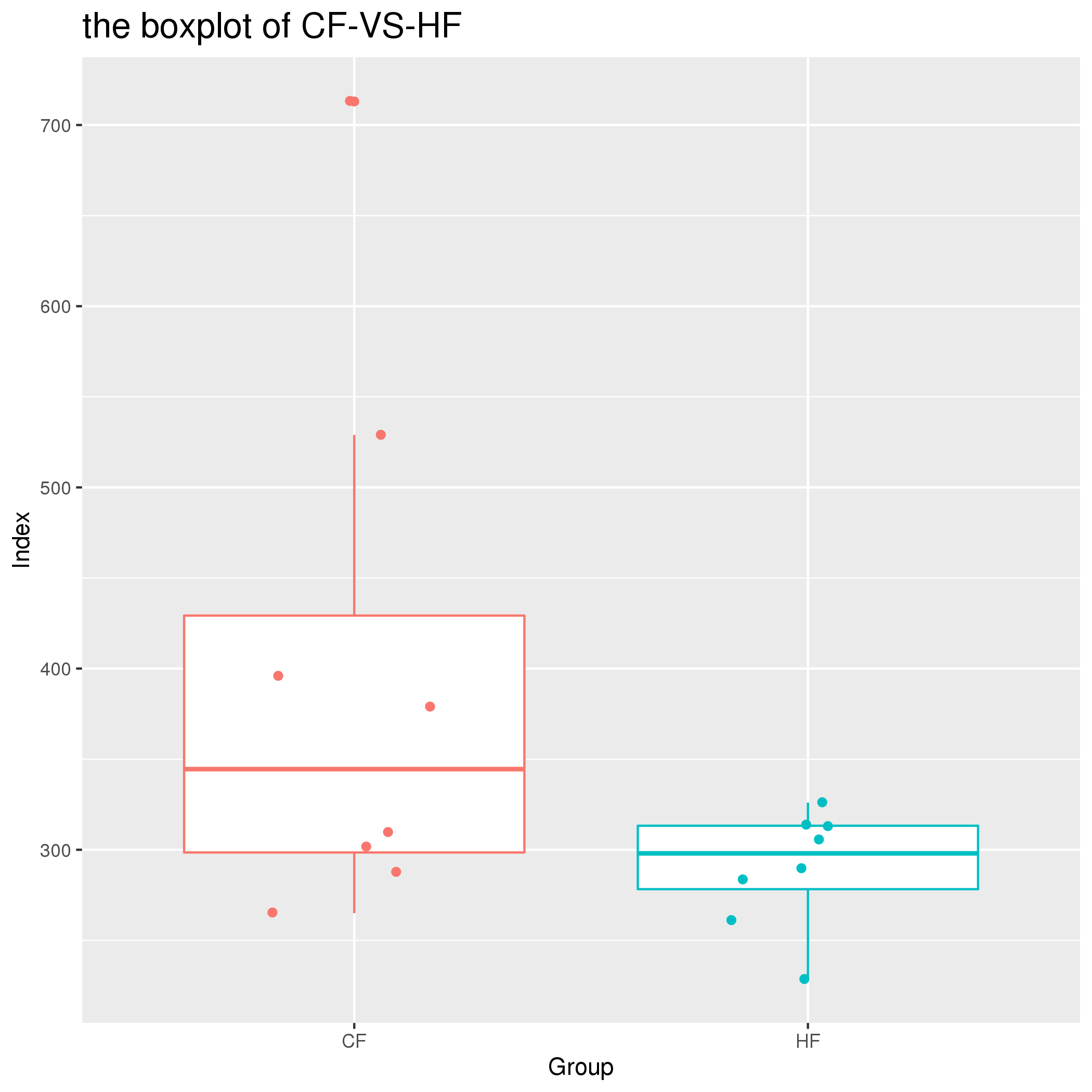

Supplement: Supplementary file 1 [file biology-12-00212-s001.zip › 16s rDNA SEQ/3.Alpha_diversity/2.diff_alpha_diversity/sobs/CF-VS-HF.boxplot.png]

the boxplot of CM-VS-CF

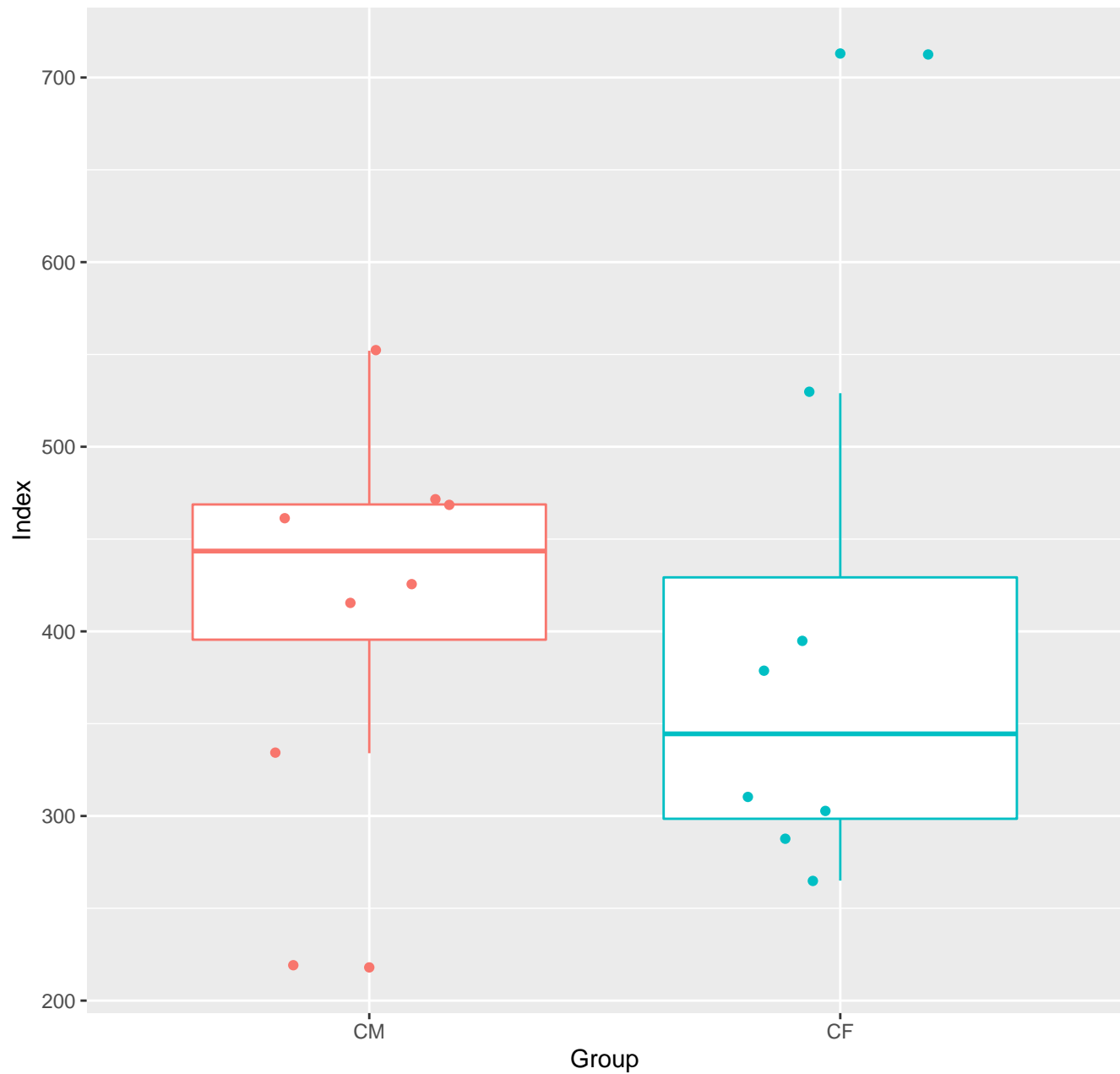

Supplement: Supplementary file 1 [file biology-12-00212-s001.zip › 16s rDNA SEQ/3.Alpha_diversity/2.diff_alpha_diversity/sobs/CM-VS-CF.boxplot.pdf]

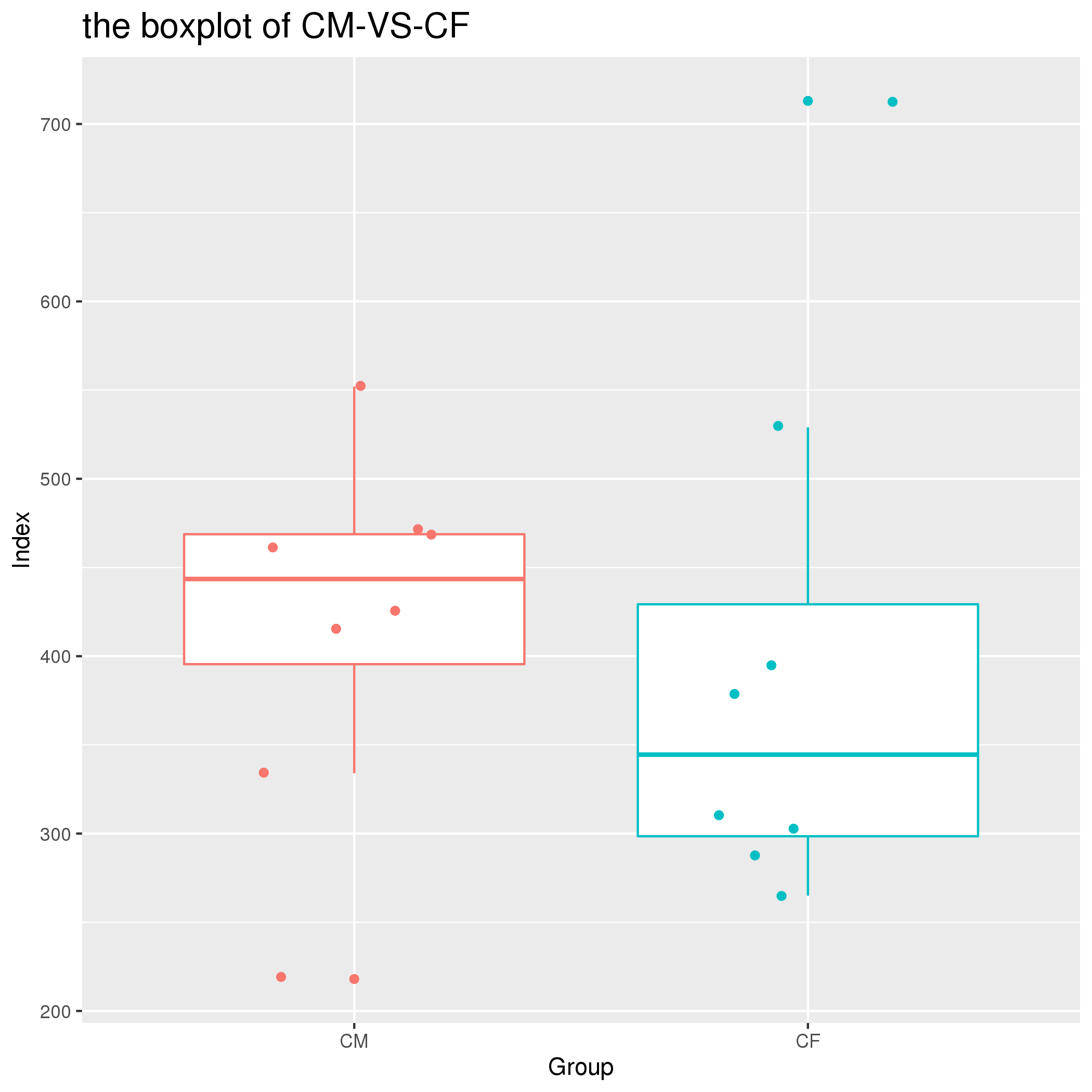

Supplement: Supplementary file 1 [file biology-12-00212-s001.zip › 16s rDNA SEQ/3.Alpha_diversity/2.diff_alpha_diversity/sobs/CM-VS-CF.boxplot.png]

the boxplot of CM-VS-HM

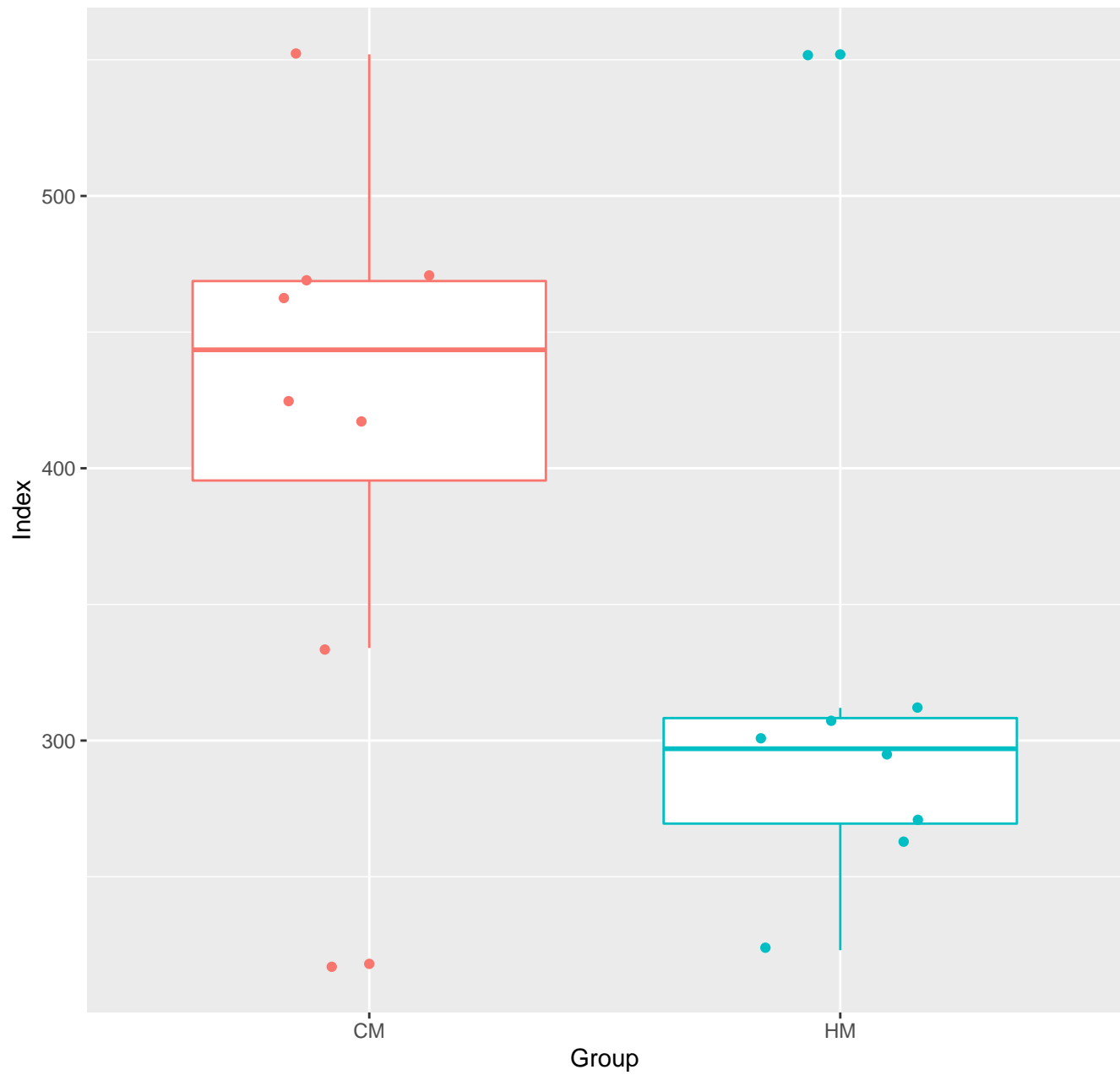

Supplement: Supplementary file 1 [file biology-12-00212-s001.zip › 16s rDNA SEQ/3.Alpha_diversity/2.diff_alpha_diversity/sobs/CM-VS-HM.boxplot.pdf]

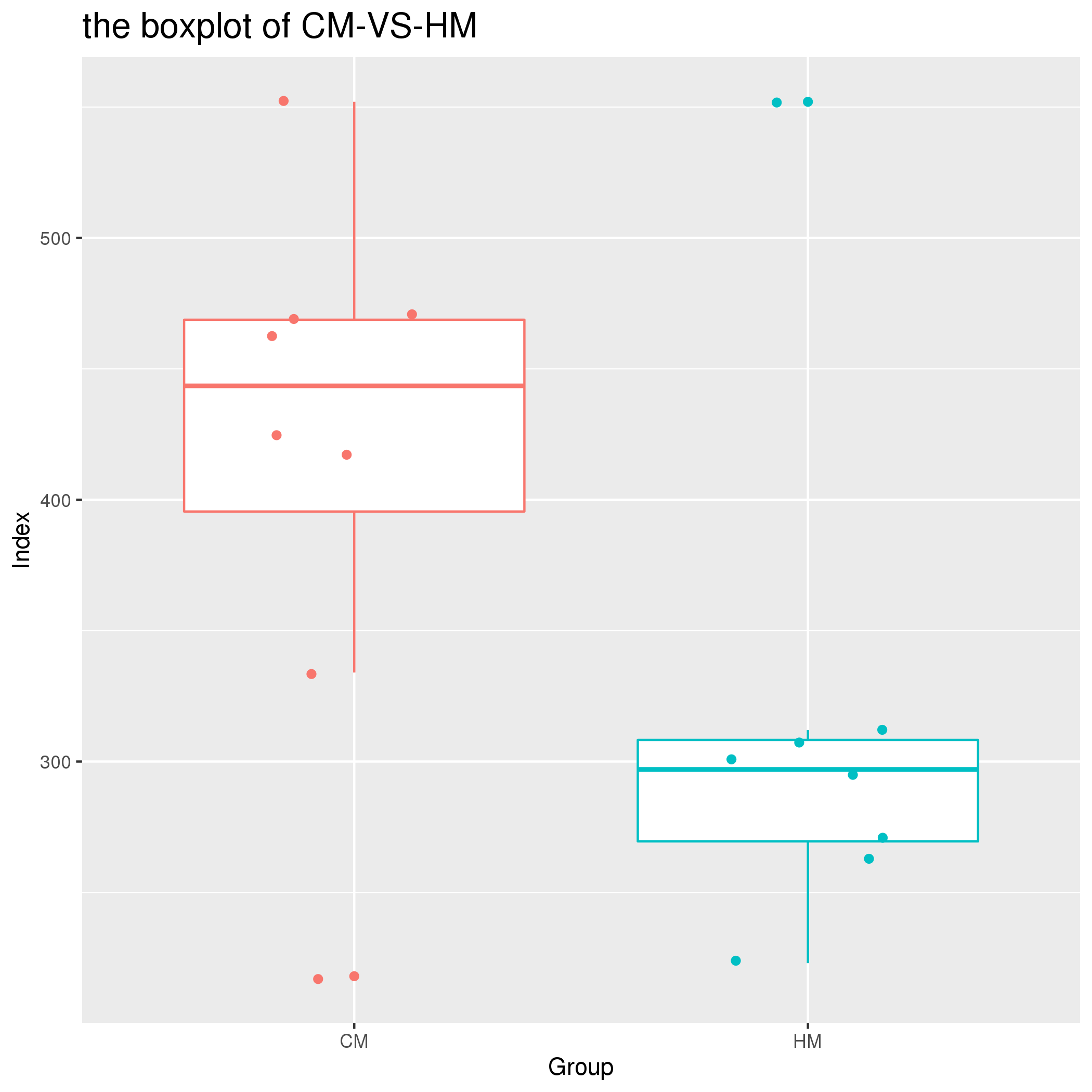

Supplement: Supplementary file 1 [file biology-12-00212-s001.zip › 16s rDNA SEQ/3.Alpha_diversity/2.diff_alpha_diversity/sobs/CM-VS-HM.boxplot.png]

the boxplot of HM-VS-HF

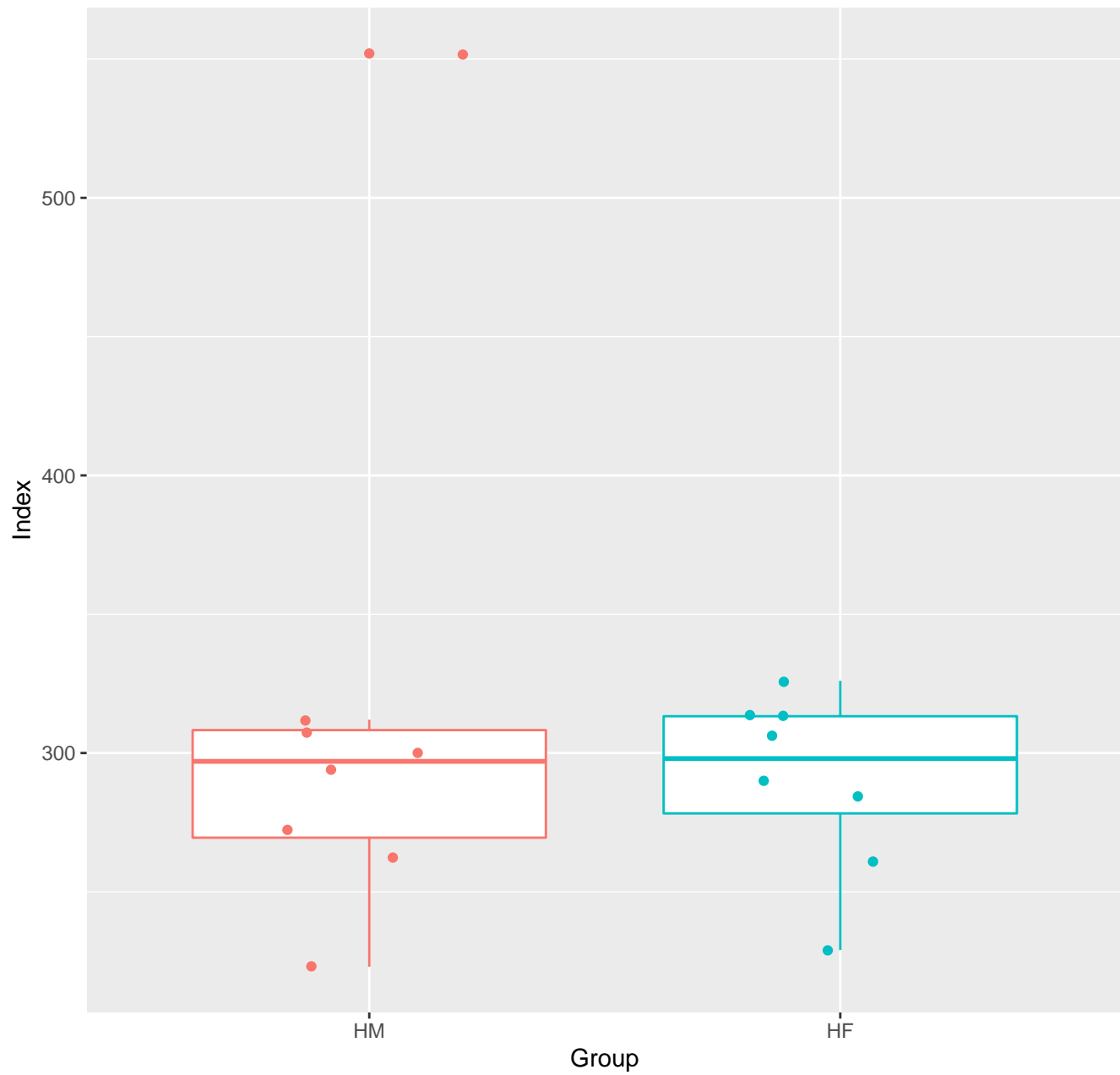

Supplement: Supplementary file 1 [file biology-12-00212-s001.zip › 16s rDNA SEQ/3.Alpha_diversity/2.diff_alpha_diversity/sobs/HM-VS-HF.boxplot.pdf]

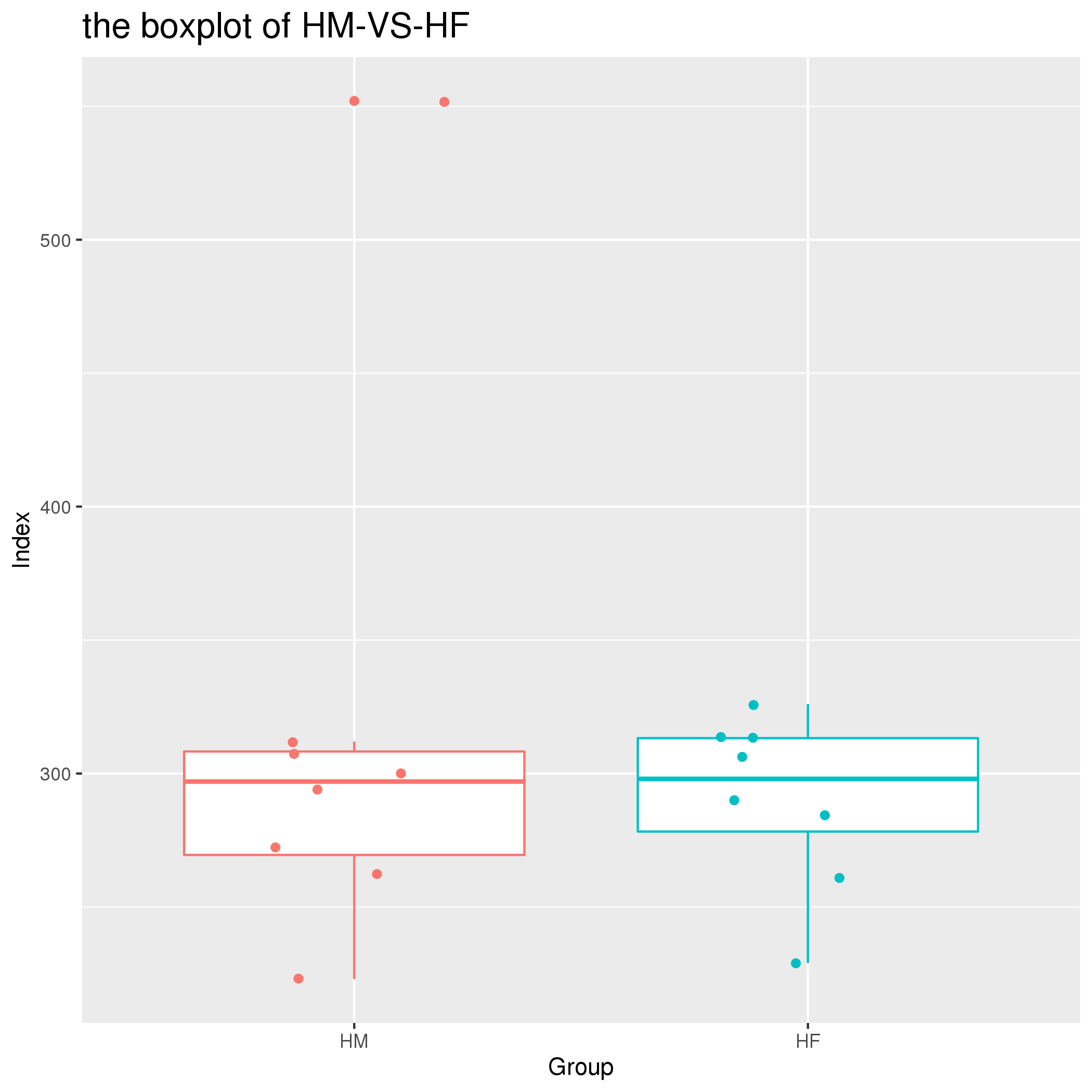

Supplement: Supplementary file 1 [file biology-12-00212-s001.zip › 16s rDNA SEQ/3.Alpha_diversity/2.diff_alpha_diversity/sobs/HM-VS-HF.boxplot.png]

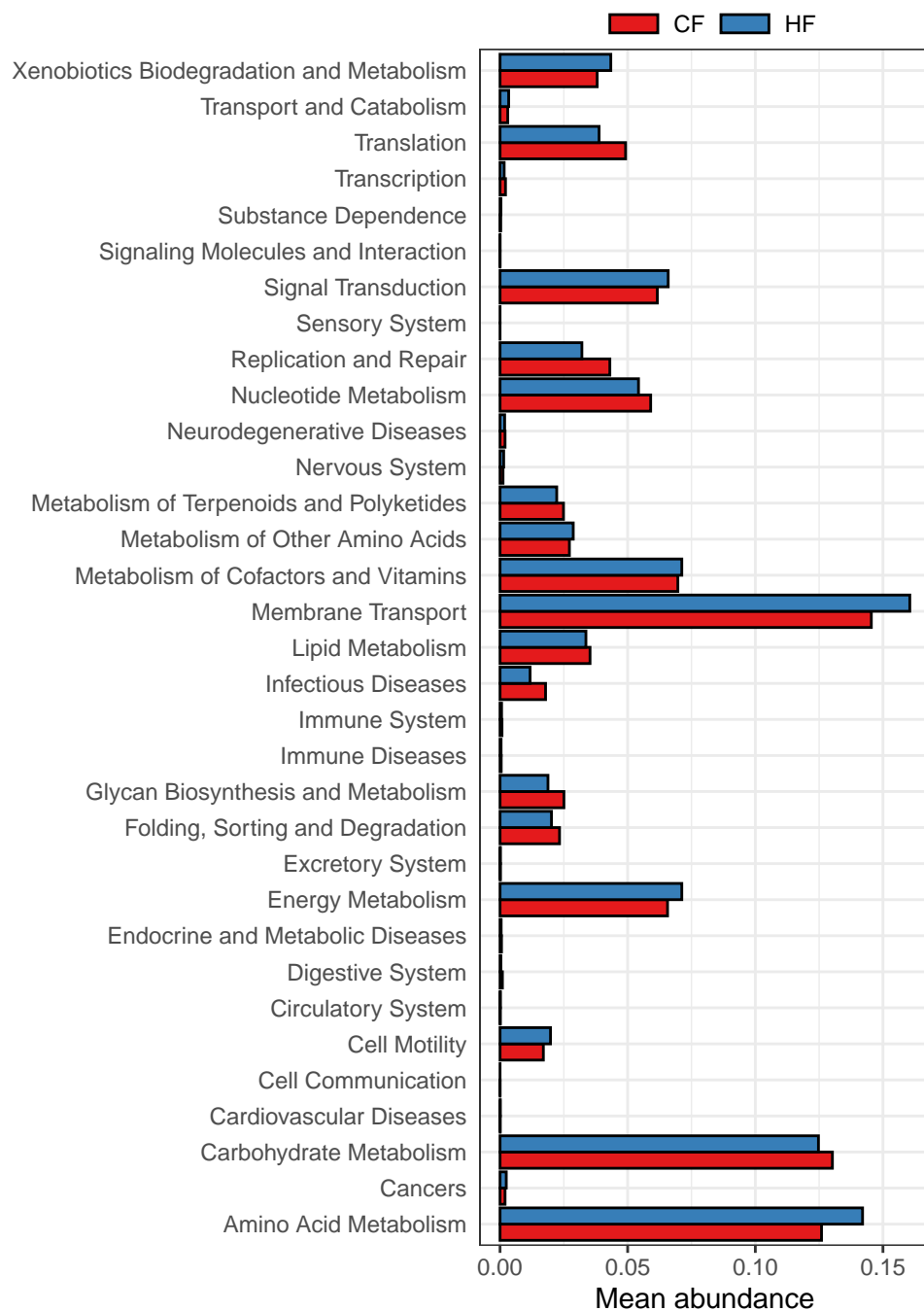

## 95% confidence intervals

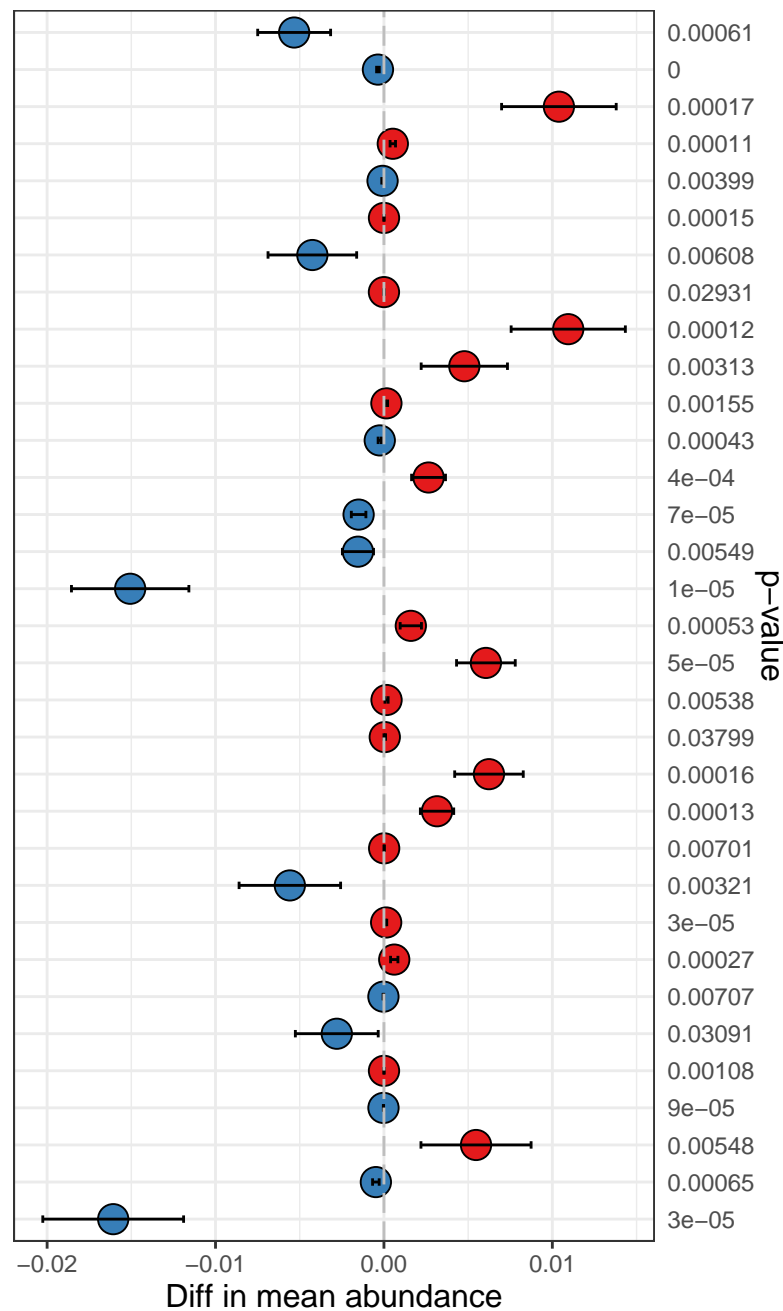

Supplement: Supplementary file 1 [file biology-12-00212-s001.zip › 16s rDNA SEQ/4.Tax4Fun/pathway_diff_L2/CF_vs_HF.t-test.extended_error_bar.pdf]

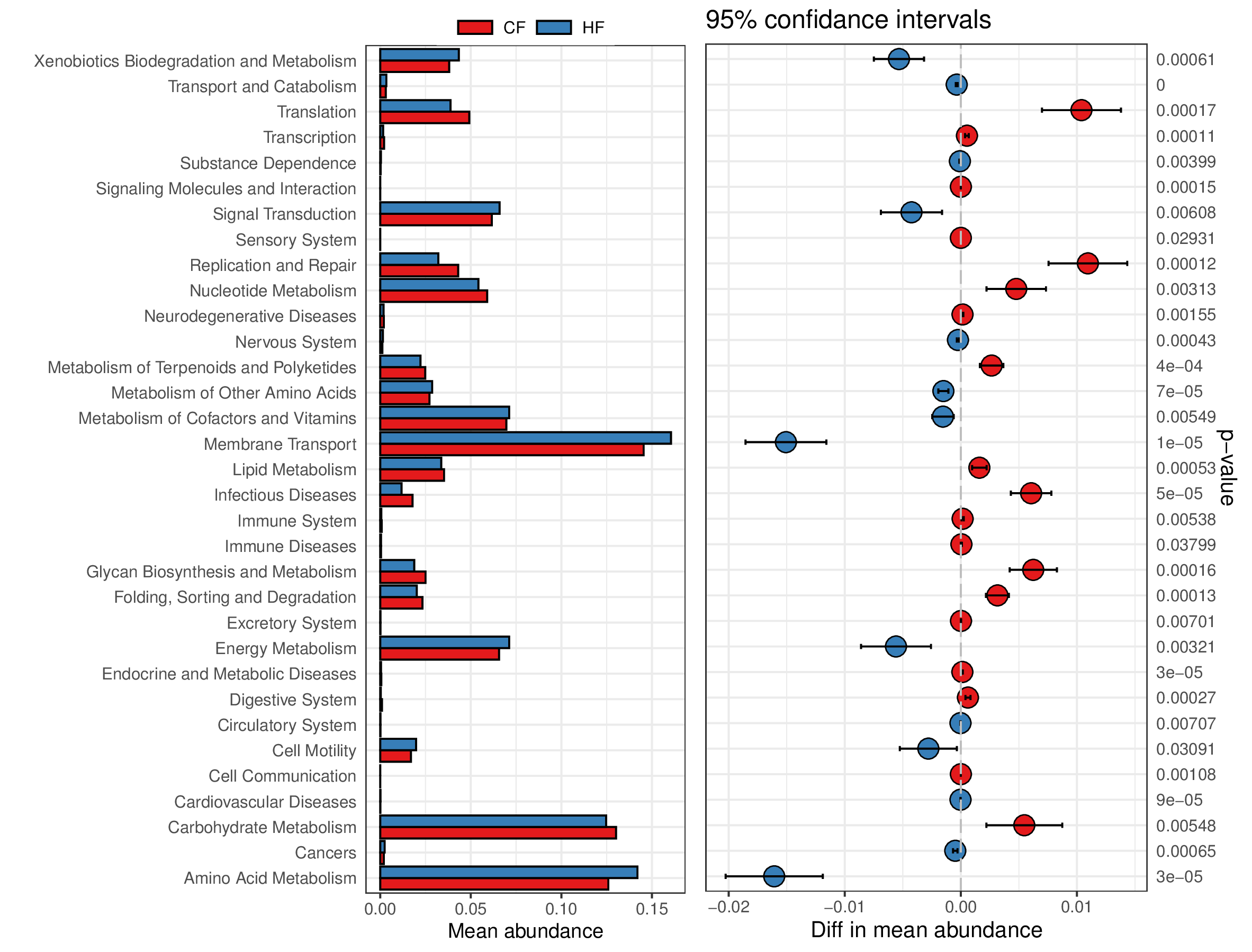

Supplement: Supplementary file 1 [file biology-12-00212-s001.zip › 16s rDNA SEQ/4.Tax4Fun/pathway_diff_L2/CF_vs_HF.t-test.extended_error_bar.png]

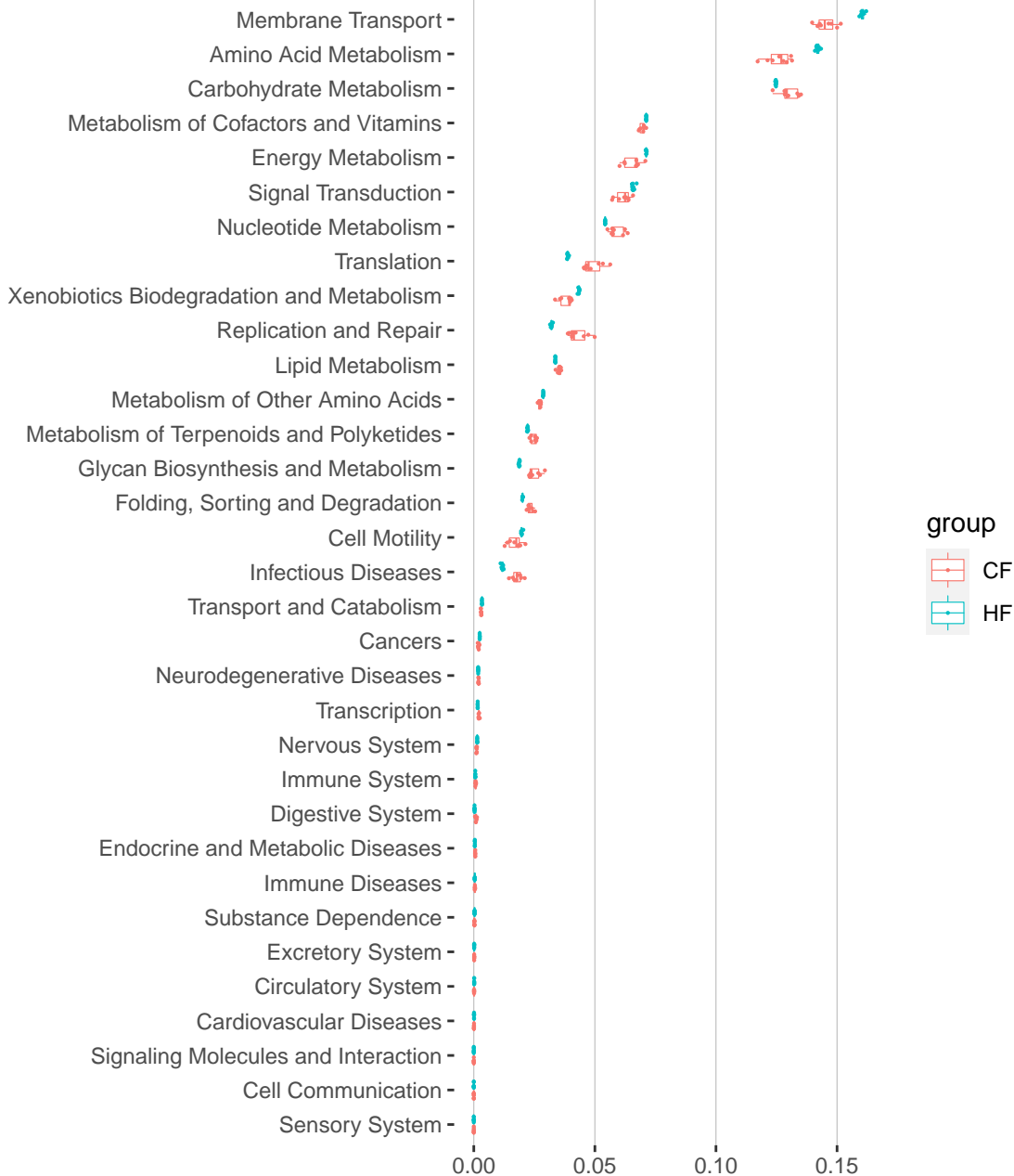

Supplement: Supplementary file 1 [file biology-12-00212-s001.zip › 16s rDNA SEQ/4.Tax4Fun/pathway_diff_L2/CF_vs_HF.wilcox.boxplot.pdf]

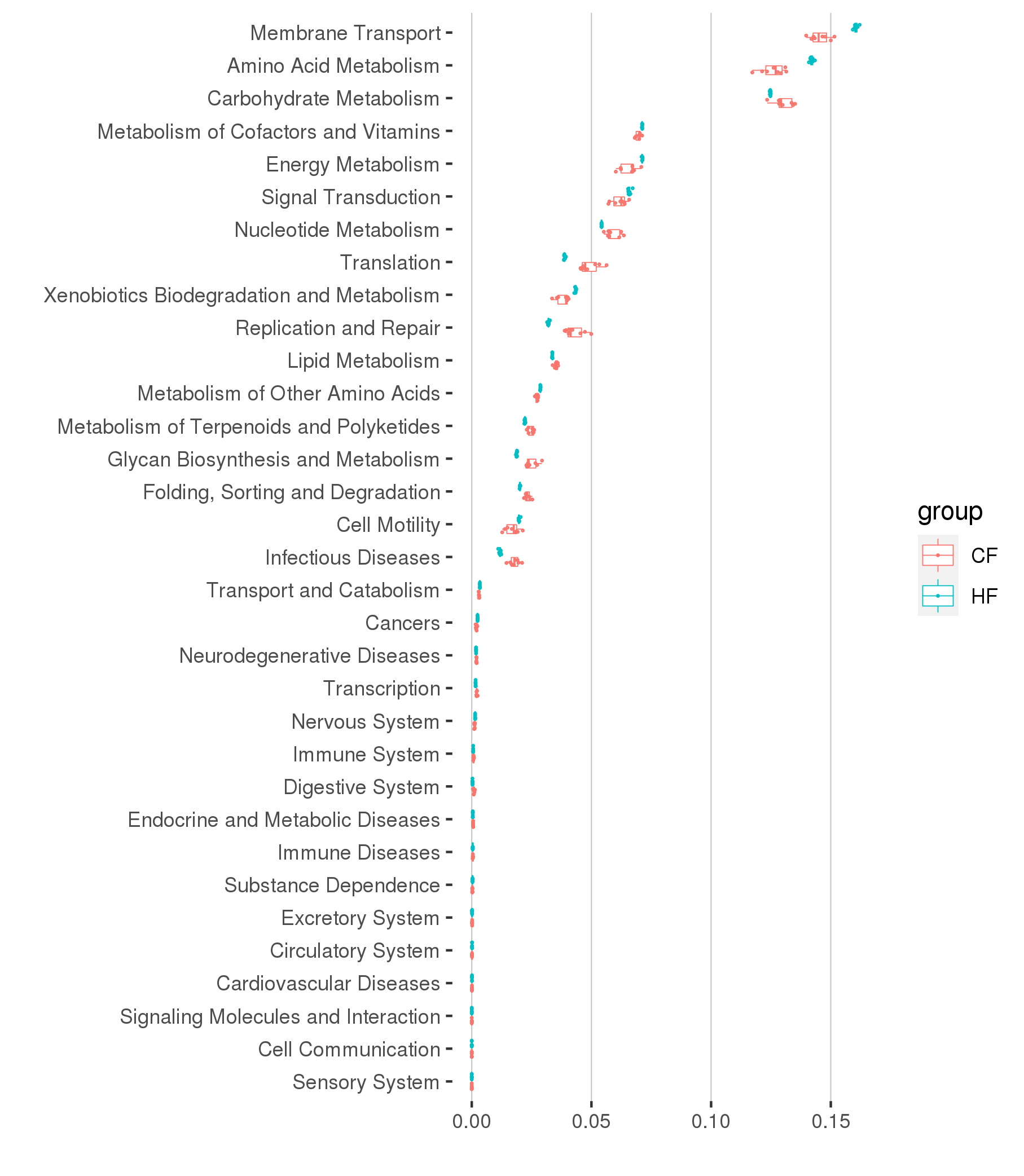

Supplement: Supplementary file 1 [file biology-12-00212-s001.zip › 16s rDNA SEQ/4.Tax4Fun/pathway_diff_L2/CF_vs_HF.wilcox.boxplot.png]

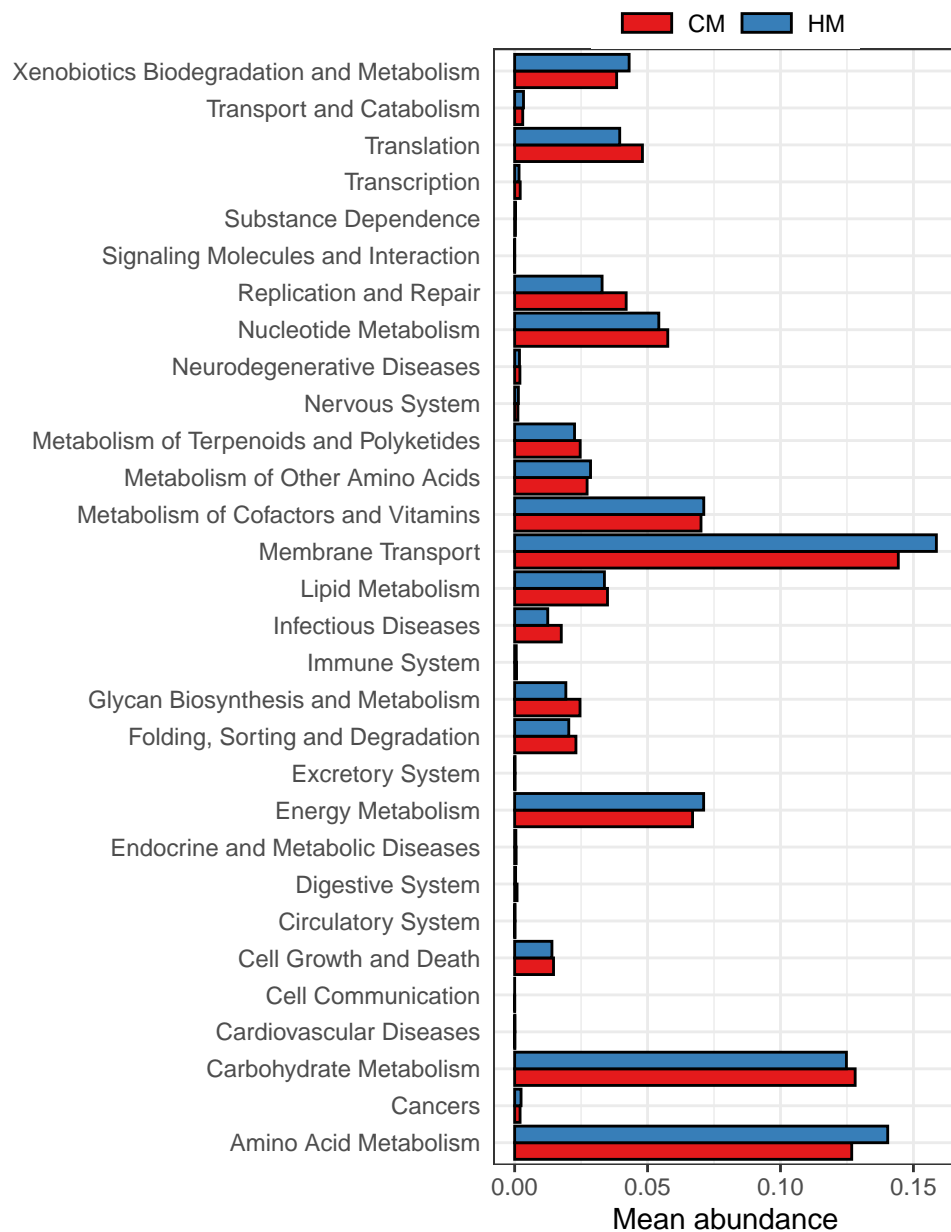

## 95% confidence intervals

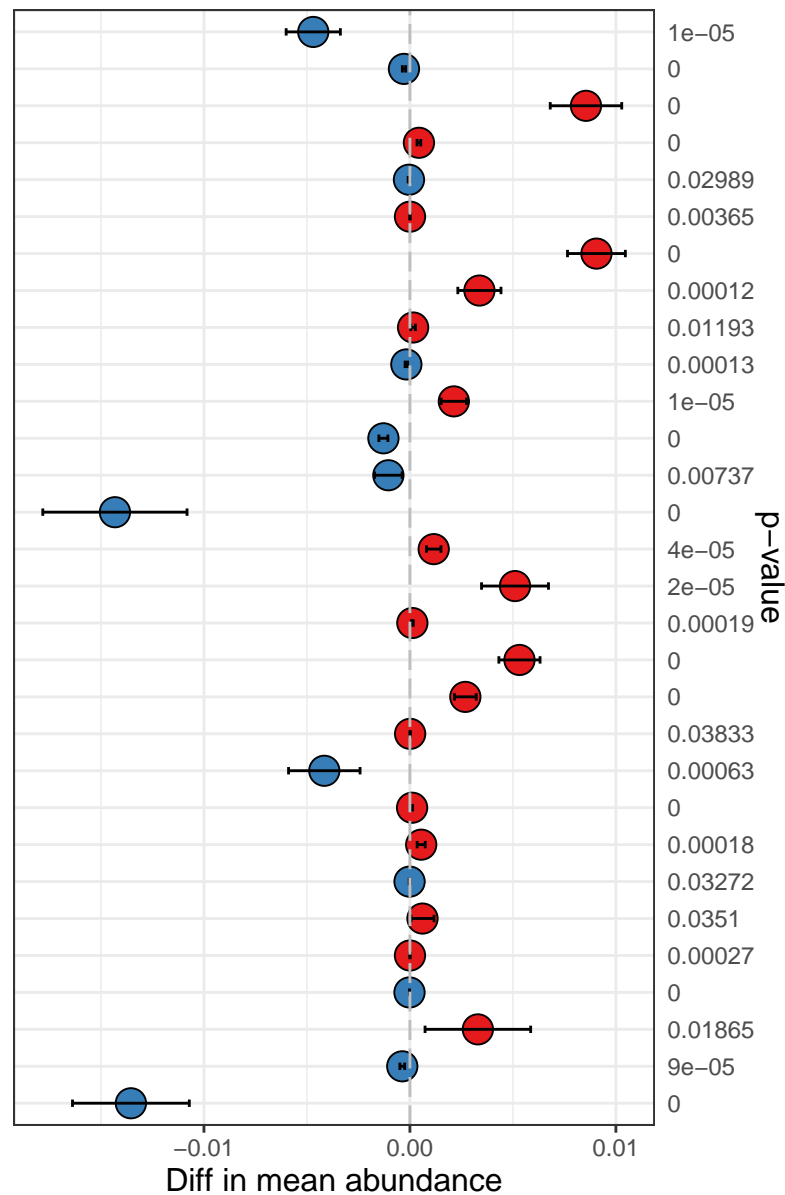

Supplement: Supplementary file 1 [file biology-12-00212-s001.zip › 16s rDNA SEQ/4.Tax4Fun/pathway_diff_L2/CM_vs_HM.t-test.extended_error_bar.pdf]

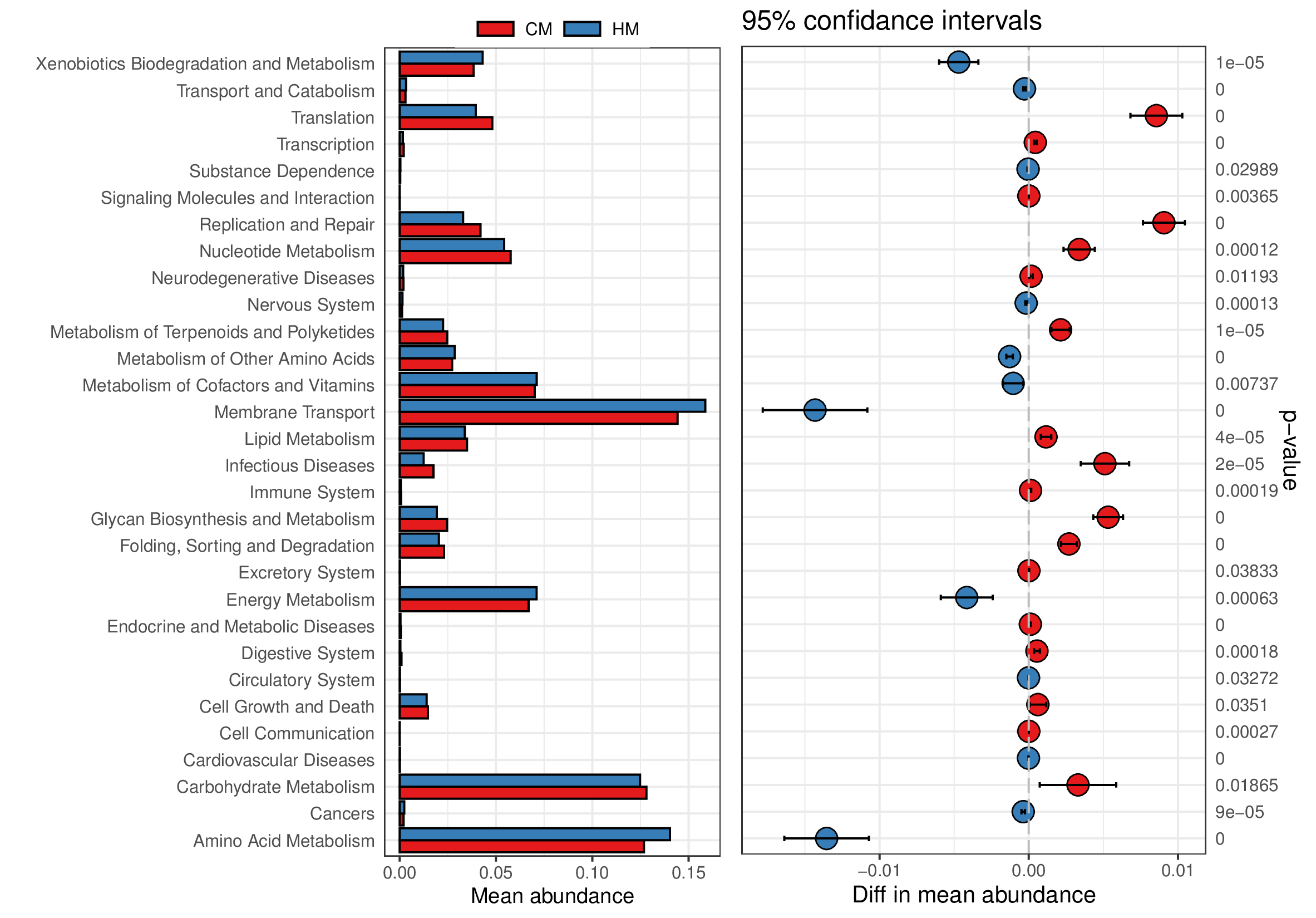

Supplement: Supplementary file 1 [file biology-12-00212-s001.zip › 16s rDNA SEQ/4.Tax4Fun/pathway_diff_L2/CM_vs_HM.t-test.extended_error_bar.png]

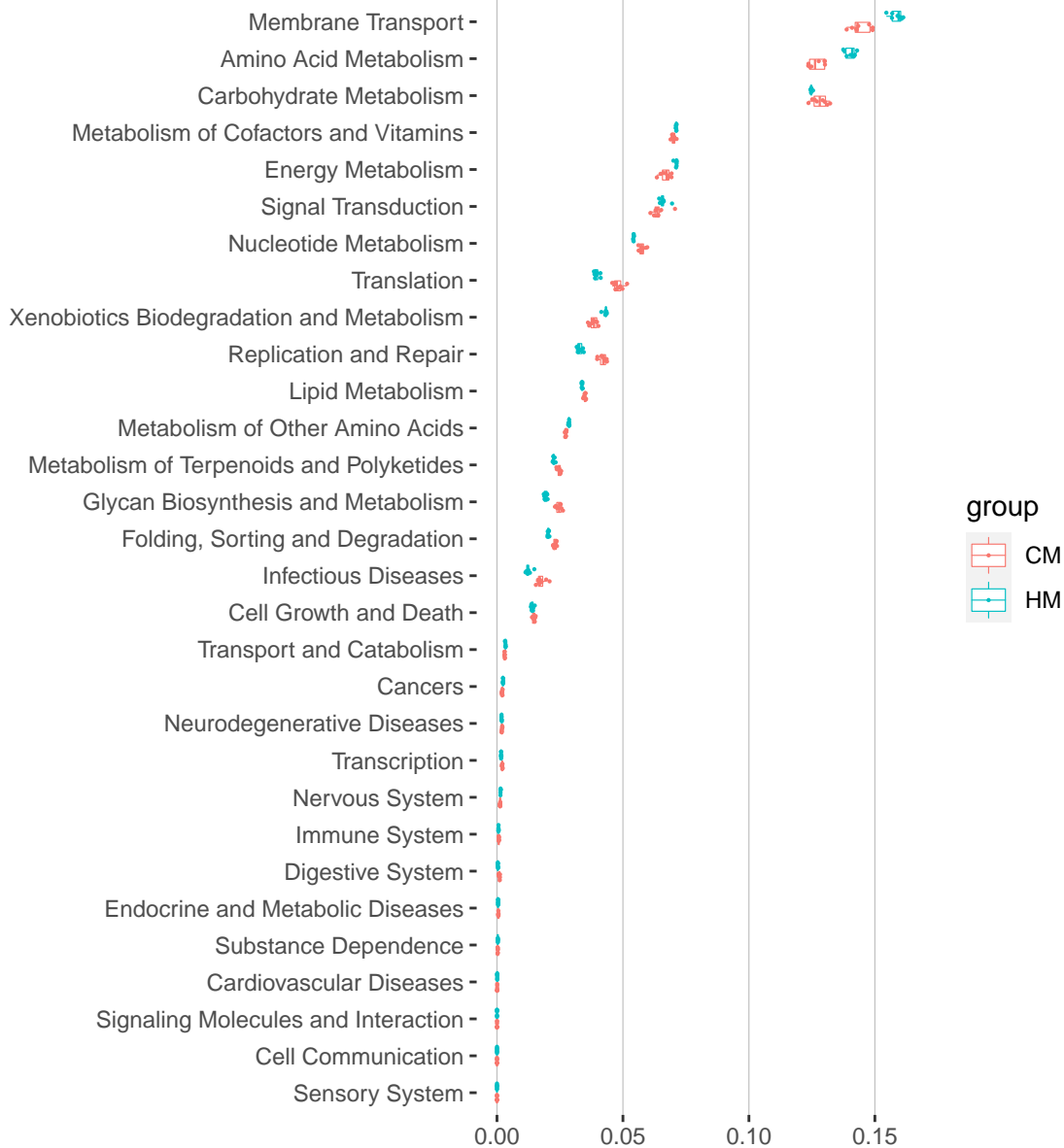

Supplement: Supplementary file 1 [file biology-12-00212-s001.zip › 16s rDNA SEQ/4.Tax4Fun/pathway_diff_L2/CM_vs_HM.wilcox.boxplot.pdf]

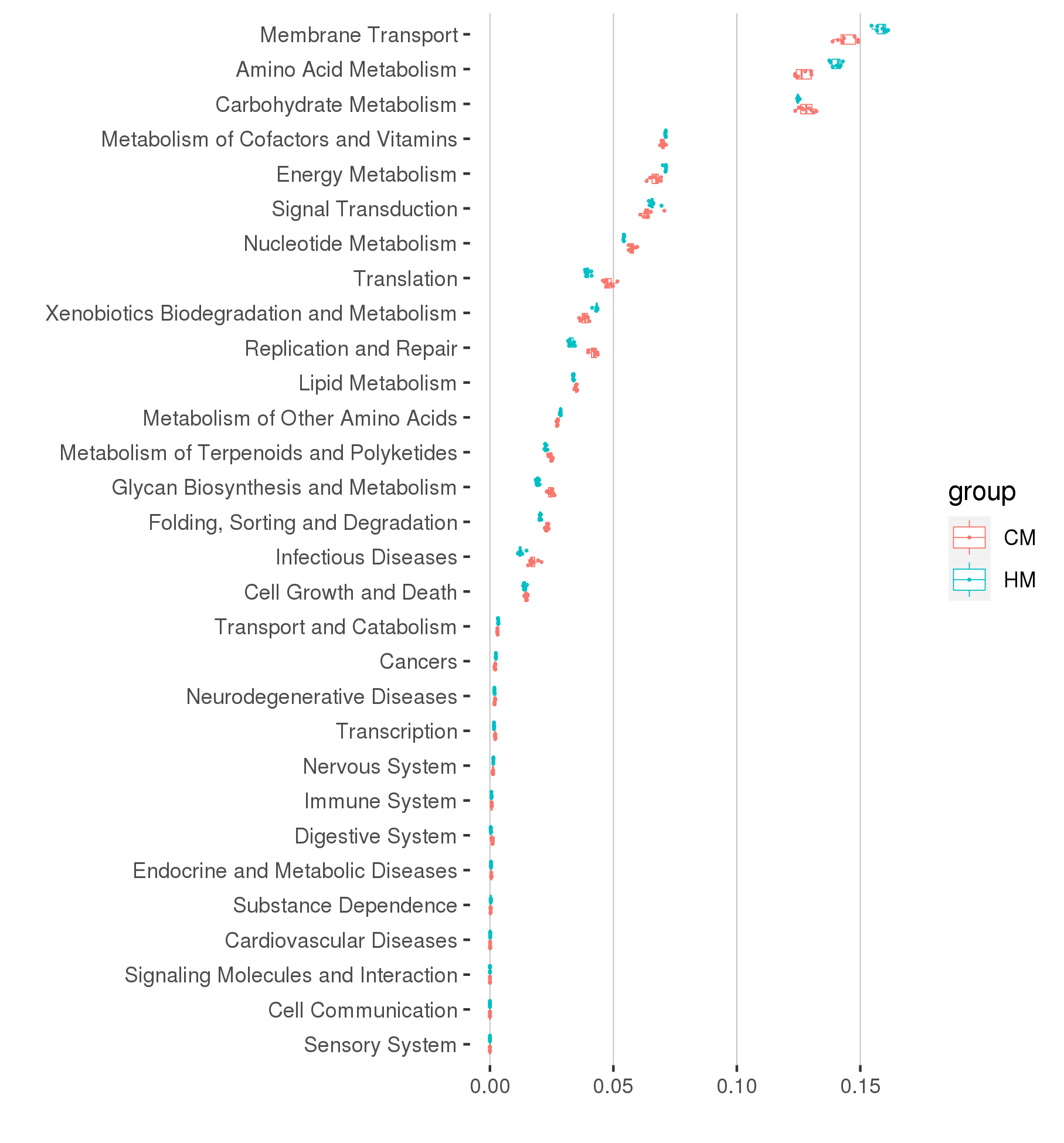

Supplement: Supplementary file 1 [file biology-12-00212-s001.zip › 16s rDNA SEQ/4.Tax4Fun/pathway_diff_L2/CM_vs_HM.wilcox.boxplot.png]

Metabolism of Terpenoids and Polyketides -

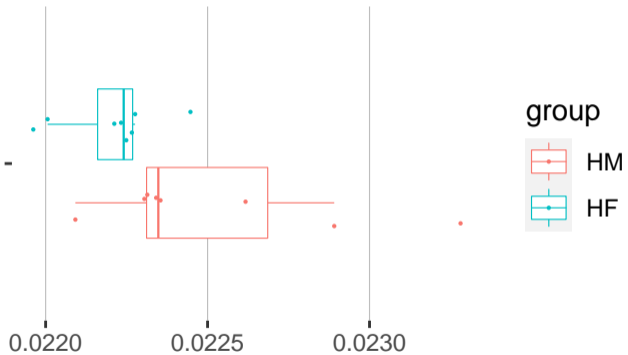

Supplement: Supplementary file 1 [file biology-12-00212-s001.zip › 16s rDNA SEQ/4.Tax4Fun/pathway_diff_L2/HM_vs_HF.wilcox.boxplot.pdf]

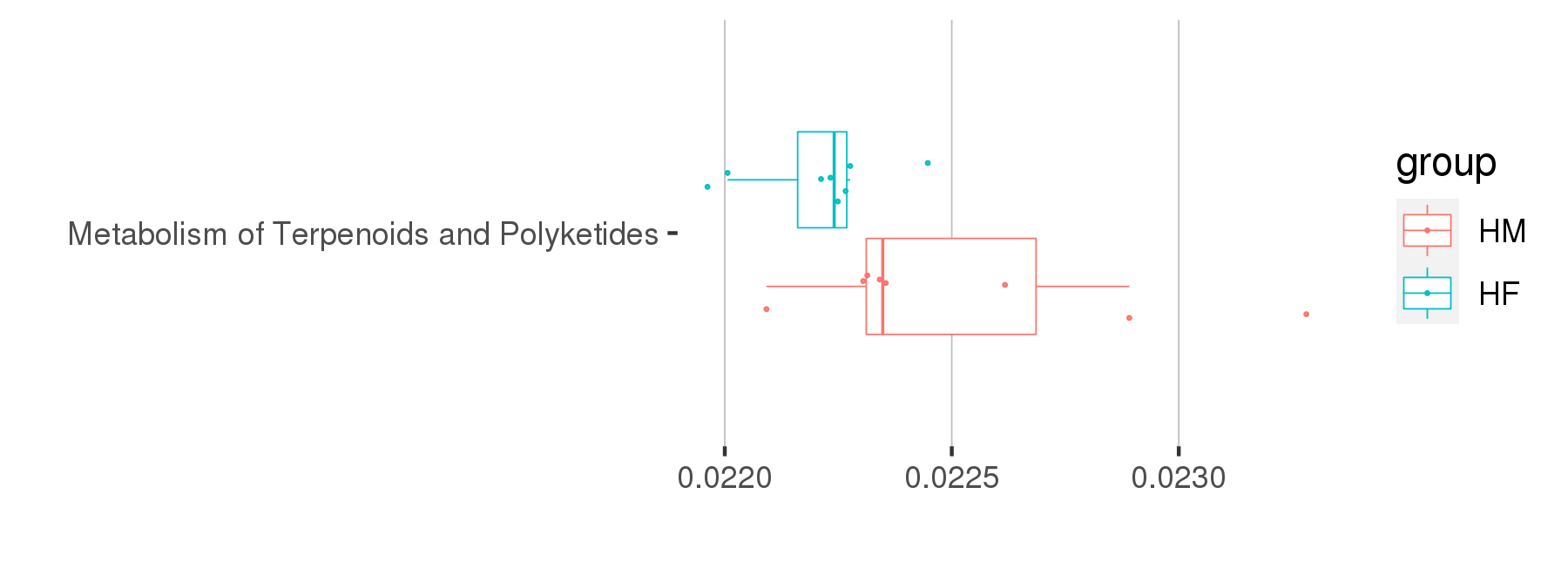

Supplement: Supplementary file 1 [file biology-12-00212-s001.zip › 16s rDNA SEQ/4.Tax4Fun/pathway_diff_L2/HM_vs_HF.wilcox.boxplot.png]

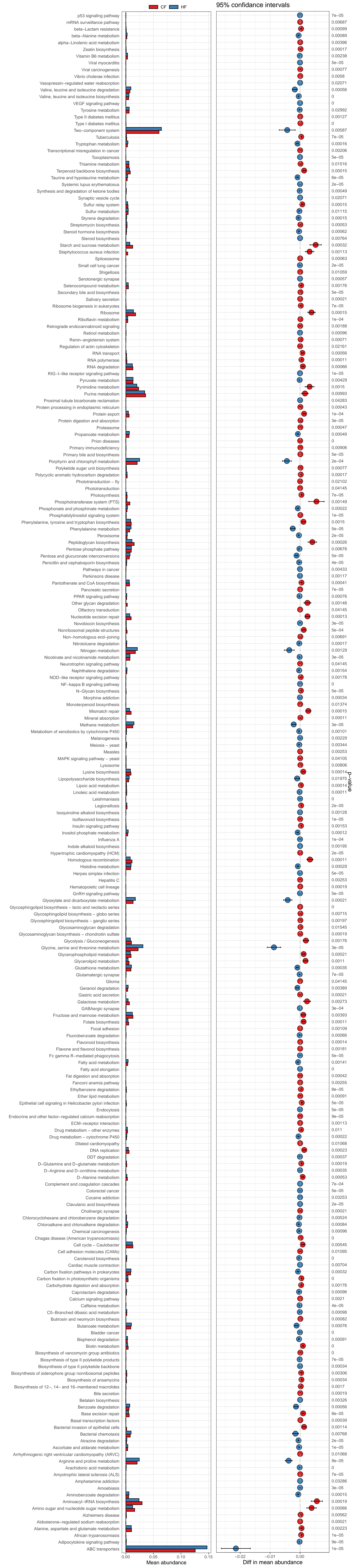

Supplement: Supplementary file 1 [file biology-12-00212-s001.zip › 16s rDNA SEQ/4.Tax4Fun/pathway_diff_L3/CF_vs_HF.t-test.extended_error_bar.pdf]

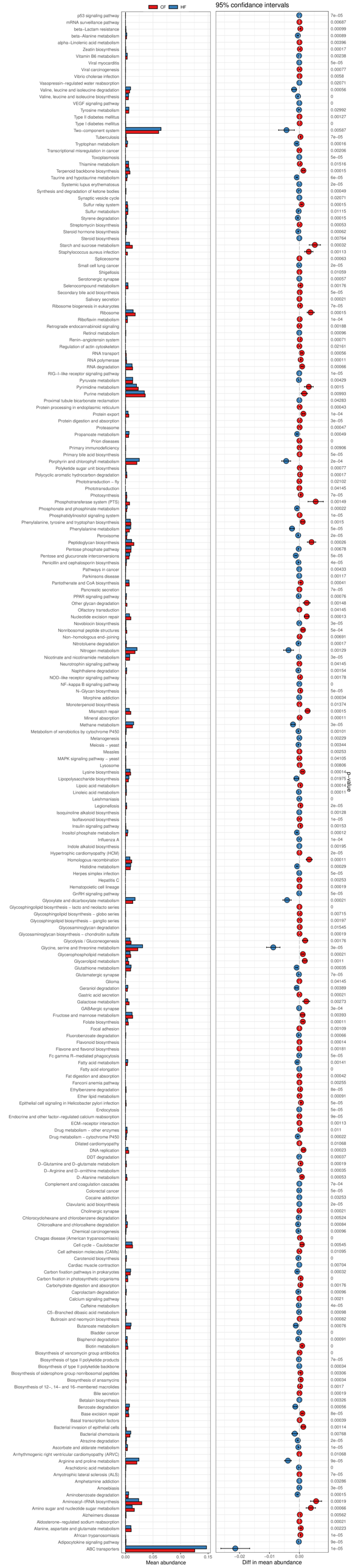

Supplement: Supplementary file 1 [file biology-12-00212-s001.zip › 16s rDNA SEQ/4.Tax4Fun/pathway_diff_L3/CF_vs_HF.t-test.extended_error_bar.png]

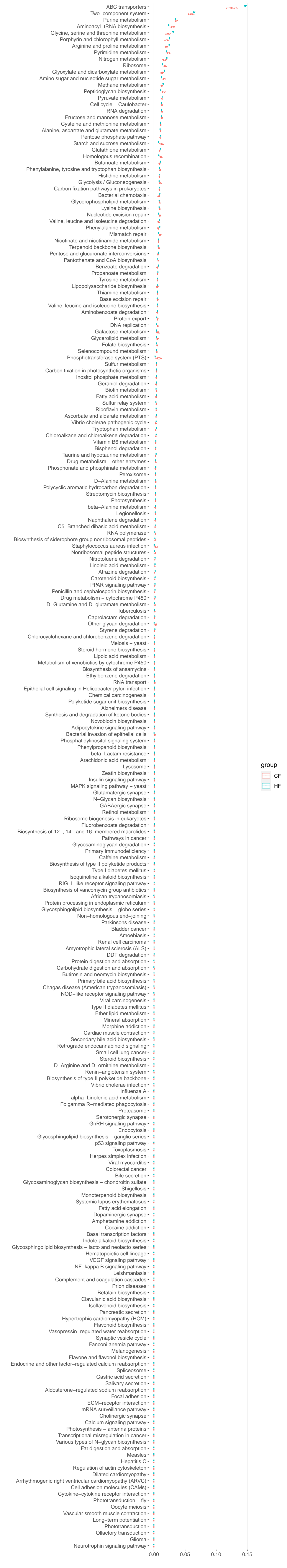

Supplement: Supplementary file 1 [file biology-12-00212-s001.zip › 16s rDNA SEQ/4.Tax4Fun/pathway_diff_L3/CF_vs_HF.wilcox.boxplot.pdf]

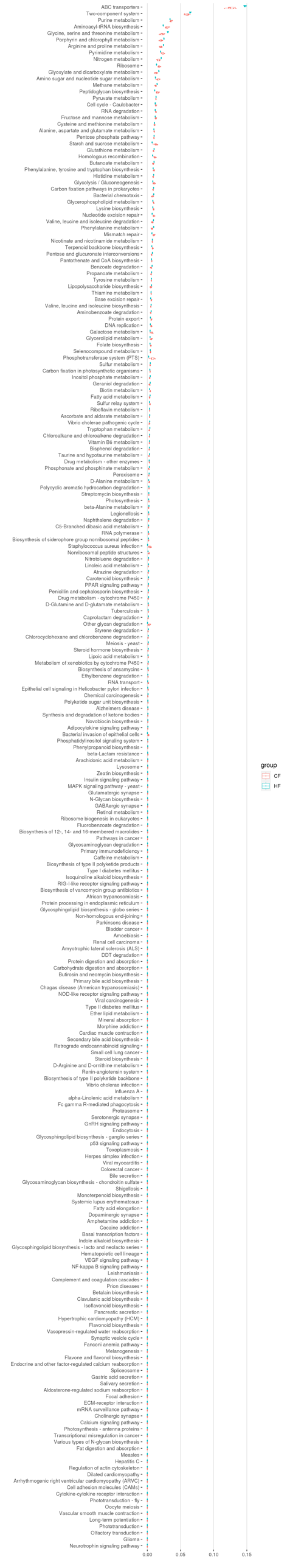

Supplement: Supplementary file 1 [file biology-12-00212-s001.zip › 16s rDNA SEQ/4.Tax4Fun/pathway_diff_L3/CF_vs_HF.wilcox.boxplot.png]

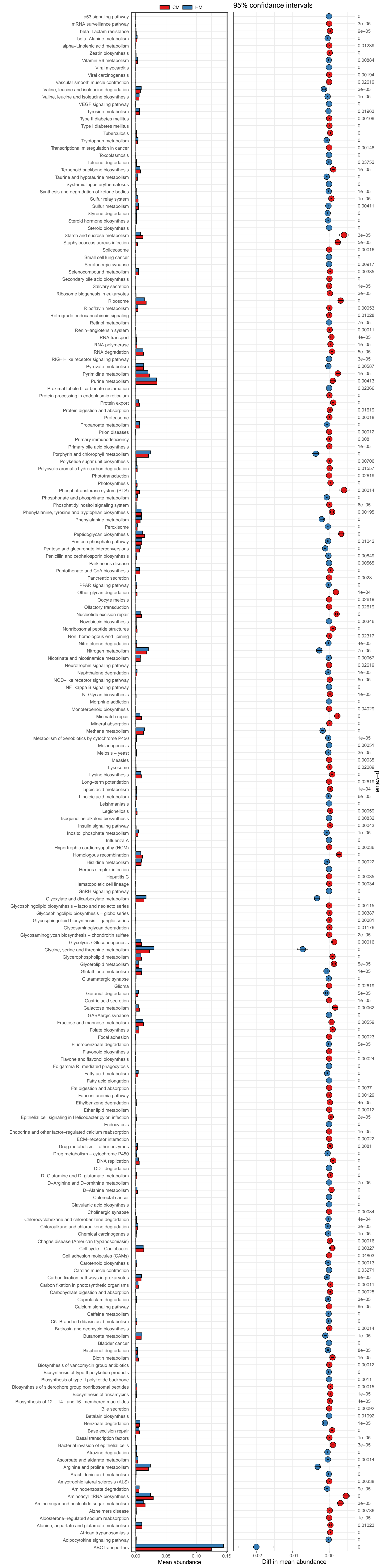

Supplement: Supplementary file 1 [file biology-12-00212-s001.zip › 16s rDNA SEQ/4.Tax4Fun/pathway_diff_L3/CM_vs_HM.t-test.extended_error_bar.pdf]

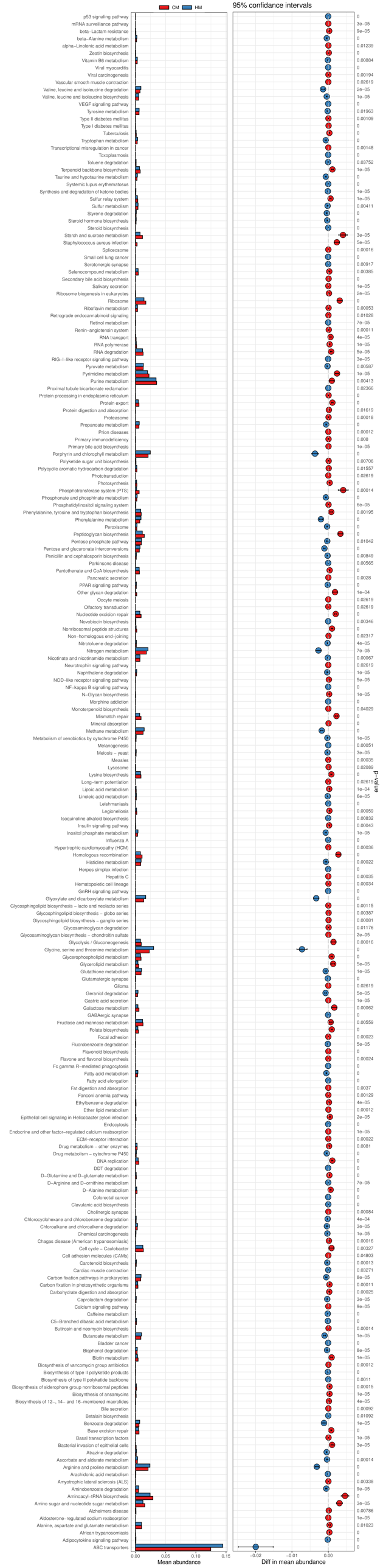

Supplement: Supplementary file 1 [file biology-12-00212-s001.zip › 16s rDNA SEQ/4.Tax4Fun/pathway_diff_L3/CM_vs_HM.t-test.extended_error_bar.png]

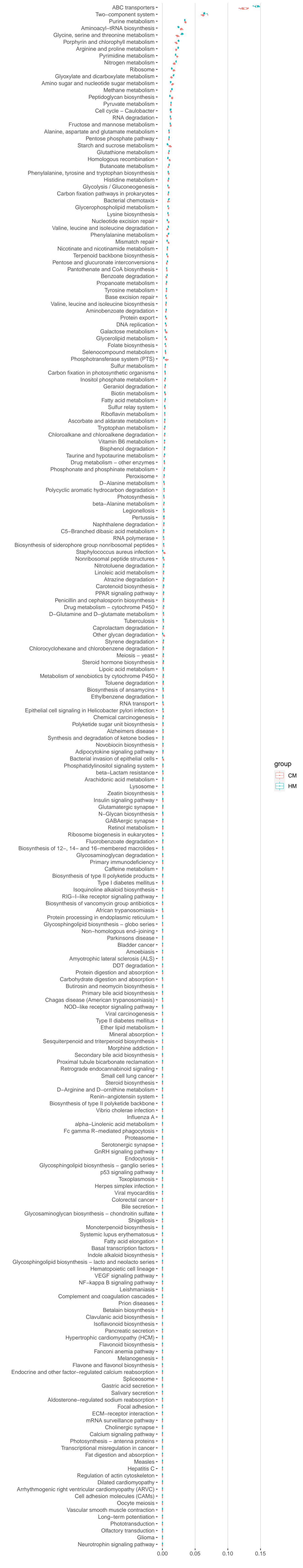

Supplement: Supplementary file 1 [file biology-12-00212-s001.zip › 16s rDNA SEQ/4.Tax4Fun/pathway_diff_L3/CM_vs_HM.wilcox.boxplot.pdf]

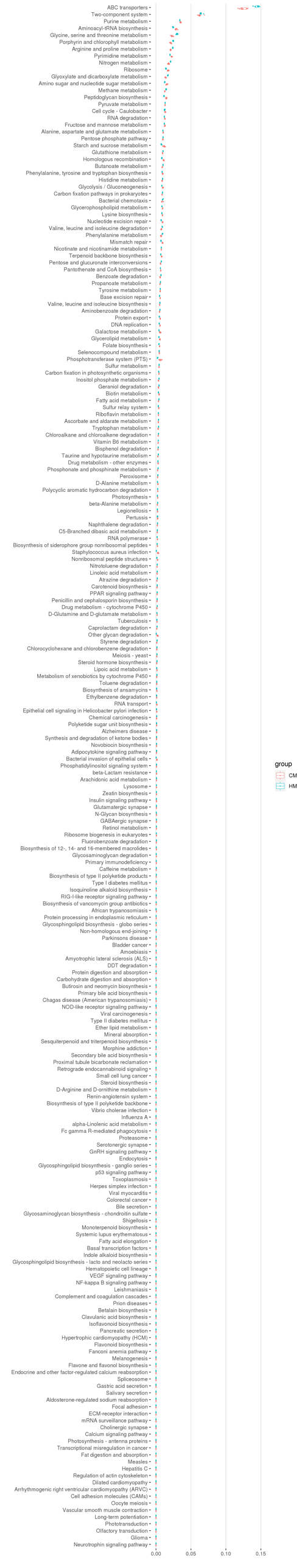

Supplement: Supplementary file 1 [file biology-12-00212-s001.zip › 16s rDNA SEQ/4.Tax4Fun/pathway_diff_L3/CM_vs_HM.wilcox.boxplot.png]

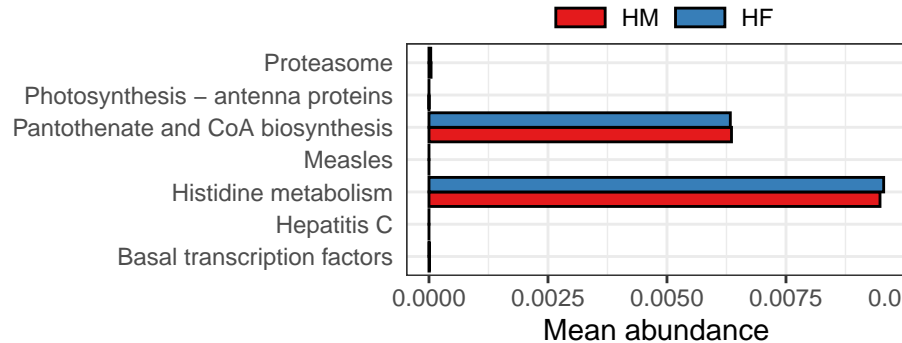

## 95% confidence intervals

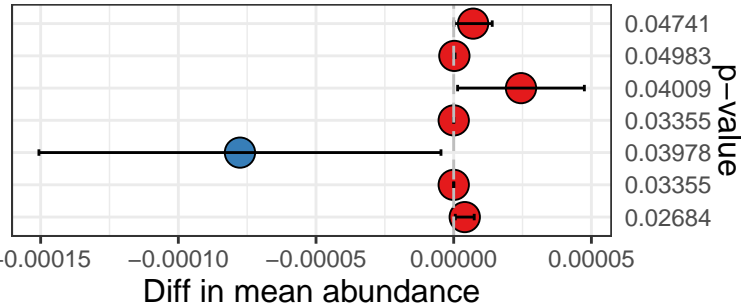

Supplement: Supplementary file 1 [file biology-12-00212-s001.zip › 16s rDNA SEQ/4.Tax4Fun/pathway_diff_L3/HM_vs_HF.t-test.extended_error_bar.pdf]

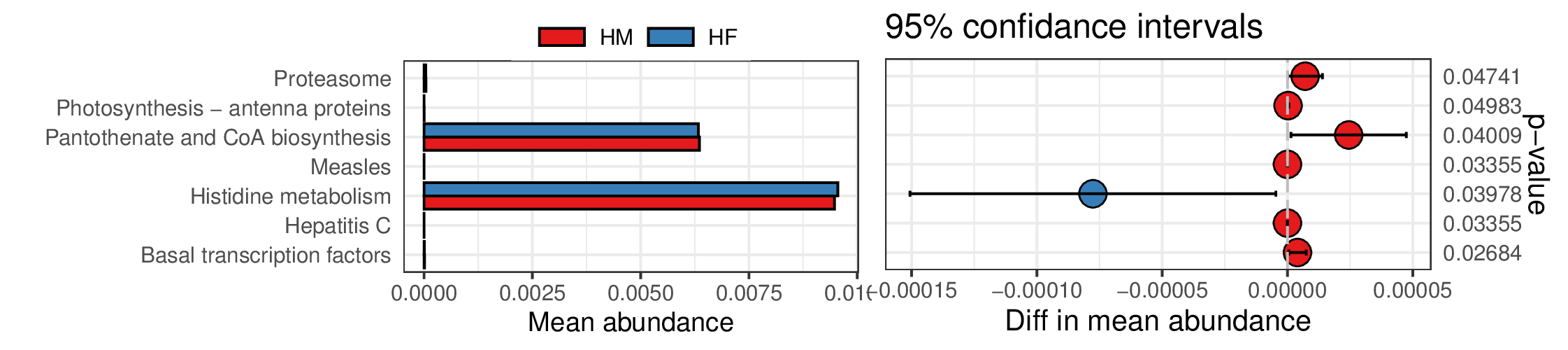

Supplement: Supplementary file 1 [file biology-12-00212-s001.zip › 16s rDNA SEQ/4.Tax4Fun/pathway_diff_L3/HM_vs_HF.t-test.extended_error_bar.png]

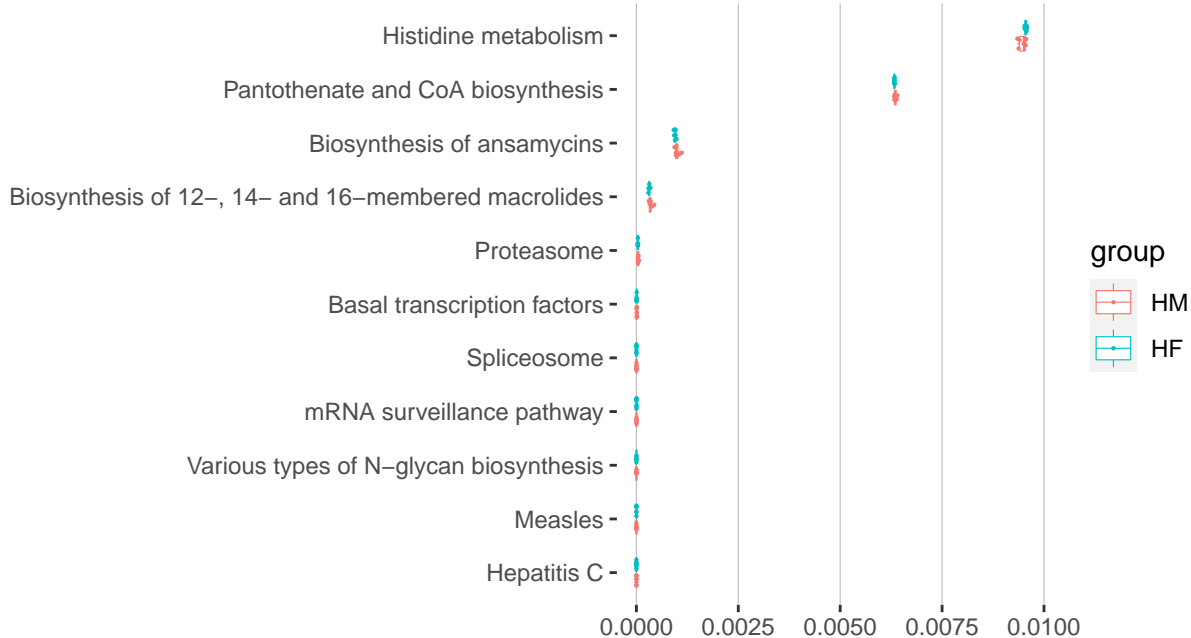

Supplement: Supplementary file 1 [file biology-12-00212-s001.zip › 16s rDNA SEQ/4.Tax4Fun/pathway_diff_L3/HM_vs_HF.wilcox.boxplot.pdf]

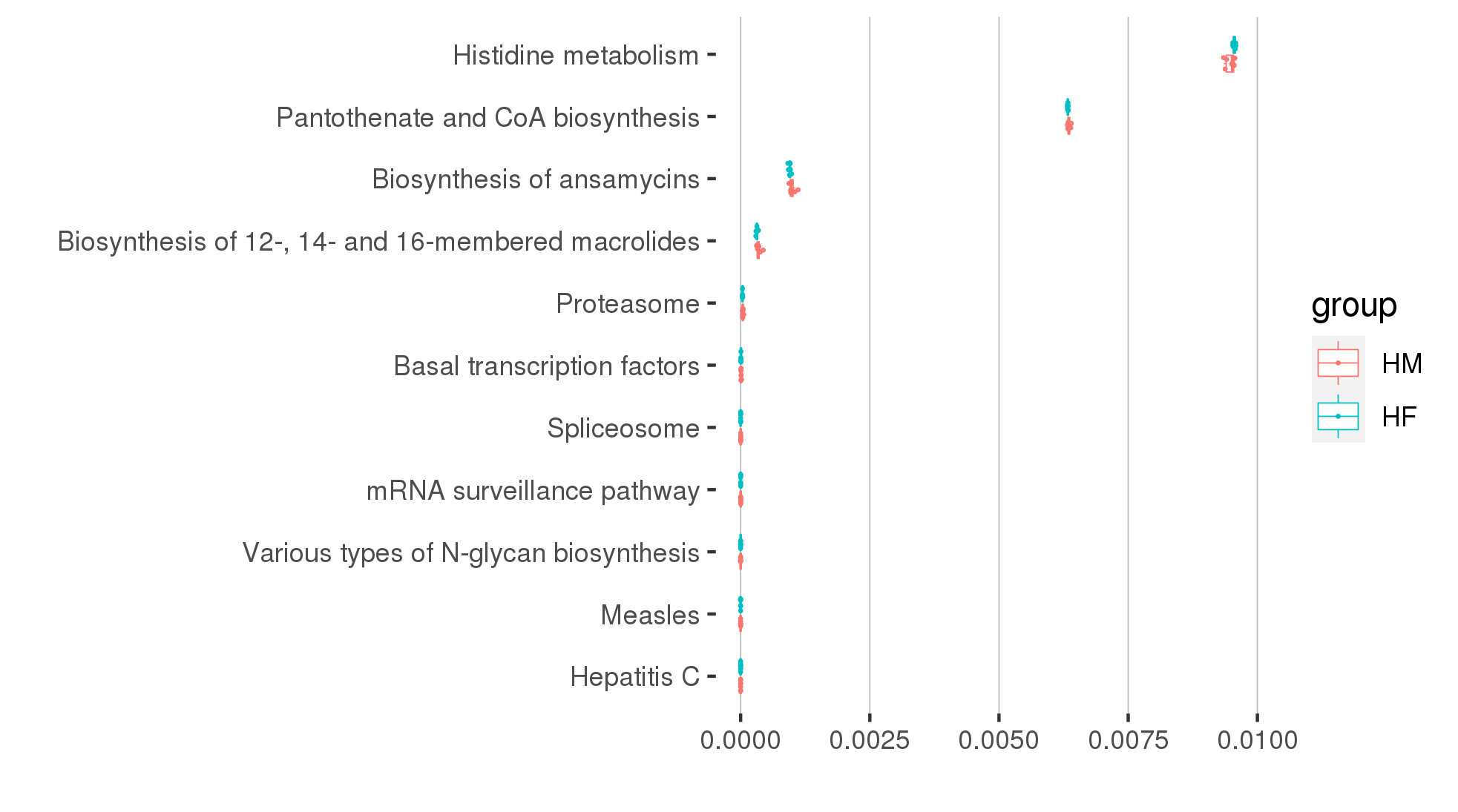

Supplement: Supplementary file 1 [file biology-12-00212-s001.zip › 16s rDNA SEQ/4.Tax4Fun/pathway_diff_L3/HM_vs_HF.wilcox.boxplot.png]
